# Unprecedented Diversity of the Glycoside Hydrolase Family 70: A Comprehensive Analysis of Sequence, Structure, and Function

**DOI:** 10.1021/acs.jafc.4c04807

**Published:** 2024-07-18

**Authors:** Tjaard Pijning, Lubbert Dijkhuizen

**Affiliations:** † Biomolecular X-ray Crystallography, Groningen Biomolecular Sciences and Biotechnology Institute (GBB), University of Groningen, Nijenborgh 7, Groningen 9747 AG, The Netherlands; ‡ Microbial Physiology, Groningen Biomolecular Sciences and Biotechnology Institute (GBB), University of Groningen, Nijenborgh 7, Groningen 9747 AG, The Netherlands; § CarbExplore Research B.V., Zernikelaan 8, Groningen 9747 AA, The Netherlands

**Keywords:** glycoside hydrolase family 70 (GH70), glucansucrase, branching sucrase, α-glucanotransferase, auxiliary domains

## Abstract

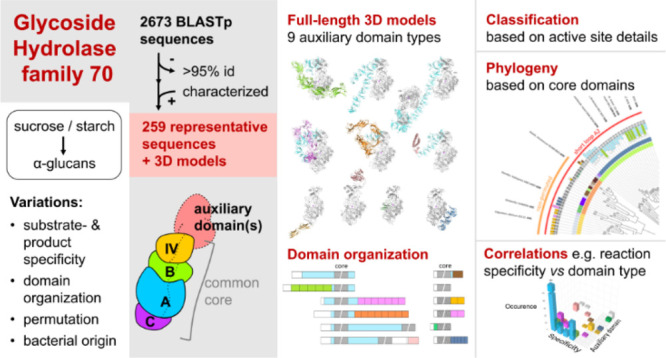

The glycoside hydrolase family 70 (GH70) contains bacterial extracellular multidomain enzymes, synthesizing α-glucans from sucrose or starch-like substrates. A few dozen have been biochemically characterized, while crystal structures cover only the core domains and lack significant parts of auxiliary domains. Here we present a systematic overview of GH70 enzymes and their 3D structural organization and bacterial origin. A representative set of 234 permuted and 25 nonpermuted GH70 enzymes was generated, covering 12 bacterial families and 3 phyla and containing 185 predicted glucansucrases (GS), 15 branching sucrases (BrS), 8 “twin” GS-BrSs, and 51 α-glucanotransferases (α-GT). Analysis of AlphaFold models of all 259 entries showed that, apart from the core domains, the structural variation regarding auxiliary domains is far greater than anticipated, with nine different domain types. We analyzed the phylogenetic distribution and discuss the possible roles of auxiliary domains as well as possible correlations between enzyme specificity, auxiliary domain type, and bacterial origin.

## 1 Introduction

Glucansucrase (GS) enzymes catalyze the cleavage of sucrose into fructose and glucose, with the concomitant transfer of the glucose residue to a growing α-glucan polymer. GSs were identified about four decades ago as being responsible for the synthesis of cariogenic carbohydrate polymers by *Streptococcus* species.^1^ Sequence analysis revealed significant homology to GH13 α-amylases;^2^ however, featuring a circularly permuted catalytic domain, GSs were classified as a new glycoside hydrolase family GH70 in the late 90s. Together with GH13 and GH77 enzymes, they were assigned to the GH-H clan of related families.^3,4^ Later, GH70 GS were identified in several other bacterial species, but only in Lactic Acid Bacteria (LAB). GS enzymes produce α-glucans varying in glycosidic linkages, degree of branching, and sizes.^1,5^ Their most well-known α-glucan products are dextrans (with α-1,6 glycosidic linkages), but also mutans (α-1,3), reuterans (α-1,4/α-1,6) or alternans (alternating α-1,3 and α-1,6) may be produced, depending on the enzyme product specificity. In addition, so-called branching sucrases (BrS) have been identified,^6,7^ using sucrose as donor substrate to introduce α-1,2 or α-1,3 branchpoints in a (linear) dextran acceptor substrate. These BrS enzymes are either found as separate proteins, or as part of large “twin” GS-BrS enzymes with both a dextransucrase catalytic domain and a branching sucrase catalytic domain. The variation in α-glucan structures also results in significant differences in their physicochemical properties.^1,8−11^

**Figure 1:**
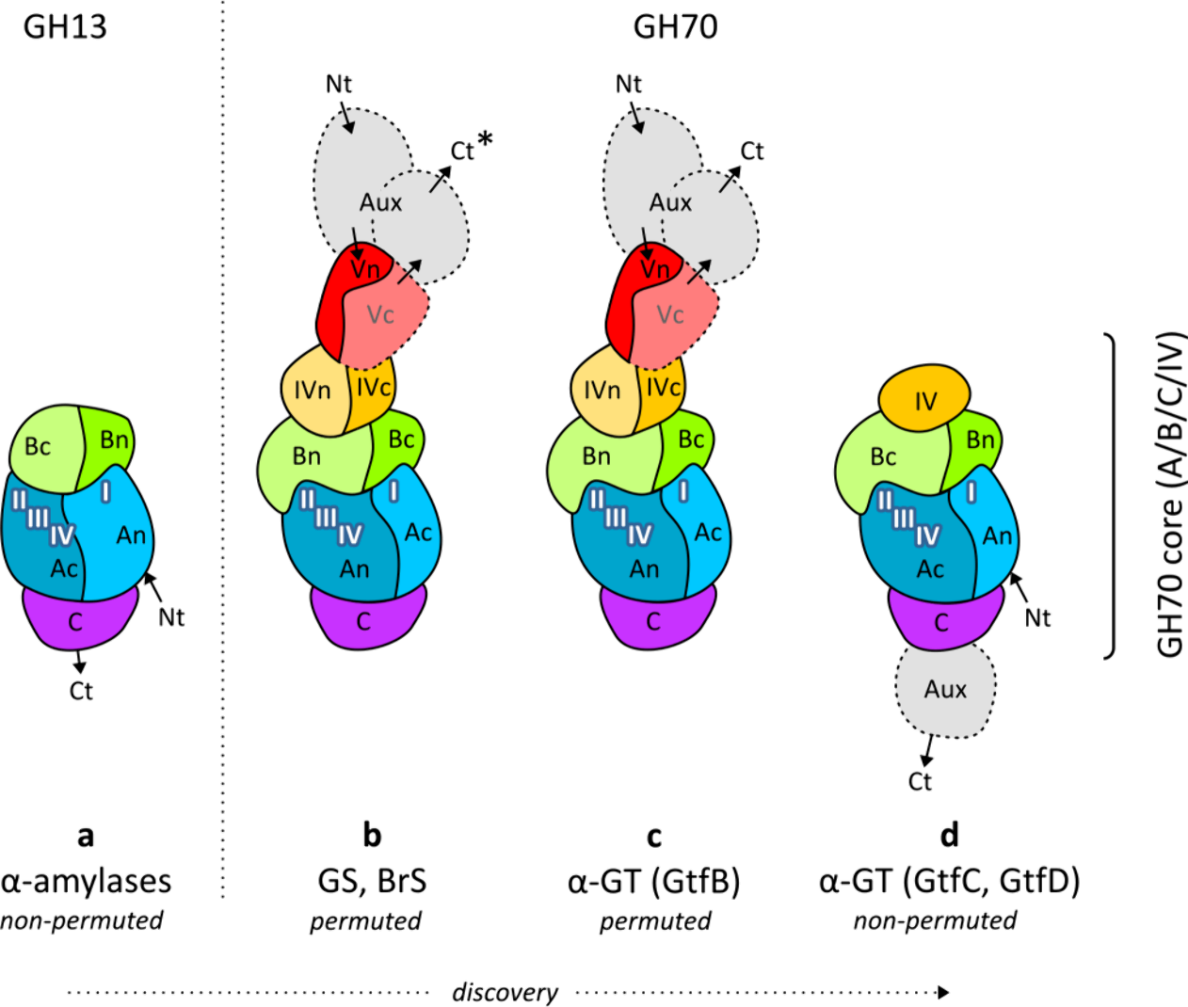
Schematic domain organization of GH13 and GH70 enzymes, in the order of discovery. The GH70 enzymes share domains A, B and C with GH13 but have an additional domain IV; domains A/B/C/IV thus constitute the common core of GH70 enzymes. Furthermore, GH70 enzymes have auxiliary domains (“Aux”, depicted in gray) of which domain V, depicted in red and first observed in glucan- and branching sucrases (GS and BrS, respectively), can be considered an example. For GS, BrS and GtfB-type α-glucanotransferases (α-GT), circular permutation results in a different order of the conserved sequence motifs I–IV along the polypeptide chain, and in a “U-shape” domain sequence with domain C at the bottom of the U. Note that the “twin” GS-BrS enzymes are not fully depicted in this figure; they feature a second A/B/C/IV core attached to the C-terminus of the GS entity, indicated by the star (*). The N- and C-termini are indicated (Nt, Ct); note that in permuted enzymes, auxiliary domains occur both N- and C-terminally, but only C-terminally in nonpermuted enzymes. See also Figure S2 for the domain organization of individual GH70 enzymes in our set.

Glucansucrases are generally large, extracellular proteins with sizes of 140–200 kDa. Aiming for higher protein expression levels, protein sizes were reduced by using severely N- and/or C- terminally truncated constructs that lacked significant parts of noncore (auxiliary) domains. This allowed successful crystallization of several GH70 GSs, resulting in a number of high-resolution 3D structures.^12−18^ The first glucansucrase 3D structure was reported for the *Limosilactobacillus reuteri* 180 Gtf180 enzyme (LrGtf180^12^), revealing five distinct, linearly arranged domains. Three of these domains structurally align with the A, B, and C domains of family GH13 α-amylases and therefore were named accordingly (Figure 1a, b). The other two domains did not show structural similarity to domains occurring in the GH13 family and were thus named domains IV and V. Subsequently determined 3D structures of GSs showed similar topologies for domain IV and V.^13−18^ Currently, the CAZy database (https://www.cazy.org)^1,5^ lists >1000 annotated GH70 enzymes.^2^

More recently, the history of GH70 enzyme discovery took a new turn by the finding of starch-acting α-glucanotransferases (α-GT),^19^ thus representing a second substrate specificity within the family, designated as GtfB. While sharing the same domain organization (Figure 1, panel c), unlike GSs, these enzymes are inactive with sucrose; instead, they use starch/amylopectin and/or amylose and maltooligosaccharides as substrate, cleaving α-1,4 linkages and subsequently introducing (via transglycosylation) α-1,6 linkages in linear chains and/or at branchpoints (4,6-α-GT). After the GtfB subfamily, two more GH70 α-GT subfamilies were recognized and designated as GtfC and GtfD, depending on their amino acid sequences and structural features (Figure 1, panel d).^20−22^ Most of the characterized subfamily GtfB α-GT enzymes are from LAB; in contrast, subfamily GtfC enzymes were found in other Gram-positive bacteria (and not in LAB), and subfamily GtfD enzymes were found in both Gram-positive (non-LAB) and Gram-negative bacteria. Similar to GS and BrS, to study α-GTs, their protein sizes have been reduced by using N- and C-terminally truncated constructs that lack significant parts of noncore (auxiliary) domains. Crystal structures have been reported for GtfB-type 4,6-α-GTs from four different *Limosilactobacillus* species,^23−26^ as well as for the GtfC-type 4,6-α-GT from *Geobacillus* 12AMOR1.^27^

Based on phylogenetic relations, it is our current understanding that the GH70 subfamilies have evolved from the GH13 starch-acting enzymes.^20−22^ Notably, enzymes of family GH13 and GH70 are structurally related: both feature the catalytic domain A containing a (β/α)_8_-barrel topology, as well as domains B and C. There is a clear sequence similarity in these 3 domains, with members of both families containing a number of conserved sequence motifs that include the catalytic residues responsible for substrate cleavage and transglycosylation.^1,28^ However, compared to GH13, GH70 subfamilies GtfC and GtfD have gained an extra domain IV,^27,26^ whereas subfamily GtfB and GS/BrS enzymes in addition gained an extra domain V (Figure 1).^22^ Finally, apart from the different domain organization, there is another feature that divides GH70 enzymes in two subgroups. While GH70 GtfC/GtfD share the same nonpermuted “domain sequence” observed in GH13 and GH77 (belonging to the same GH-H clan), the GH70 GS/BrS and GtfB-type α-GT enzymes have undergone a so-called circular permutation in their core domains. This was first recognized in the GH70 GS sequences^2^ and later confirmed by the first GH70 crystal structure of LrGtf180 glucansucrase;^12^ as a result of this permutation, in the catalytic domain A, the order of the conserved sequence motifs is II - III - IV - I (instead of I - II - III - IV).

Many carbohydrate-active enzymes are modular; besides a catalytic domain they often contain other functional entities such as carbohydrate-binding modules (CBM) and/or linker domains. Likewise, GH70 enzymes are known to possess N- and C-terminal extensions attached to the common core domains A/B/C/IV (auxiliary domains; gray in Figure 1). These extensions often are of considerable length (several hundreds of amino acid residues), and it was recognized early that they frequently contain different types of repeating sequence motifs.^29−32^ The exact role of the auxiliary domains remained somewhat elusive, although studies on auxiliary domains of different GH70 enzymes suggested a possible role in α-glucan binding.^16,31,33−37^ Notably, all so far published 3D structures of GH70 GS/BrS enzymes showed that their domain V (which can be considered an auxiliary domain; red in Figure 1b, c) takes up a β-solenoid topology.^12−18^ Whether this applies to all GH70 enzymes is so far unknown, but 3D modeling of some GtfC-type α-GTs suggested that other topologies are likely to exist in auxiliary domains.^26^

The last decades have seen a growing interest in the α-glucan products of GH70 enzymes, which can be synthesized from relatively cheap substrates in biobased, eco-friendly routes.^8,10,11^ Due to the promiscuity of GH70 enzymes regarding the acceptor reaction, a wide range of glucosylated sugars or hydroxylated compounds can be synthesized efficiently. Varying in structure and physicochemical properties, α-glucans and other transglucosylation products have already found numerous applications in nutrition, health, medicine, cosmetics, and as biomaterials.^1,7−11^ Importantly, the potential prebiotic properties of α-glucans and the possibility to synthesize low glycemic index sweeteners hold great promise for GH70 enzymes. In this light, understanding the underlying mechanisms and reaction specificities of GH70 enzymes is crucial to advance the development of their application. Given the sequence-, topological-, catalytic- (specificity) and structural diversity of GH70 enzymes, we set out to systematically characterize this large family, using bioinformatics, phylogenetics and 3D modeling. Importantly, recent advances in structure prediction from protein sequence (AlphaFold^38^) for the first time facilitated a comprehensive “3D survey” revealing enormous variation, especially regarding the auxiliary domains in this family, also allowing us to discuss their possible role in more detail than before.

## 2 Materials and Methods

### 2.1 Creating a Representative Set of Sequences

**Figure 2:**
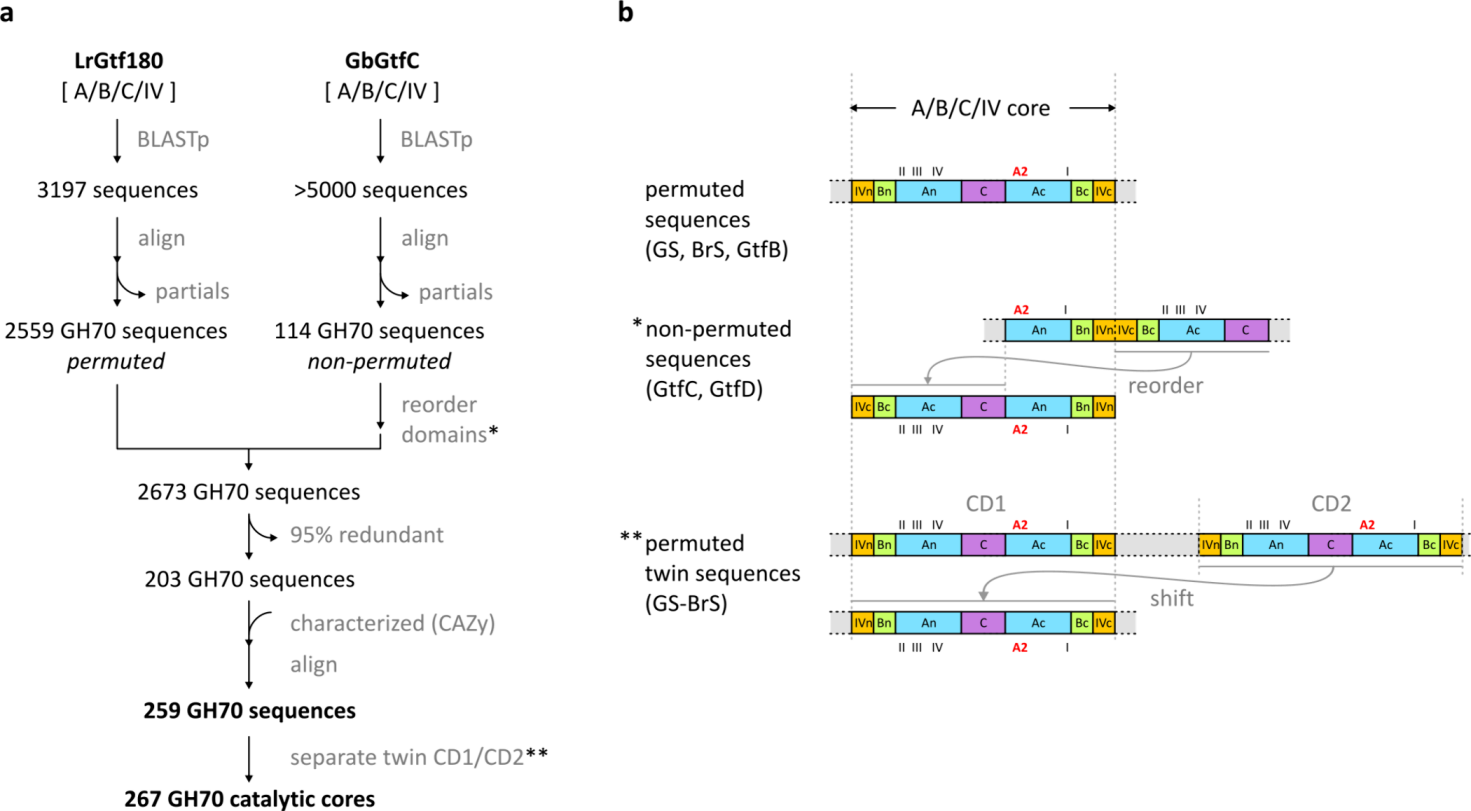
Strategy for creating a representative multiple sequence alignment of GH70 enzymes. (a) Flow scheme used to combine permuted (left) and nonpermuted (right) sequences, respectively represented by glucansucrase Gtf180 from *L. reuteri* 180 (LrGtf180)^12^ and 4,6-α-glucanotransferase GtfC from *Geobacillus* 12AMOR1 (GbGtfC).^26^ (b) Organization and reordering of domains A, B, C and IV in permuted and nonpermuted sequences to allow their alignment. In the domain names, the lowercase character denotes whether it is an N- or C-terminal segment (e.g., An is the N-terminal segment of domain A). For twin sequences, the two catalytic cores were separated and the CD2 was aligned with CD1. For nonpermuted sequences, the polypeptide segment containing domains IVc-Bc–Ac-C was selected and placed N-terminal of the segment containing domains An-Bn-IVn in order to match the organization of permuted enzymes. The position of the GH70 conserved sequence motifs I to IV is indicated, as well as that of Loop A2 (red).

In order to take into account that GH70 contains permuted as well as nonpermuted sequences, we performed two separate BLASTp runs that were then combined to obtain a single multiple sequence alignment; a schematic overview of this strategy is depicted in Figure 2a. First, the sequence of *Limosilactobacillus reuteri* 180 Gtf180 glucansucrase (UniProt entry Q5SBM0; LrGtf180) was trimmed to the region corresponding to domains A, B, C and IV (residues 791–1639) based on its crystal structure;^12^ this region, hereafter referred to as the A/B/C/IV core, was then used for a BLASTp search (January 2023). The resulting hits were aligned with MAFFT,^39^ using the option to base the alignment on the same region. This first set represents the circularly permuted GH70 enzymes; after removal of partials (lacking significant core parts) it contained 2559 sequences. For the second set, a similar workflow was applied with the nonpermuted *Geobacillus* 12AMOR1 GtfC 4,6-α-glucanotransferase sequence (AKM18207.1; GbGtfC) trimmed to domains A, B, C and IV (residues 33–738); the resulting aligned set contained 114 sequences. Representing nonpermuted GH70 enzymes, we reordered these by swapping their N- and C-terminal halves (Figure 2b) to allow alignment with the permuted set.

The two sets of sequences were combined to a single set of 2673 GH70 sequences, which was reduced to 203 by applying a redundancy limit of 95% (sequence identity). We then added sequences from the CAZy GH70 page (http://www.cazy.org/GH70; December 2023) for characterized enzymes, if not already present, and realigned the set. One sequence (a putative GS) was annotated as obsolete in the NIH Protein Database and was removed. Furthermore, the set contained 15 partial sequences, lacking N- or C-terminal segments; these were either replaced by the most identical full sequence from the same bacterial species if the sequence identity was >95% (11 cases), or else kept (4 cases).

The resulting final set, listed in Table S1, contained 259 GH70 sequences: 234 natively permuted and 25 nonpermuted reordered ones.

### 2.2 Sequence Alignment and Phylogenetic analysis

The 259 representative GH70 sequences were retrieved from UniProt (https://www.uniprot.org/) or NCBI Protein Database (https://www.ncbi.nlm.nih.gov/protein/). After combining the permuted and nonpermuted set (see above), they were realigned with MAFFT, again using the A/B/C/IV core as selected region for alignment. Notably, among the 259 sequences, 8 significantly longer “twin” sequences with two such regions were found, for which the two A/B/C/IV cores (CD1, CD2) were extracted and aligned separately (Figure 2b). The final alignment, thus containing 267 aligned catalytic GH70 cores, was analyzed within JalView^40^ to assess sequence identity (with respect to LrGtf180), the length of active site loops A1, A2 and B,^22^ and the residues constituting GH70 conserved motifs, in particular motif III.^5^ Sequence repeats (in auxiliary domains) were detected with the RADAR Web server;^41^ auxiliary domain alignments were performed with Clustal^42^ or MUSCLE;^43^ alignment figures were prepared using ESPript output.^44^ Sequence logos were generated with WebLogo.^45^ A phylogenetic tree of the 267 GH70 cores was constructed within MEGA X using the Maximum Likelihood method.^46^

### 2.3 Structural Analysis

The CAZy database currently lists experimental 3D structures (crystal structures) of 13 different GH70 N- or C-terminally truncated enzymes. In order to obtain more complete 3D models, we either retrieved these from the AlphaFold Protein Structure Database (https://alphafold.ebi.ac.uk^47^) or calculated them locally with AlphaFold2.^38^ Longer sequences (>1700 residues) were first split in an N- and a C-terminal segment with sufficient overlap and then recombined. In some cases, this approach resulted in clashing auxiliary domains; although such a 3D model does not represent a feasible structure, it can still be used to analyze the individual domain folds. The predicted domain organization of each of the 259 models was guided by structural superposition of the segments corresponding to the A/B/C/IV core of LrGtf180 (PDB: 3KLK(12)).

In general, the pLDDT scores of the AlphaFold models were sufficiently high (>60) to reliably predict the structures of almost the complete enzymes, including the core A/B/C/IV domains as well as large parts of auxiliary domains, if present. The majority of (predicted) glucansucrase and branching sucrase AlphaFold models also showed segments with low confidence, in particular at the N-terminus of auxiliary domains; segments with a pLDDT score lower than ≈60 were not considered in structural analyses. The fold/topology of N- and C-terminal auxiliary domains was extracted from the model and analyzed by FoldSeek^48^ or PDBeFold,^49^ focusing on resulting homologues with the highest E-value and sequence identity. Auxiliary domain topologies were compared with those from InterPro/Pfam databases.^50^

## 3 Results

Using the sequences of the glucansucrase Gtf180 from *Limosilactobacillus reuteri* 180 (LrGtf180) and the 4,6-α-glucanotransferase GtfC from *Geobacillus* 12AMOR1 (GbGtfC) as BLASTp queries for permuted respectively nonpermuted enzymes, we obtained a set of 2673 GH70 sequences. After applying core reordering, a 95% redundancy filter, and addition of biochemically characterized enzymes, a representative set of 259 GH70 enzymes was obtained (Figure 2) which was then analyzed regarding bacterial origin, sequence features (permutation, motifs, active site loops), phylogenetic relations and 3D structure (core domains, auxiliary domains, active site loops).

### 3.1 Bacterial Origin

The majority (175; 67.6%) of the 259 representative GH70 sequences originates from the Lactobacillaceae bacterial family, mainly including *Lactobacillus, Leuconostoc, Oenococcus* and *Weissella,* genera in which GH70 enzymes have been identified before (Table S1). GH70 enzymes now also were observed in other genera in this family, namely in *Periweissella* (isolated from fermented cassava), *Convivina* (an insect gut symbiont), as well as in some fructophilic LAB (FLAB) such as *Fructobacillus* (flower) and *Nicoliella* (honey). A second well represented bacterial family is the Streptococcaceae family (57 entries; 21.9%) exclusively containing GH70 enzymes from *Streptococcus* species, also containing several characterized ones (Table S1). The remaining 27 sequences (10.4%) are from families in the same Bacillota phylum (Paenibacillaceae, Enterococcaceae, Sporolactobacillaceae), the Pseudomonadota phylum (Burkholderiaceae, Oceanospirilliceae, Pseudomonadaceae, Rhodanobacteraceae) or the Actinomycetota phylum (Microbacteriaceae, Propionibacteriaceae). Not reported before, we found GH70 enzymes in *Enemella evansiae*, isolated from human clinical samples, and *Naumanella* species, both belonging to the Propionibacteriaceae phylum. Together, our set contains GH70 enzymes from 3 different phyla and 12 families, covering very different habitats: host digestive tract (oral cavity, stomach or gut), fermented food, soil, marine, honey, flowers or even human skin. Moreover, while most of the species live in moderate temperatures, some of them adapted to extreme low or high temperatures (e.g., *Exiguobacterium* and *Geobacillus* spp. found in permafrost or hydrothermal vents).^21,51^

### 3.2 Sequence Identity, Permutation and Length

**Figure 3:**
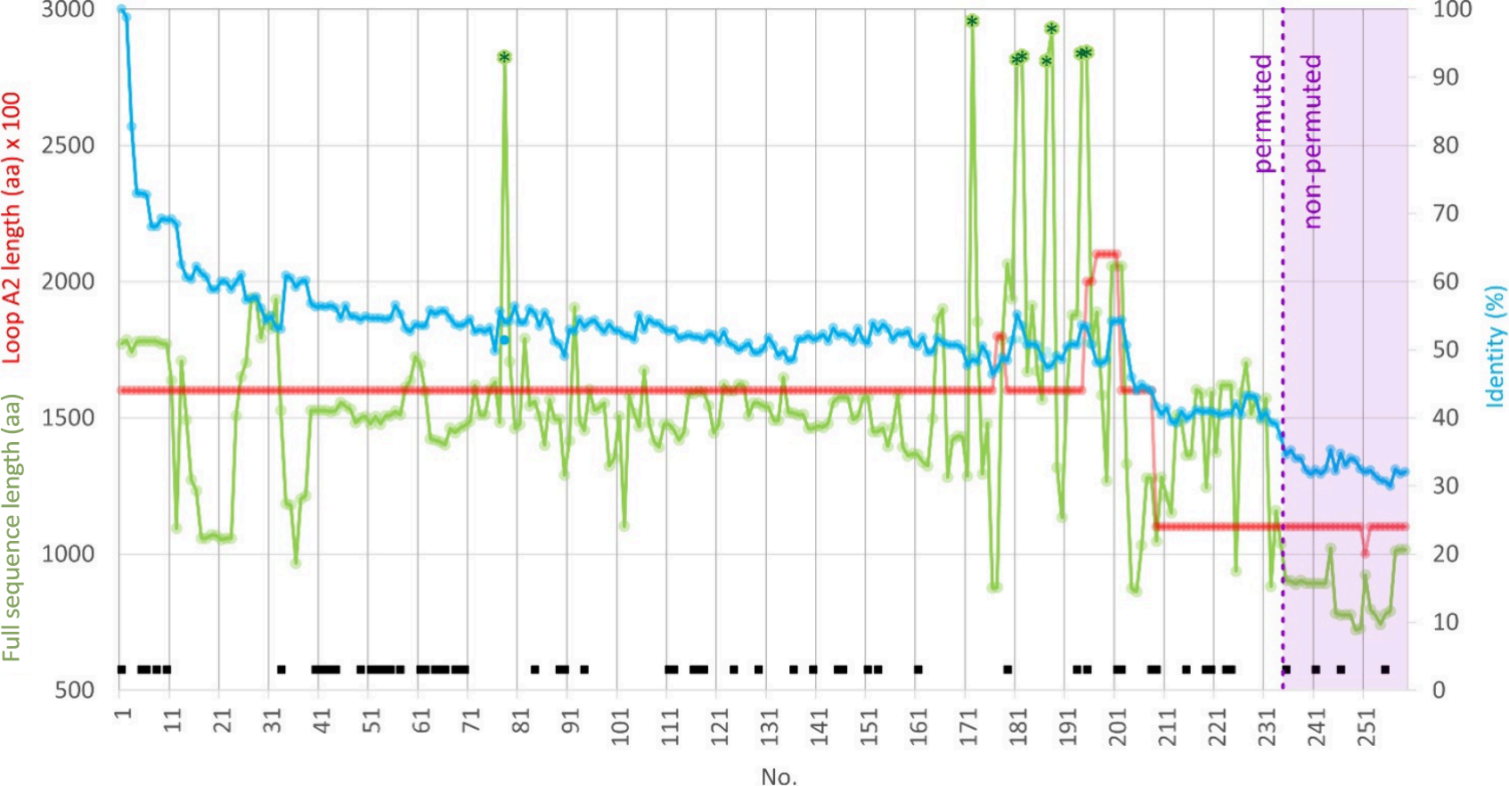
Sequence length (full sequence; green), sequence identity (A/B/C/IV core; cyan) and length of loop A2 (red) in the 259 GH70 sequences. Eight significantly longer sequences are annotated with stars, and characterized enzymes with a black square; nonpermuted enzymes (no. 235–259) are indicated by violet shading.

In the set of 259 GH70 sequences, the sequence identity of the A/B/C/IV core (with respect to that of LrGtf180) ranges from 98.8 to 28.8% (Figure 3); besides 234 permuted enzymes (90.3%) there are 25 nonpermuted enzymes (9.7%) which appear in the lowest sequence identity region. Only a few enzymes, mostly from *L. reuteri* species, have a sequence identity >70%, suggesting these have a rather unique sequence. On the other hand, for a large part of the set the sequence identity is between about 50 and 60%, and drops to 30–40% for the last ≈50 sequences of the set. There is a large variation in sequence length, from 721 to 2954 residues; the only apparent trend is that nonpermuted enzymes tend to be shorter. Four enzymes are marked as partial in sequence databases, but were kept because they still featured complete A/B/C/IV core domains (indicated with “(p)” in Table S1. Notably, eight sequences are significantly longer (2806–2954 residues) and contain two A/B/C/IV cores. Finally, it is worth mentioning that the so far characterized GH70 enzymes are well spread over the set.

### 3.3 Loop A2 Distinguishes GS/BrS from α-GT

**Figure 4:**
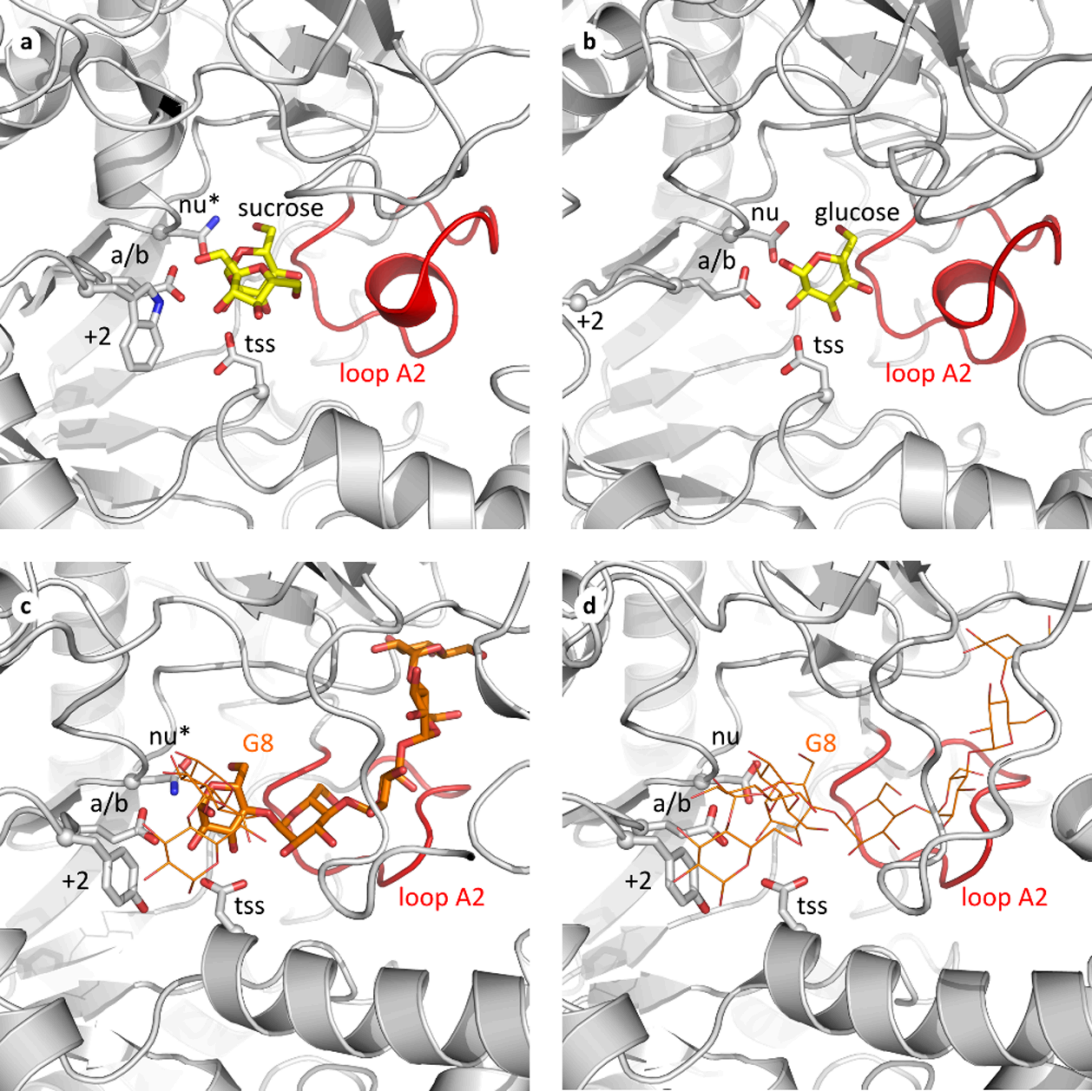
Structural superposition of GH70 active sites with Loop A2 (red) in a glucansucrase, a branching sucrase and two α-4,6-glucanotransferases. The three catalytic residues are shown as sticks: nu = nucleophile Asp (* indicates mutation to Asn), a/b = acid/base Glu, tss = transition state stabilizer Asp, as well as the Trp, Gly or Tyr (“+2”) two positions downstream of the acid/base residue. Experimentally observed ligands are shown as sticks; modeled ligands as lines. (a) Glucansucrase Gtf180 from *L. reuteri* 180 (PDB: 3HZ3(12)) complexed with the substrate sucrose (yellow carbon atoms). (b) Branching sucrase DSR-E from *L. mesenteroides* B-1299 (PDB: 4TVD(35)) complexed with glucose. (c) GtfB-type 4,6-α-glucanotransferase from *L. reuteri* 121 (PDB: 5JBF(22)) complexed with maltopentaose (orange carbon atoms) which was extended to maltooctaose (G8) by modeling. (d) GtfC-type 4,6-α-glucanotransferase from *Geobacillus* 12AMOR1 (PDB: 7ZC0(26)]) with a modeled maltooctaose.

Apart from permutation, a clear distinction was observed regarding the length of active site loop A2 (Figure 3; Figure S1), also dividing GH70 in two groups, different from the permutation division, but correlating with substrate specificity. Notably, loop A2 is not part of one of the seven GH13/GH70 conserved motifs,^1,22,52^ but lies in between motifs IV and I. In the top 208 entries of the aligned set, the length of this loop varies between 16 and 21 amino acid residues (mostly 16 residues). The characterized sequences in this first group are glucansucrases or branching sucrases; moreover, the available crystal structures of 8 enzymes from this group revealed that loop A2 features a short helix, blocking donor subsites beyond subsite −1 (Figure 4a, b). Consequently, the high sequence and length conservation of this loop (89% have a sequence identity >60%) in the 208 entries strongly suggest that all these enzymes share the same substrate specificity, using a single donor subsite (−1) and utilizing sucrose for polymerization or dextran as template for branch grafting. A few enzymes in this group have a somewhat longer loop A2, where the up to 5 extra residues precede the α-helix; however, the crystal structure of the *Leuconostoc mesenteroides* NRRL B-1355 alternansucrase (PDB: 6HVG(17)) shows that a longer loop A2 blocks donor subsites in a similar way.

The remaining 51 GH70 sequences consistently have a shorter loop A2, consisting of 11 amino acid residues, which partly differs in amino acid composition (Figure S1); since all enzymes from this group were characterized as α-glucanotransferases (α-GT), they likely share the same substrate specificity, utilizing starch-like oligosaccharides for α-glucan synthesis. Structurally, the shorter loop A2 near subsite −1 lacks a helical element (Figure 4c,d) resulting in multiple accessible donor subsites, as was experimentally observed in the 5 reported α-GT crystal structures.^22−26^ Two other loops (A1 and B) vary in length among α-GTs and affect the accessibility of the donor subsites in these enzymes,^23^ but the length and position of loop A2 in all α-GTs is very consistent.

### 3.4 Motif III

**Figure 5:**
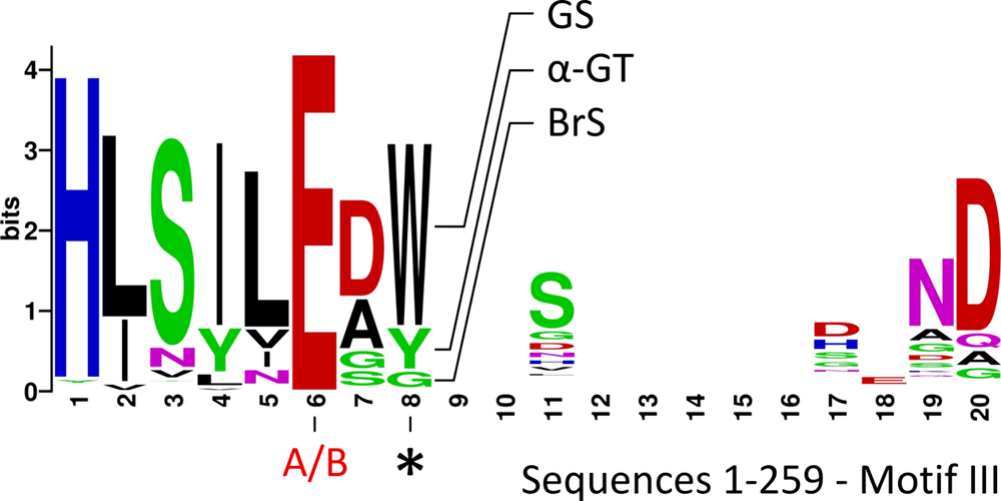
Sequence logo of conserved sequence motif III of all 259 sequences; the fully conserved catalytic acid base (A/B) and the residue 2 positions downstream of it (*) are indicated; the latter discriminates between GS, BrS and α-GT enzymes with a conserved tryptophan, glycine or tyrosine residue at this position, respectively. The gaps occur due to a few sequences with a longer motif III.

In addition to loop A2, the GH70 conserved motif III is another region that splits the GH70 set into different groups, seemingly correlating with reaction specificity. Especially the residue two positions downstream of the catalytic acid/base glutamate (Figure 5) is of importance. Crystal structures have shown that in glucansucrases this residue, a tryptophan, contributes a specific hydrogen bond to sucrose, facilitating its utilization as donor substrate to initiate the polymerization reaction; in addition, the aromatic ring structure of tryptophan contributes to acceptor substrate binding via stacking interactions.^12,13^ In contrast, in branching sucrases the large aromatic side chain is absent, and replaced by a small nonaromatic residue. Within the group of 208 GH70 sequences with a long loop A2, 185 sequences feature a tryptophan in motif III; all characterized enzymes within this subset are glucansucrases. In contrast, in the remaining 23 sequences the tryptophan is replaced by glycine or another small nonaromatic residue; here, the characterized enzymes were unable to catalyze α-glucan synthesis from sucrose alone, but required a dextran-like acceptor to perform the polymerization reaction. Thus, depending on the presence or absence of tryptophan in motif III, we assigned glucansucrase or branching sucrase specificity to 185 respectively 15 entries of the 208 enzymes with a long loop A2. The remaining 8 entries are the significantly longer sequences harboring two A/B/C/IV cores; e.g., the characterized DsrE from *Leuconostoc citreum* NRRL B-1299.^14,39^ The N-terminal core (CD1) always features a tryptophan in motif III, which is absent in the C-terminal core (CD2). This is consistent with studies showing that CD1 functions as a glucansucrase synthesizing a dextran-type α-glucan from sucrose, while the CD2 requires a dextran acceptor to catalyze a branching sucrase reaction.^35^

For the 51 predicted α-GT enzymes, featuring a short loop A2, the corresponding motif III residue usually is a tyrosine (Figure 5). The crystal structure of the GtfB from *L. reuteri* NCC 2613 in complex with acarbose (PDB: 7P39(23)) suggested that, similar to the tryptophan side chain in glucansucrases, the aromatic tyrosine side chain still can provide aromatic stacking with maltooligosaccharide acceptor substrates.

### 3.5 Combined Features Support the Classification of GH70 (Sub)Groups

**Figure 6:**
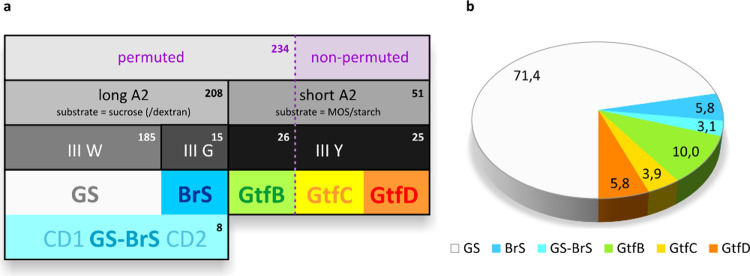
(a) Classification of 259 representative GH70 enzymes into subgroups, based on permutation of the catalytic core, the length of loop A2, and the motif III residue (W/G/Y) two positions upstream of the catalytic acid/base. GS = glucansucrase; BrS = branching sucrase; GS-BrS = twin glucansucrase-branching sucrase enzymes with two catalytic cores CD1 and CD2; GtfB/-C/-D = the three subgroups of α-glucanotransferases. The numbers represent the number of sequences found in each subgroup. (b) The relative occurrence (%) of each enzyme subgroup in the representative set of 259 sequences.

Combining the sequence and structural features of the GH70 A/B/C/IV cores mentioned above, the (sub)groups of enzymes within this glycoside hydrolase family as well as their relative occurrence in the representative set become evident (Figure 6a). Three levels of distinction can be observed: (1) the presence or absence of circular permutation; (2) a long or short loop A2; (3) the residue in motif III two positions downstream of the acid/base glutamate (tryptophan/glycine/tyrosine). Together, as earlier studies have shown, we distinguish the following subgroups: glucansucrases (GS), branching sucrases (BrS), GtfB-, GtfC- and GtfD-type α-GTs.^1,7,19,20,53^ Finally, a special subgroup contains the significantly longer enzymes harboring twin A/B/C/IV cores with GS- and BrS-specificity, respectively (Figure 6a). The relative occurrence of the GH70 subgroups shows that GS enzymes are by far the most abundant (71.4% of 259 representative enzymes), followed by GtfB-type α-GTs (10.0%), BrS and GtfD (both 5.8%), GtfC (3.9%) and GS-BrS (3.1%) (Figure 6b).

### 3.6 The GH70 A/B/C/IV Core

Currently, crystal structures have been published of 13 GH70 enzymes (7 GSs, 1 GS-BrS CD2, 4 GtfBs and 1 GtfC; corresponding representative PDB entries are listed in Table S1). Since all these structures were of constructs significantly truncated at the N- or C-terminus, we generated and analyzed 3D models of all 259 representative enzymes in our set. All enzymes comprise a structurally similar A/B/C/IV core, consisting of 820–870 residues in permuted enzymes and 670–700 residues in nonpermuted enzymes (partly due to a smaller single-segment domain IV). Thirteen sequences may be regarded as “minimal” GH70 sequences, as they do not feature other domains than those in the core.

### 3.7 Phylogeny of the GH70 Core

**Figure 7:**
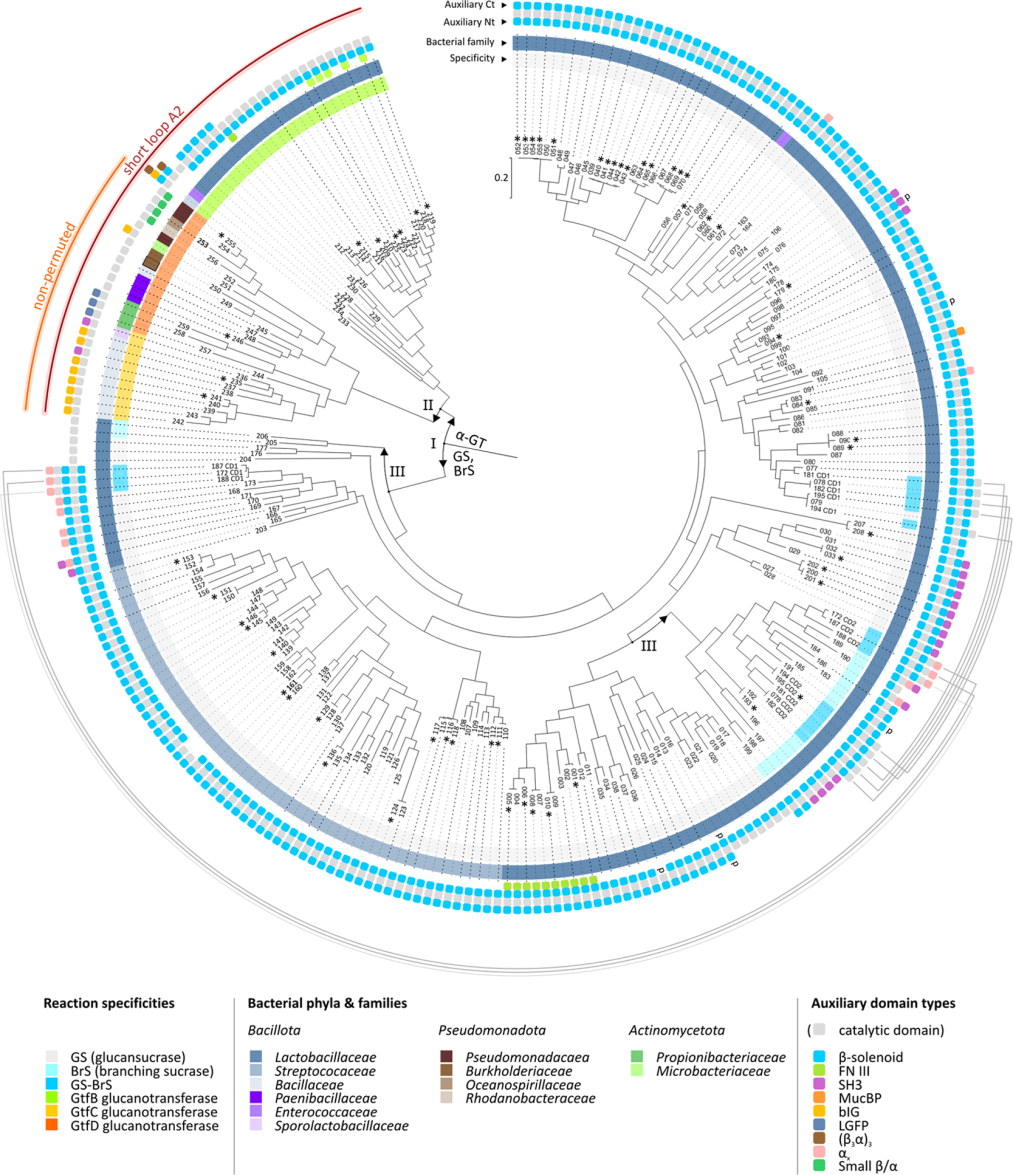
Phylogenetic tree (center) based on the alignment of 259 representative GH70 cores (domains A/B/C/IV, corrected for permutation); entry numbers correspond to those listed in Table S1. The highest log likelihood tree calculated with MEGA X (54) is shown. Initial tree(s) for the heuristic search were obtained automatically by applying Neighbor-Join and BioNJ algorithms to a matrix of pairwise distances estimated using a JTT model and then selecting the topology with superior log likelihood value. Branch lengths were measured in the number of substitutions per site. All positions with less than 95% site coverage were eliminated, i.e., fewer than 5% alignment gaps, missing data, and ambiguous bases were allowed at any position (partial deletion option). There was a total of 512 positions in the final data set. The bootstrap consensus tree was inferred from 500 bootstrap replicates Characterized enzymes are indicated by an asterisk (*). Bifurcation point I signifies the separating branches of GS/BrS/GS-BrS and α-GTs; point II shows where permuted and nonpermuted enzymes separated, and the two points III indicate the branching off of BrS catalytic cores. The inner ring is color-coded according to predicted enzyme specificity, clearly separating the GS/BrS/GS-BrS enzymes (no. 001–208) from the α-GT enzymes (no. 209–259). The middle ring is color-coded for the bacterial family in which the enzyme is found. The outer ring with rounded squares represents the predicted auxiliary domain type in each sequence (but not the number or length of these domains), with the inner ones corresponding to N-terminal and the outer ones to C-terminal domains; black lines (−) represent the catalytic core. Partial sequences are indicated by a “p” at the respective terminus. The gray circle segments on the lower half shows which CD1 and CD2 catalytic cores belong to the same twin GS-BrS enzyme. Enzymes with a short A2 loop (predicted α-GT enzymes), as well as enzymes with a nonpermuted catalytic core, are indicated with a red or orange circle segment, respectively.

Alignment of 267 A/B/C/IV core segments (after correcting for permutation) allowed calculation of a phylogenetic tree (Figure 7), which largely reflects the subgroup specificities described above. First and foremost, the GS- and BrS-type enzyme cores (no. 001–208), forming the largest group, are clearly separated from the α-GT cores (no. 209–259; GtfB, -C and -D), reflecting the different substrate specificities between them and correlating with the presence of either a long or short loop A2, respectively. Within the GS/BrSs clade, almost all of the 23 BrS enzyme cores (single BrS or GS-BrS CD2) are clustered together in one branch. The only exceptions are two branching enzymes from *Apilactobacillus* species (no. 205 and 206) which, given their position in the phylogenetic tree, seem to be rather unique; they are close to three GSs, either from another *Apilactobacillus* species or from *Nicoliella spurrieriana* and *Lactobacillus* Sy-1. Within the α-GT clade, permuted enzymes (GtfB) and nonpermuted enzymes (GtfC, GtfD) appear as distinct branches; the latter two also form separate sub-branches.

Regarding the 8 A/B/C/IV core pairs of the twin GS-BrS enzymes, two sets can be distinguished: for the 5 *Leuconostoc* enzymes (no. 078, 181, 182, 194, 195), the CD1 and CD2 cores are relatively close; in contrast, the CD1 cores of the 3 *Apilactobacillus kunkeei* enzymes (no. 172, 187, 188) are further apart from their CD2 partners (and from the CD2 cores of the *Leuconostoc* GS-BrSs).

### 3.8 Auxiliary Domains

Besides the A/B/C/IV core, the vast majority of the representative set of GH70 enzymes also features auxiliary domains at either or both termini. In the reported GH70 crystal structures, significant segments of these auxiliary domains were absent due to N- and/or C-terminal truncation. AlphaFold modeling allowed us to study these missing segments, although several of the 259 models featured segments with low pLDDT scores, especially at N-termini. Sometimes these segments only comprise a predicted signal sequence of about 30–40 residues (e.g., for most of the GtfCs and GtfDs); in other cases, they are relatively rich in small residues (e.g., Ala/Ser/Thr/Val) and can be up to 350 residues long.^32,54^

**Figure 8:**
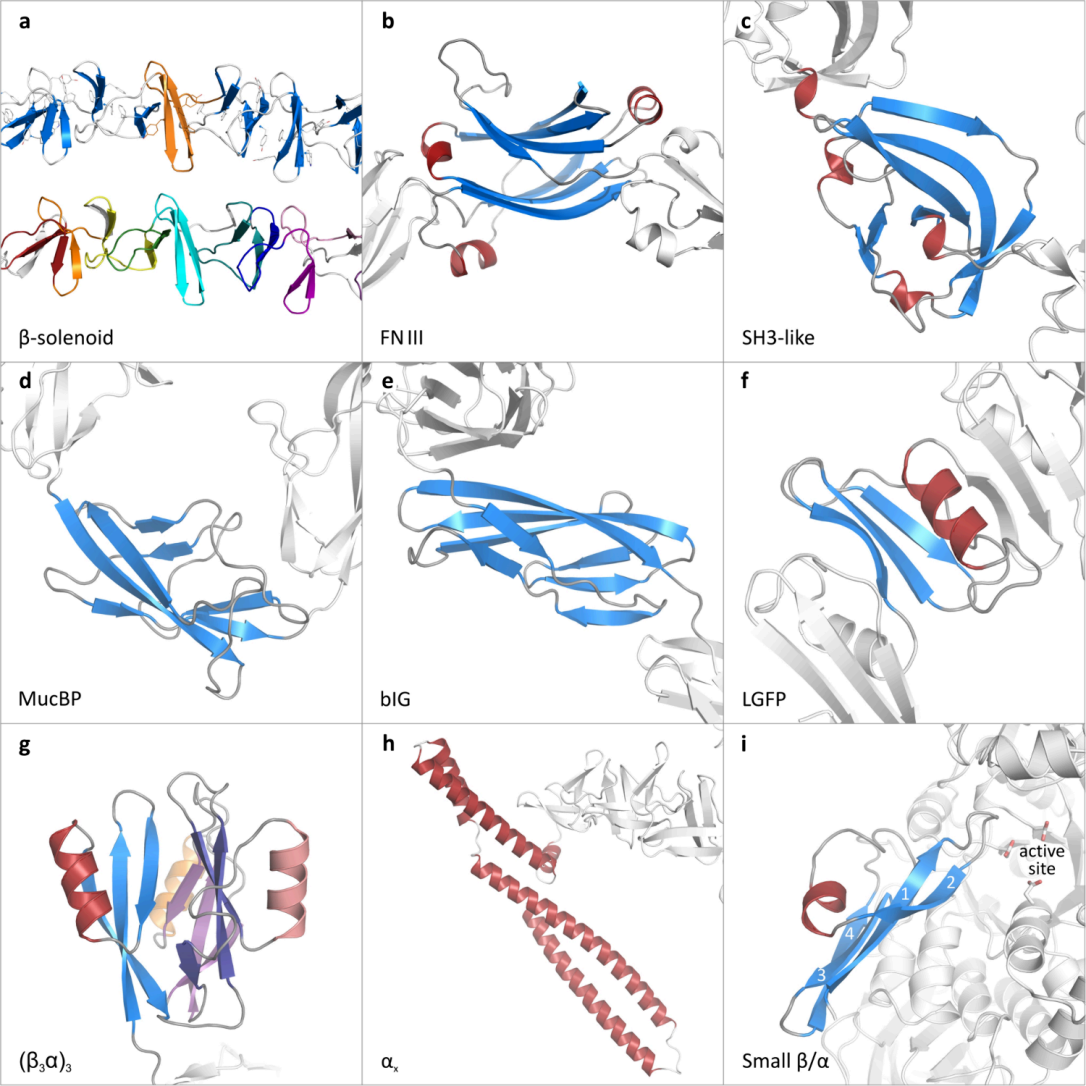
Examples of the (predicted) 3D structures of auxiliary domains in GH70 enzymes. Polypeptide segments with pLDDT scores <55 are not shown. (a) Part of the C-terminal β-solenoid domain of *Leuconostoc mesenteroides* NRRL B-512F glucansucrase (no. 044). The top picture is colored by secondary structure, with one module (the 2-stranded β-sheet connected by a β-hairpin followed by a loop) highlighted in orange. The bottom picture shows the same structure, with several YG-repeats colored differently. (b) Part of the N-terminal segment of *Limosilactobacillus reuteri* 180 Gtf180 glucansucrase (no. 001), containing five FNIII domains, with one FNIII domain highlighted. (c) One of the C-terminal SH3-type auxiliary domains of *Oenococcus oeni* glucansucrase (no. 027). (d) C-terminal segment of *Fructilactobacillus hinvesii* glucansucrase (no. 092) with its third MucBP domain highlighted. (e) C-terminal bIG domains in the GtfC-type α-GT from *Exiguobacterium* sp. H66 (no. 239); the first one is highlighted. (f) C-terminal LGFP domains in the GtfD-type α-GT from *Propioniferax innocua* (no. 257); the fourth one is highlighted. (g) The C-terminal (β_3_α)_3_ domain of *Lactococcus nasutitermitis* GtfB-type α-GT (no. 233); the first, second and third subdomain (β_3_α) are colored blue/red, purple/pink and violet/orange, respectively. (h) C-terminal α-helical bundle in the GS from *Apilactobacillus kunkeei* (no. 168). (i) The small β/α subdomain of the GtfD-type α-GT from *Oceanospirillaceae bacterium* (no. 253), adjacent to the active site of the enzyme.

**Figure 9:**
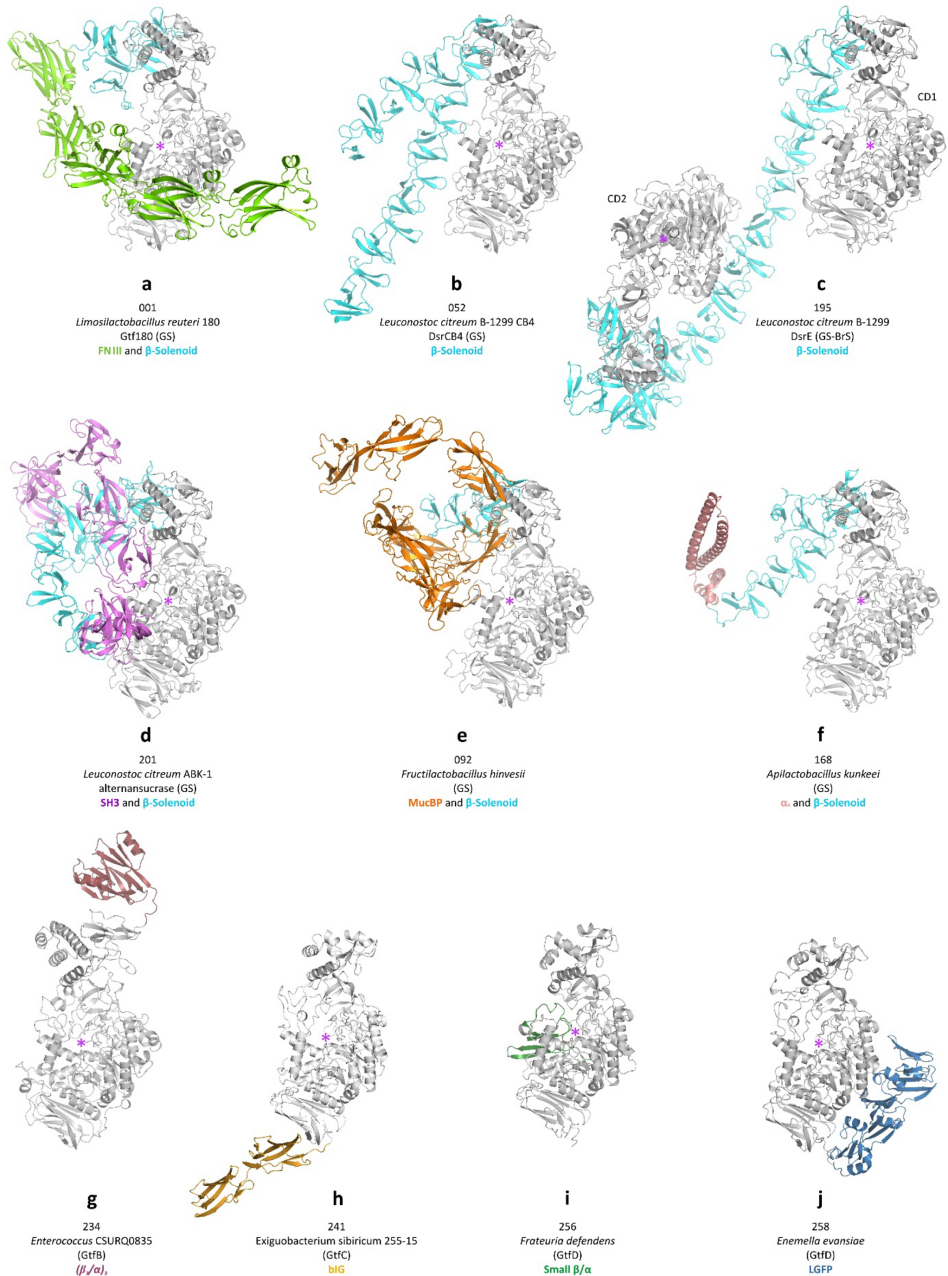
Representative examples of AlphaFold models of GH70 enzymes; segments with low pLDDT scores and without secondary structure are not shown. All models are in about the same orientation regarding the A/B/C/IV core domains (gray); auxiliary domains are colored differently for each domain type. For the twin GS-BrS (panel c), the N- and C-terminal catalytic entities are indicated as CD1 and CD2, respectively. The approximate location of the active site(s) is indicated by a purple star. The numbers correspond to those listed in Table S1. (a) Glucansucrase Gtf180 from *Limosilactobacillus reuteri* 180 (LrGtf180). (b) Dextransucrase DsrCB4 from *Leuconostoc citreum* B-1299 CB4. (c) Bifunctional dextransucrase/branching sucrase DsrE from *Leuconostoc citreum* B-1299. (d) Alternansucrase from *Leuconostoc citreum* ABK-1. (e) Putative glucansucrase from *Fructilactobacillus hinvesii*. (f) Putative glucansucrase from *Apilactobacillus kunkeei*. (g) Putative GtfB-type α-glucanotransferase from *Enterococcus* CSURQ0835. (h) GtfC-type α-glucanotransferase from *Exiguobacterium* 255–15. (i) Putative GtfD-type α-glucanotransferase from *Frateuria defendens*. (j) Putative GtfD-type α-glucanotransferase from *Enemella evansiae*.

Investigating the predicted domain organization for each of the 259 GH70 sequences revealed an impressive structural diversity. We identified 9 different auxiliary domain topologies (Figure 8), appearing either N- or C-terminal (or both) to the A/B/C/IV core; notably, several GH70 enzymes contain two or even three different auxiliary domain types. The domain organization of all 259 entries is schematically depicted in Figure S2, while examples of whole 3D models are shown in Figure 9. Below, we describe each of the observed auxiliary domain type.

#### 3.8.1 β-Solenoid

β-Solenoid domains (Figure 8a) are by far the most abundant auxiliary domain type in the representative GH70 set; they were found in 228 of the 259 entries (87.7%), in almost all enzymes with a circular permutation (GS, BrS, GtfB-type α-GTs) but not in nonpermuted ones (GtfC-/GtfD-type α-GTs) (Figure S2). The β-solenoid topology consists of repeating structural units of 20–24 amino acids, each forming a 2-stranded antiparallel β-sheet (connected by a β-turn) followed by a short loop lacking secondary structure; these units (hereafter named “β2”) fold into a superhelical structure. In some cases, the very C-terminal part of a β-solenoid domain forms a 3-stranded antiparallel β-sheet. Often present at both termini of GH70 enzymes, in many cases they connect the A/B/C/IV core to other types of auxiliary domains (Figure 9a/d/e/f) and vary greatly in length (Figure S2). In the first reported crystal structure of a (truncated) GH70 enzyme, *L. reuteri* 180 Gtf180^12^), the N- and C-terminal segments constituting domain V contained β-solenoid repeats. The longest β-solenoid domains (up to ≈830 residues, ≈38 repeating β2 units) are found in the GS-BrS enzymes, where they constitute the so-called glucan-binding domain (GBD) connecting the two catalytic cores (Figure 9c). Interestingly, they do not appear C-terminal of the BrS catalytic core. When β-solenoid domains are present at both the N- and the C-terminus, the segments nearest to the A/B/C/IV core lie close together and in fact interact with each other (e.g., Figure 9b). Finally, in many cases, β-solenoid segments preceding the A/B/C/IV core are rather short (≈ 45 residues) and comprise just two β2 units, especially in sucrase-type enzymes.

Given the enormous variation in length, sequence alignment of β-solenoid domains is very challenging. However, when we divided them in an N- and a C-terminal set (201 and 186 segments, respectively), it appeared that in the better aligning segments, in particular aromatic residues (mostly tyrosine) and glycine showed the highest conservation. Sequence repeats were identified that have been described in earlier studies.^29−32^ For example, the putative cell-wall/choline binding repeat (InterPro IPR018337) consists of ≈21 residues and corresponds to a single β2 unit. The ≈63-residue glucan-binding repeat (IPR027636) partly overlaps with it, contains two copies of a consensus 33-residue segment (“A-repeat”) identified in glucan synthesizing or glucan binding enzymes from *Streptococcus* and *Leuconostoc*(31,55) and structurally corresponds to three β2-units. Likewise, we identified B-, C- and D-repeats in β-solenoid domains, as well as their common YG-repeat. Thus, despite the presence of a common structural motif, the β-solenoid domains contain different types of sequence repeats; a preliminary analysis of the RADAR results showed that repeats tend to be more conserved within each of the bacterial genera.

#### 3.8.2 FNIII

In 16 permuted GS or GtfB enzymes from *Lactobacillus* spp., auxiliary domains with a common β-sandwich topology were identified (Figures 8b and 9a); they always appear N-terminal to the A/B/C/IV and are linked to the core via a short β-solenoid segment (Figure S2). Interestingly, these domains, each consisting of ≈125 residues, always are present with 5 copies. A PDBeFold search gave the highest scores with Fibronectin type III (FNIII) proteins or domains (InterPro IPR003961); yet, in none of the 16 entries this fold was identified in UniProt or GenBank. The FNIII topology resembles an immunoglobulin-like fold, with two sandwiched β-sheets formed by strands A-B-E and C–D-F-G. In some cases, one or two short extra β-strands or a short extra α-helix were found in the predicted structure. Sequence alignment (Figure S3a) revealed high sequence identity between the 16 full FNIII segments (5 domains, 55.4–92.3%); for the 16 × 5 individual domains it is lower (25.6–82.0%). Similarly, all 16 × 5 individual FNIII domains superimpose well, but all 16 first (N-terminal) domains resemble each other more closely than they resemble the other four domains in the same entry, and this is true for each of the 5 copies. GH70 enzymes lack the RGD motif found in the corresponding loops of many FNIII containing proteins.^56,57^ However, all 16 × 5 FNIII modules contain the RDV repeat first described for a few glucansucrases from *Lactobacillus* species.^32^ From our alignment (Figure S3a), we can derive a slightly modified RDV consensus sequence: **R**(P/N/S/T/Q)**DV**-x_11–12_-S/A**G**Y/F-x_17–22_-**R**(Y/F)S (changes underlined) with the residues in bold being fully conserved in all 16 entries. In addition, about 64 and 50 residues upstream of the RDV motif, there is a fully conserved D and a virtually conserved GW pair, respectively. Structurally, the R/D/V/G and D/GW cluster together on the surface of the FNIII module, with the arginine and two aspartate residues forming salt bridge interactions (Figure S3b). Finally, several of the aromatic residues in the FNIII domains are highly conserved, but only some of them are accessible at the surface (indicated in Figure S3a).

#### 3.8.3 SH3-like

In 23 enzymes (permuted or nonpermuted), the predicted structure features 1–7 copies of an auxiliary domain that bears resemblance to the SH3 (src Homology-3) topology (InterPro IPR003646). The SH3 superfamily contains different subfamilies, all based on a ≈60-residue domain with a highly twisted and open β-sheet forming an open barrel conformation. The SH3-like domains always occur C-terminal to the GH70 core domains (Figure 9d; Figure S2). In the UniProt or NCBI Protein Database entry of the 23 sequences, some of these SH3-like domains were already detected, namely pfam13457 (SH3_8), pfam08239 (SH3_3), and pfam19087 (DUF5776). Most of the predicted domains contain approximately 75–90 residues, with some variation in the topology regarding the connecting loops, but structurally similar to the SH3-like domains of the invasion protein InlB from *Listeria monocytogenes* (PDB: 1M9S(58)), despite very low sequence identity (5.6–24.6%).

Sequence alignment of the SH3-like domains revealed two groups (Figure S4a,b). The first group, containing 17 of the 23 GH70 entries could be aligned with the so-called GW domain, a divergent SH3 subfamily (SH3_8) also known as cell wall targeting (CWT) signal (pfam13457; InterPro IPR038200). GW domains feature a conserved and buried glycine-tryptophan dipeptide located in the last β-strand. The GH70 SH3_8 domains share low sequence identity (generally 25–45%), but do contain some homologous regions. First, regarding the GW motif, only the tryptophan is virtually conserved while the glycine is mostly replaced by an aliphatic or aromatic residue, e.g., leucine, valine, threonine or tyrosine (Figure S4a). Second, the longest region with higher homology resides in a β-strand and following loop, and corresponds to the APY motif first identified in the alternansucrase ASR from *L. mesenteroides* NRRL B-1355;^17,55^ no. 202 in our set). The APY motif is present in 17 of the 23 SH3-containing sequences. Interestingly, the GW-like and APY motifs structurally lie adjacent to each other, with the tryptophan (GW-like motif) and penultimate proline (APY motif) oriented perpendicular to the surface, their rings mutually stacking (Figure S4b).

For the second group, containing the remaining GH70 entries with detected SH3_3 or DUF5576 domains, alignments are less consistent (Figure S4c). Compared to the first group, they lack the APY motif and its conserved proline, while the aromatic residue of the GW motif observed in SH3_8 type domains is mostly replaced by a tyrosine.

#### 3.8.4 MucBP

The (predicted) glucansucrase from *Fructilactobacillus hinvesii* (no. 092) contains C-terminal domains resembling the MucBP domain topology (InterPro IPR009459) and is the only enzyme in our set predicted to contain this domain (Figures 8d and 9e). In the NCBI Protein Database entry (WP_252797321.1) of this enzyme, one copy of a MucBP domain was predicted; however, our predicted structure contains eight of them, each of them aligning very well and showing moderate to high conservation (46.3–88.1%) (Figure S5a). The two β-sheets (4- and 2-stranded) of each domain form an elongated immunoglobulin-like fold of approximately 83 residues (Figure S5b), although in our predicted structure not all β-strands are detected. Among the highest conserved residues are several aromatic residues; most of these are buried in the core of the domain while a few lie at the surface.

#### 3.8.5 bIG

Ten of the α-GT enzyme models in our set contain an ≈85-residue elongated domain resembling a bacterial immunoglobulin (bIG)-like topology, C-terminal to the GH70 core domains (Figure 8e; Figure 9h). Almost all of these were already predicted from the sequences and in earlier published AlphaFold models of GtfC-like α-GTs.^26^ First, in 8 of the 10 enzymes, two copies of a type 2 bIG domain (bIG_2; IPR003343; pfam02368) are predicted, displaying a two-layered sandwich of β-sheets in a Greek key motif. Alignment of all bIG_2 domains (Figure S6a) shows a moderate conservation of aromatic residues (Figure S6b) and some exposed on the domain surface. Second, two entries contain a single bIG_3 (pfam07523) domain: no. 233 (WP_213533855.1 from *Lactococcus nasutitermitis*, the only permuted enzyme and putative GtfB in this subset), and no. 251 (NIJ 05635.1 from *Frigoribacterium faeni*.

#### 3.8.6 LGFP

Entries no. 257–259 of our representative GH70 set contain 5 C-terminal copies of a ≈54-residue domain consisting of an N-terminal α-helix and a 3- or 4-stranded antiparallel β-sheet (Figures 8f and 9j). In the sequences of these enzymes, the LGFP-repeat (InterPro IPR013207; pfam 08310) was identified in the corresponding regions, but not always (in no. 259 they were not identified at all). The only reported experimental structure containing an LGFP domain is the mycoloyltransferase MytA from *Corynebacterium glutamicum*.^59^ The conserved Leu-Gly-Phe-Pro motif in each of its 5 LGFP domains is located on alternating sides of a stalk-like extension of the transferase domain, but, despite the structural similarity, only the glycine is fully conserved in the GH70 enzymes (Figure S7a), while the other three residues in the motif vary, but retain the physicochemical nature of the side chain. In MytA, several semiconserved aromatic residues in the LGFP domains were observed to be involved in acetate binding; the corresponding positions in the GH70 GtfD enzymes are also semiconserved (Figure S7b). Finally, all LGFP domains in the GH70 enzymes have two fully conserved cysteine residues which are predicted to form a disulfide bridge, but are absent in those of MytA.

#### 3.8.7 (β_3_α)_3_

In two predicted GtfB-type α-GT enzymes of our representative GH70 set (no. 233 and 234), a compact C-terminal domain is predicted consisting of 3 subdomains arranged by ≈60° rotation around a central axis (Figure 8g). These two entries are the only GtfBs in our set that are from non-*Lactobacillus* species, namely *Lactococcus* and *Enterococcus*. The three ≈45-residue subdomains appear C-terminal to a bIG domain or immediately after domain IV of the core, respectively (Figure 9g) and align well with moderate sequence identity (38.6–54.6%; Figure S8a). They share a very similar fold consisting of a 3-stranded antiparallel β-sheet and an α-helix in the order β1-β2-α–β3; hence we designated this auxiliary domain as (β_3_α)_3_. The α-helices sit on the outside of the domain (Figure S8b). Notably, these subdomains were detected as pfam18885 (DUF5648) in the sequences of the two enzymes, but until now no 3D structures had been described. Many of the aromatic residues are located near the surface, especially in grooves that form between the subdomains (Figure S8b) and are conserved between the two enzymes. Five or six other aromatic residues pack together in the center of the (β_3_α)_3_ domain, forming a hydrophobic core.

#### 3.8.8 α-Helices

Twelve AlphaFold models in our set have isolated α-helices, mostly appearing at the C-terminus and frequently arranged in a long 2- or 3-helix bundle (Figures 8h and 9f); they occur only in permuted GS and GS-BrS enzymes of Lactobacillaceae spp. In several cases these α-helices have significantly lower pLDDT scores than the rest of the protein. All predicted α-helices are rich in alanine, lysine and/or leucine residues, and to a lesser extent, glutamate and serine. For example, in the α-helical segment of glucansucrase Gtf-33 from *Lentilactobacillus parabuchneri* 33 (no. 084), 7 repeats were detected that correspond to two 49-residue KYQ repeats described by Kralj et al.^32^ RADAR analysis detected internal sequence repeats in several of the α-helical segments, although in general with low scores, and with no common motif detected between the enzymes.

#### 3.8.9 Small β/α

In a few GtfD-type α-GT models (no. 253–256), extra secondary structure elements are observed, sharing sequence similarity and forming a small subdomain of ≈50 residues (Figures 8i and 9i). The GtfD enzymes with this β/α subdomain are all from the *Pseudomonadota* phylum. A FoldSeek search with the β/α subdomain of no. 253 (residues 32–83) did not yield any characterized structural homologues. Unlike all other auxiliary domain types of nonpermuted GH70 α-GTs, the small β/α subdomains occur N-terminal to domain A, immediately after the predicted signal peptide. For example, no. 253 and 256 feature a 4-stranded antiparallel β-sheet with a short α-helix on one side. In no. 254 and 255, a similarly positioned sheet is also present and, while the sequence alignment shows similarity (Figure S9a), only the last two β-strands are predicted reliably by AlphaFold. The small β/α subdomain packs against the side of the catalytic domain A, close to helices α6 and α7 of the (β/α)_8_-barrel in domain A, and seem to lie close to or even partially block the acceptor side of the binding groove of α-GT enzymes (Figure S9b). The second β-strand features a conserved tryptophan residue (e.g., W47 in no. 253); its aromatic side chain packs against the side of domain A and does not seem solvent-accessible.

#### 3.8.10 None

There are 5 sucrase sequences (3 GS and 2 BrS) that lack auxiliary domains and thus only consist of the A/B/C/IV core preceded by a signal peptide, although in one case (no. 206) the preceding N-terminal part is significantly longer (210 residues) but could not be reliably modeled by AlphaFold. Notably, these 5 sucrases cluster together in the phylogenetic tree (Figure 6), and three of them are from *Apilactobacillus* spp. In addition to the sucrases, 8 GtfD-type α-GTs also lack auxiliary domains.

### 3.9 Distribution of Specificity, Origin and Auxiliary Domain Topology

We investigated if correlations could be detected regarding the distribution of three “characteristics” of the 259 enzymes: reaction specificity, bacterial origin (phylum/family), and auxiliary domain type. The left half of Table 1 lists the occurrence of (predicted) reaction specificity when grouped by bacterial phylum and family, and is graphically represented in Figure S10a. Note that the Lactobacillaceae family is by far the most represented one in our set. The data reveal that most enzyme specificities are concentrated in one or two bacterial families; on the other hand, the GtfD-type α-GTs are distributed over 8 or the 12 bacterial families and are the only specificity found in Gram-negative bacteria belonging to the Pseudomonadota phylum, as well as the only reaction specificity found in the Actinomycetota phylum. Furthermore, Lactobacillaceae display the largest variation regarding specificity, with 4 specificities (GS, BrS, GS-BrS and GtfB). Finally, there is a clear distinction between permuted and nonpermuted enzymes: permuted enzymes exclusively appear in Lactobacillaceae, Streptococcaceae and Enterococcaceae (all belonging to the Bacillota phylum), while nonpermuted enzymes (names and numbers in italics in Table 1) exclusively appear in the other 9 families.

**Table 1:** Occurrence of Predicted Reaction Specificity and Auxiliary Domain Types in a Representative Set of 259 GH70 Enzymes, Grouped By Bacterial Phyla and Families^a^

						auxiliary domain topology occurrence									
phylum	family	Gr	LAB	no. of entries	specificity	β-sol	FNIII	SH3	Muc	bIG	LGFP	(β_3_α)_3_	α_*x*_	β/α	none
Bacillota	Lactobacillaceae	+	yes	128	GS	125	10	15	1				9		3
				15	BrS	13		6							2
				8	GS-BrS	8							3		
				24	GtfB	23	6								
	Streptococcaceae	+	yes	56	GS	56									
				1	GtfB	1									
	Enterococcaceae	+	yes	1	GS	1									
				1	GtfB	1						1			
	Bacillaceae	+	no	9	*GtfC*			*1*		*8*					
				1	*GtfD*										*1*
	Paenibacillaceae	+	no	3	*GtfD*										*3*
	Sporolactobacillaceae	+	yes	1	*GtfC*			*1*							
Pseudomonadota	Pseudomonadaceae	–	no	3	*GtfD*									*2*	*1*
	Burkholderiaceae	–	no	2	*GtfD*										*2*
	Oceanospirillaceae	–	no	1	*GtfD*									*1*	
	Rhodanobacteraceae	–	no	1	*GtfD*									*1*	*1*
Actinomycetota	Propionibacteriaceae	+	no	3	*GtfD*						*3*				
	Microbacteriaceae	+	no	1	*GtfD*					*1*					
totals				185	GS	182	10	15	1				9		3
				15	BrS	13		6							2
				8	GS-BrS	8							3		
				26	GtfB	25	6			1		2			
				10	*GtfC*			*2*		*8*					
				15	*GtfD*					*1*	*3*			*4*	*8*

a Gr = Gram classification; LAB = lactic acid bacteria; β-sol = β-solenoid; FNIII = FNIII-like; SH3 = SRC Homology 3; Muc = MucBP (mucin-binding protein) domain; bIG = bacterial immunoglobulin-like domain group 2; LGFP = Leu-Gly-Phe-Pro domain; α_*x*_ = α-helices; Other = small subdomain close to domain A. Importantly, the number given for each auxiliary domain type does not represent the number of observed domains in each sequence, but rather the number of sequences in which the domain type was predicted. The “None” column lists cases where no auxiliary domains were found or could not be reliably. The “Totals” row lists the sums of all phyla/families. Names and numbers in italics represent the non-permuted enzymes (GtfC, GtfD)

The right half of Table 1 shows the occurrence of the different auxiliary domain types; a graphical representation of their distribution over GH70 reaction specificities is shown in Figure S10b. When comparing permuted and nonpermuted enzymes, permuted ones show a higher diversity (7 different domain types) than nonpermuted ones (4 types). Furthermore, β-solenoid, FNIII, MucBP, (β_3_α)_3_ and α_*x*_ type domains are exclusive to permuted enzymes, LGFP and small β/α are exclusive to nonpermuted ones, and SH3 and bIG domains are found in both. When comparing sucrases with α-GTs, the latter show more diversity with 7 different domain types; MucBP and α_*x*_ are only found in sucrases, while bIG, LGFP, (β_3_α)_3_ and small β/α are exclusive to α-GTs. The MucBP-type auxiliary domain in *Fructilactobacillus hinvesii* is not observed in the five other GH70 enzymes of this *Fructilactobacillus* genus. Thus, overall, there is some correlation regarding auxiliary domain type, but the distribution is not mutually fully exclusive if we consider (non)permutation or reaction specificity.

We also investigated if the auxiliary domain types correlate with bacterial phyla and families, shown in Figure S10c. Here, Lactobacillaceae spp. show the highest diversity (5 different auxiliary domain types). Furthermore, there is a fairly clear correlation between auxiliary domain type and bacterial phylum: except for bIG, all auxiliary domain types are exclusive to one of the three phyla, or even exclusive to a single bacterial family (FNIII, MucBP, LGFP). Finally, enzymes without any auxiliary domain occur in all three bacterial phyla.

## 4 Discussion

In this paper, we aimed to obtain a systematic overview of α-glucan synthesizing enzymes belonging to GH70. Over the last decades a picture began to emerge of large variation with regard to sequence length, domain organization, permutation and structural details. The discovery of new reaction specificities and subfamilies in recent years contributed to this view. While several substrate- and product specificities have been investigated and reviewed,^1,7,8,20,60^ (see also Table S2), a detailed structural overview of full-length enzymes so far has been limited because of the use of severely truncated enzyme constructs for crystallization, usually covering the core domains and only small parts of other domains. Our selection strategy (sequence similarity search, core domain alignment, redundancy filter, structural predictions) (Figure 2) resulted in a representative set of 259 GH70 sequences, facilitating systematic analysis and classification, as well as their phylogenetic relations. Regarding our strategy and subsequent analysis, a few notes have to be made. First, regarding the predicted auxiliary domains, the set of 259 enzymes may not be entirely representative, since (1) our selection was based on alignment of the A/B/C/IV core domains; (2) addition of characterized enzymes reintroduced some extra redundancy. Second, we found that in our set, the Lactobacillaceae family as well as the β-solenoid auxiliary domain type are by far the most abundant; this may skew the interpretation of the distribution of specificities, auxiliary domain types and bacterial origins. Nevertheless, we believe our strategy allowed proper processing of the enormous diversity within GH70. It is worth mentioning that more GH70 enzymes have been characterized than currently listed on the CAZy GH70 page; however, the 58 characterized enzymes in our set are well distributed (Figures 3 and 7), allowing us to predict reaction specificities of noncharacterized GH70 enzymes. Moreover, the predicted 3D structures presented here enables readers to link to earlier studies describing enzymes that so far had only been characterized biochemically.

A BLASTp search detected thousands of GH70 sequences, which, by applying our strategy (Figure 2) were systematically analyzed. The 95% redundancy filter on alignment of the core domains (A/B/C/IV) reduced the set by a factor of ≈10 (2673 to 259), indicating that many GH70 enzymes share very similar core sequences; the fact that the vast majority (234) is found in Lactobacillaceae and Streptococcaceae (Table 1; Table S1) may contribute to this high redundancy. Still, our set contains GH70 enzymes of at least 12 different bacterial families from 3 different phyla, including some that have not been reported before (e.g., Propionibacteriaceae), and from very different hosts and habitats (host digestive tract, soil, fermented food, marine environment, flower, human skin). All GH70 enzymes have a bacterial origin; GS, BrS and GtfB enzymes are only found in LAB, but the other α-GT enzymes clearly occur more widespread. GH70 enzymes are likely to play crucial roles in host survival, e.g., with their sucrose- or starch/maltooligosaccharide derived products at the basis of biofilm formation, offering protection against environmental extremes, or in symbiotic relationships with plants or animals. Detailed knowledge of the biochemical and structural properties of GH70 enzymes, and structure/function relationships of their products, appears highly relevant for their application,^1,10,11^ or for inhibition of these enzymes, e.g., to prevent dental plaque formation.^12,61^

Circular permutation of the A/B/C/IV is a widespread phenomenon in GH70:90.3% (234 out of 259) of the enzymes in the representative set is permuted, and the relative amount is even higher in the nonreduced set (95.7%; 2259 out of 2673). Apparently, permutation is a successful “strategy” for the bacterial families (Lactobacillaceae, Streptococcaceae and Enterococcaceae) in which it is found.

The CAZy classification system is based on sufficient sequence similarity;^3,4^ the GH70 family is characterized by the presence of seven conserved sequence motifs I–VII in the core domains.^28^ Previous phylogenetic analyses revealed that starch-converting GH70 enzymes with α-GT specificity form a separate branch.^8,22^ Notably, however, outside the GH70 motifs, sequence alignment and AlphaFold modeling clearly showed that also the length of loop A2 near the active site distinguishes α-GTs from the GS/BrS subgroup (Figure 3; Figure S1): the short 11-residue loop A2 in α-GT enzymes results in multiple donor subsites required for the starch/maltooligosaccharide substrate preference of α-GTs (Figure 3 – 3D loop A2). Remarkably, none of the sequences contains a loop A2 of intermediate length; the length and 3D structure of loop A2 thus provides a reliable criterion to predict α-GT or GS/BrS specificity. Within the GS/BrS group, a more subtle yet also reliable feature to define GH70 enzyme reaction specificity is the amino acid residue in motif III two positions downstream of the catalytic glutamate: either a tryptophan (GS-type specificity from sucrose) or a small nonaromatic residue (BrS-type specificity involving dextran as acceptor- and sucrose as donor substrate). In α-GTs, the almost completely conserved tyrosine at the corresponding position maintains the aromatic nature. The crystal structure of the GtfB from *L. reuteri* NCC 2613 in complex with acarbose (PDB: 7P39(23)) suggested that this tyrosine still provides aromatic stacking with maltooligosaccharide acceptor substrates. At the same time, due to the absence of a ring nitrogen, it cannot provide the hydrogen bond interaction to sucrose that was observed in the LrGtf180–sucrose complex.^12^ In this light it is also interesting that 3 of the 26 GtfB-type α-GTs do feature a tryptophan at this position.

The phylogenetic tree constructed here (Figure 7) offers additional insights in the functional evolution of GH70 enzymes. While previous phylogenetic analyses usually focused on a specific GH70 subgroup, bacterial origin or a small set of enzymes, our approach (1) encompasses a much larger set of enzymes, representative for the whole GH70 family, (2) is based solely on the common A/B/C/IV domains, and (3) involves a separate treatment of the two catalytic cores of GS-BrS enzymes prior to alignment. First and foremost, the phylogenetic distribution of the 259 sequences largely correlates with the different reaction specificities: GtfB, GtfC and GtfD each form distinct subclades separate from the GS/BrS clade, while almost all BrS cores also group in a distinct branch among GSs. This indicates that the different GH70 reaction specificities are the result of divergent evolution events, visible as bifurcation points in the phylogenetic tree. First, the separation of GH70 sucrases from α-glucanotransferases (bifurcation point I in Figure 7) correlates with the shorter loop A2 of the latter. The fact that the α-GT branch contains both permuted and nonpermuted sequences would suggest that this sucrase/α-GT separation occurred *before* the advent of circular permutation (bifurcation point II). This is in contrast with the findings of an earlier phylogenetic study,^26^ although it has to be noted that (nonpermuted) GH13 α-amylase sequences were included in those alignments. Second, the permuted enzymes exclusively originate from only three of the six bacterial families in the *Bacillota* phylum (Table 1; Figure S10a). Since it was proposed earlier that permutation involved gene duplication,^12^ the observed separation may indicate that such evolutionary events are restricted to certain bacterial families that possessed the “tools” to facilitate these events. This suggests that the GH70 sucrase/α-glucanotransferase separation (bifurcation point II in Figure 7) is a case of divergent evolution. The fact that no intermediate loop A2 lengths are found supports this as well. Third, almost all BrS catalytic cores (either single or as CD2 in GS-BrS) appear in a separate branch, suggesting divergent evolution from GS catalytic cores. Overall, given the large variation in specificities, domain organization and (non)permutation, and origin of GH70 enzymes, it remains challenging to piece together the evolutionary events leading to all GH13, GH70 and GH77 enzyme (sub)families with the GH-H clan. In this light it is interesting that recently circular permutation was found to occur in a GH13 enzyme.^62^

Apart from the common core domains, the N- and/or C-terminal auxiliary domains of GH70 enzymes have been the subject of several studies, but remained somewhat enigmatic. Compared to GH13 enzymes, GH70 enzymes possess an extra domain IV that so far seems unique for this family. The function of the mainly α-helical domain IV is not clear, although it has been suggested to provide a hinge between domains A/B/C and the auxiliary domains^13,63^ enabling the latter to be flexible and take up different positions relative to the catalytic site. This may be important in light of the often enormous size (up to tens of MDa) of the α-glucan products with respect to that of the enzymes. Regarding the auxiliary domains linked to domain IV, glucansucrases from different bacterial species were found to have different types of sequence repeats in their N- and C-terminal parts,^31,32^ suggesting some structural repeats might exist in these segments. Notably, in the reported crystal GH70 structures that did include such segments, the only experimentally observed auxiliary domain type was the β-solenoid type auxiliary domain (named “domain V” or “glucan binding domain”, (GBD)).^12,14,16,17^ Other auxiliary domain types have been predicted, but mainly by sequence similarity with non-GH70 proteins. Besides, most of the studies focused on a limited number of GH70 glucansucrases, mainly from Lactobacillaceae and Streptococcaceae spp. Our AlphaFold-assisted survey extends the GH70 space to several other bacterial families, annotates 259 representative sequences with (almost) complete 3D information, revealing an unexpectedly large structural diversity. Surprisingly, 9 different domain topologies were found (Figure 8), often present as multiple copies. Moreover, some enzymes feature two or even three different auxiliary domain types (Figure 9).

With sizes of 140–200 kDa, glucansucrases are large enzymes. The significant length/size (compared to the catalytic core) and diversity of their auxiliary domains suggest that these play important functional roles. Such roles have indeed been experimentally studied, but only for β-solenoid type domains. For example, different experimental approaches involving the β-solenoid type GBD (or domain V) of dextran- and alternansucrases indicated a role in binding of (intermediate) products, affecting the processivity of α-glucan synthesis. For example, GBD constructs of *Streptococcus downei* GtfI and *Leuconostoc mesenteroides* B-1299 DsrB were shown to bind biotinylated dextran.^31^ In another study, constructs of different (β-solenoid) GBD segments containing YG-repeats of dextransucrase DsrS were shown to bind dextran.^64^ Later, binding sites for dextran-type oligosaccharides in β-solenoid GBDs were found in crystal structures of DsrE,^35^ DsrM,^16^ and ASR,^37^ by mutation/chimera studies in DsrOK,^36^ and also by the severe loss of α-glucan polymer synthesis upon removal of β-solenoid segments e.g. in DsrS,^33^ and Gtf180.^34^ In the crystal structures of DsrE and DsrM, the observed binding sites in the (truncated) C-terminal β-solenoid domains are relatively close to the catalytic site and involve semiconserved aromatic residues, especially tyrosine. Our finding that in the better aligning parts of β-solenoid domains, tyrosine and glycine residues seem to be the most conserved suggests that carbohydrate binding sites may be conserved in other β-solenoid domains as well. Notably, the β-solenoid auxiliary domain topology is the most repetitive and the most variable one regarding length, with up to ≈38 repeats of the β2 unit (e.g., DsrE, Figure 9c). Indeed, these structural repeats represent varying sequences, but with a common motif. This supports the hypothesis that repeating β-solenoid motifs increase binding (of α-glucans) while at the same time, by sequence variation, decrease the vulnerability to immune surveillance.^29^ Since β-solenoid domains are by far the most abundant auxiliary domain type, this seems to be an important strategy in GH70, at least in Lactobacillaceae, Streptococcaceae and Enterococcaceae, the three bacterial families in which β-solenoid domains are found in virtually all GS, BrS and GS-BrS (Table 1). Notably, we did not find β-solenoid domains in nonpermuted GH70 enzymes; this is consistent with the earlier proposed evolutionary pathways for GH70 enzymes based on the “permutation-per-duplication” model,^12,27^ where the GH70 A/B/C/IV core was inserted into a β-solenoid domain. The acquisition of β-solenoid domains in permuted sucrase-type enzymes may have enhanced their ability to bind intermediate α-glucan products, enabling them to synthesize larger end products.^8^

Another proposed role for the β-solenoid domains is that of binding to the bacterial cell wall. The ≈21-residue cell wall/choline binding repeat (InterPro 018337) is detected in many of the GH70 β-solenoid domains, showing structural homology with bacterial toxins that bind components of cell wall lipoteichoic acids (LTA).^65^ However, a study with DsrP, a glucansucrase from *L. mesenteroides* IBT-PQ showed that, although the C-terminal β-solenoid domain does bind to the *L. mesenteroides* cell wall, binding is likely mediated by other components than LTA choline moieties.^54^ Furthermore, cell wall binding of the DsrP β-solenoid domain did not prevent dextran binding, suggesting that binding sites for cell wall components do not overlap with dextran binding sites. It remains to be seen whether this applies to other GH70 enzymes as well.

The finding of FNIII-type domains in 10 GS and 6 GtfB enzymes (Figures 8b and 9a) was unexpected; a 5-fold sequence repeat had already been detected in the glucansucrase GtfA from *L. reuteri* 121,^32^ but not recognized as constituting this fold, likely due to low sequence similarity with known FNIII domains. Inter- and intradomain sequence alignments, as well as the fact that all 16 entries contain 5 N-terminal FNIII copies, suggests that these domains may have been acquired as a whole. Since FNIII domains are only predicted in permuted enzymes, this acquisition likely took place after permutation events. From an analysis of all classes of carbohydrate-acting enzymes, Valk et al.^66^ suggested that these domains most likely function as linkers between the catalytic domain and carbohydrate binding modules (CBM). However, in the glycoside hydrolases of GH70, no (known) CBMs were detected. A role as linker thus seems less likely, also because the preceding ≈75–125 N-terminal residues could not be reliably modeled and may not form a structurally ordered domain. An alternative role may however be deduced from a conservation analysis of the GH70 FNIII domains; their most conserved residues locate on the outer surface of the two β-sheets, with the RDV motif being part of a highly conserved patch including an Asp-Arg-Asp salt bridge (Figure S3b). The role of these conserved patches remains to be determined, but may be related to the fact that the domains are only found in GH70 enzymes from Lactobacillaceae. In general, FNIII-type domains are found in a wide variety of extracellular proteins where they facilitate cell–cell adhesion and signaling through interaction with cell surface receptor proteins belonging to the integrin family, often using an RGD motif in the loop connecting β-strands F and G.^56,57^ However, also FNIII domains without this motif interact with integrins; the GH70 FNIII domains seem to belong to this group and still may be involved in cell wall interactions, perhaps using the RDV motif and/or conserved aromatic residues on the surface.

The observation of SH3-like auxiliary domains in 23 GH70 enzymes (Figures 8c and 9d), most of them structurally resembling GW domains, also hints at a role in cell wall binding. In general, SH3 domains are thought to mediate protein–protein interactions,^67^ although they may not do this independently; the (relative) position and nature of the protein that they are tethered to also seems important.^68^ SH3 domain family GW domains (GH3_8) are divergent members of the large SH3 superfamily only found in Gram-positive bacteria; they are known to bind bacterial cell surface polyanions such as LTA and host cell heparan sulfate proteoglycans. In GH70, the SH3 domains are also only observed in Gram-positive species (mainly *Leuconostoc*) and may serve a similar role, since GH70 enzymes reside in the extracellular space. However, their low sequence similarity (including variation of the GW dipeptide) suggests that after they had been acquired, the SH3-like auxiliary domains evolved further, perhaps to be tailored for specific interactions with cell surface components, depending on the species. In this light, the finding of a conserved stacking interaction between the (modified) GW- and APY-motifs in the GH70 SH3_8 modules is interesting (Figure S4b), although its more or less (buried) orientation suggests a role in fold stabilization rather than a role in binding ligands originating from the cell wall. Nevertheless, deletion of SH3 domains in *L. citreum* ABK-1 alternansucrase affected its catalytic efficiency and the product viscosity,^69^ suggesting that these domains may play an alternative role, namely in binding (intermediate) enzyme products, like was proposed for β-solenoid auxiliary domains.

The single case of auxiliary MucBP domains in a glucansucrase from *Fructilactobacillus hinvesii* (Figures 8d and 9e) exemplifies another, specific cell-wall binding property. *Fructilactobacillus hinvesii* was isolated from slender honey myrtle flowers; since LAB host adaptation is widely observed in insects, the finding of this fructophilic FLAB may have been the result of bacterial exchange via insect visits (e.g., honey bees, wasps). MucBP domains, having an immunoglobulin-like topology, have been detected mainly in Lactobacillaceae, often in conjunction with LPXTG anchors and/or carbohydrate processing/binding proteins such as lectins and glycoside hydrolases (e.g., GH13, GH66). It has been shown that these domains facilitate adhesion of *Lactobacillus* spp. to mucins, the highly glycosylated proteins found on the cell surface of epithelial cells; this interaction is thought to play a critical role in the beneficial effects of *Lactobacillus* species in the intestinal environment.^70^ Aromatic residues found on the surface of the *F. hinvesii* MucBP domain (Figure S7b) could play a role in facilitating cell wall interactions. It is of note that none of the 5 other GH70 enzymes from *Fructilactobacillus* spp. in our set, isolated from flowers or from fermented sourdough, contained predicted MucBP domains, but β-solenoid auxiliary domains instead; the *F. hinvesii* glucansucrase may therefore represent a more unique case within FLAB GH70.

Also the bIG domains, belonging to the immunoglobulin superfamily (IgSF) (Figures 8e and 9f), may play a role in cell surface adhesion or carbohydrate binding. Like in many immunoglobulin-like domain containing proteins, the GH70 bIG domains almost exclusively occur in pairs; their alignment (Figure S6a) suggests that they likely were acquired as such. Their exact role remains to be investigated, but studies on the two characterized GtfC enzymes from *Exiguobacterium sibiricum* 255–15^27^ and *Geobacillus* 12AMOR1^51^ showed that the truncated enzymes lacking both bIG_2 domains were still active and capable of producing α-glucans. This suggests that the bIG_2 domains are not essential for the catalytic function of GH70 α-GT enzymes.

The C-terminal 5-fold LGFP auxiliary domains of the GtfD enzymes from three Propionibacteriaceae species (Figures 8f and 9j) reveal significant sequence and structural homology with the LGFP domains of *Corynebacterium glutamicum* MytA, a transferase proposed to be involved in maintaining cell wall stability/integrity.^59^ Mutation studies and pull-down assays showed that the MytA LGFP domains interact with *C. glutamicum* peptidoglycan-arabinogalactan cell wall components, possibly through multiple ligand binding sites observed in the crystal structure. Notably, the aromatic residues involved in these binding sites are largely conserved in the GtfD LGFP domains (Figure S7a); since *Corynebacterium* and Propionibacteriaceae belong to the same phylum of Actinomycetota, the LGFP domains of GtfD α-glucanotransferases may provide similar interactions with cell wall components.

An auxiliary domain type with novel topology, (β_3_α)_3_, was found in two GtfB-type α-GT enzymes from non-*Lactobacillus* species (Figure 8g; Figure 9g); conserved clusters of aromatic residues on the surface (Figure S8b) may be involved in binding carbohydrate ligands unique to *Lactococcus* and *Enterococcus* species, but this needs to be experimentally confirmed.

The prediction of C-terminal long α-helical bundles (“α_*x*_”) in at least 12 of the 259 AlphaFold models is interesting (Figures 8h and 9f); however, as stated by the AlphaFold Protein Structure Database FAQ (https://alphafold.com/faq), long isolated α-helices should be treated with caution. The observed lower pLDDT scores of these segments indeed contribute to this uncertainty. Therefore, it remains to be seen if these α-helices really constitute an auxiliary domain in GH70 enzymes, or that they adopt a different fold or even disordered segments. The fact that the prediction of these auxiliary helical structures correlates with a single bacterial family (Lactobacillaceae) may indicate a function of these segments related to the specific properties and/or environment of this family, although the nature of such function is currently unclear.

The N-terminal small β/α subdomain, predicted for a few GtfD-type α-GTs, is interesting due to its location (Figures 8i and 9; Figure S9b). Being very close to the acceptor side of the active site groove, it may affect the binding of intermediate products and thus contribute to product specificity. Supporting evidence for such a role comes from comparing two recently characterized GtfDs. GtfD from *Paenibacillus beijingensis* DSM 24997 (no. 246) lacks a β/α subdomain; this enzyme uses amylose/starch to synthesize low as well as high molecular mass branched α-glucans with alternating (α1 → 4) and (α1 → 6) linkages and long α-1,4 linked fragments.^53^ In contrast, the GtfD from *Azotobacter chroococcum* NCIMB 8003 (no. 255), features the β/α subdomain and only synthesizes high molecular mass α-glucans of similar structure but with shorter α-1,4 linked fragments.^21^ In the AlphaFold models, the *A. chroococcum* enzyme has a less accessible acceptor binding groove due to the extra β/α domain, a feature that may well affect the transfer reaction during α-glucan synthesis. The fact that a tryptophan in the second β-strand is fully conserved may suggest a functional role for this residue, although its more or less buried side chain does not seem favorable for carbohydrate (stacking) interactions. Determination of crystal structures of these (or similar) enzymes with bound substrates is needed to experimentally confirm the role of the β/α domains.

When superimposing the GH70 AlphaFold models on their A/B/C/IV core, it became apparent that the relative position of the auxiliary domain(s) varied considerably, also among the 5 AlphaFold models usually generated for the same sequence (not shown). Moreover, the overall shape of auxiliary domains was different (e.g., fully extended, curved, or kinked) even if the domain topology was similar (compare for example the β-solenoid domains in Figure 9b,d). In this light, it is important to note that AlphaFold models are static representations; in their *in vivo* environment the structures likely present various degrees of flexibility. Such flexibility has been suggested earlier on the basis of small-angle X-ray scattering (SAXS) experiments and different crystal forms of the glucansucrase Lr Gtf180.^63^ In most GH70 crystal structures, the (truncated) auxiliary β-solenoid domain folds toward the core domains, but since also crystal structures are static and may “suffer” from lattice packing effects, it remains to be determined how dynamic the overall 3D fold is, and to what extent this plays a functional role in GH70 enzymes.

Intriguingly, almost all GH70 enzymes feature sequence segments (mostly N-terminal) that could not be modeled reliably by AlphaFold (Figure S2). Therefore, it is currently impossible to conclude whether these segments, often rich in small amino acid residues and containing semiconserved repeats, form folded domains or not, and the role of these segments remains unclear. On the other hand, the predicted 3D structures can assist in determining sites for truncation in a more precise way than has been possible based on primary sequence alone. This may allow for more successful GH70 enzyme expression strategies, aimed at (a) studying the role of auxiliary domains in a particular enzyme, (b) optimizing production levels of enzymes, or (c) the production of “tailored” enzymes with desired product specificity. Such optimization strategies will advance food-, health-, or biomaterial-related research involving GH70 enzymes.

Together, our set of 259 GH70 enzymes provides a representative collection of the different reaction specificities, phylogenetic relations and structural features, and covers a larger number of bacterial families than seen previously. While the core domains A/B/C/IV of GH70 enzymes bear high sequence similarity and share a very similar overall structure, specific details near the active site (loop A2, motif III) can be used to classify the different substrate specificities for sucrose (GS), sucrose + dextran (BrS) or starch-like substrates (α-GT). Intriguingly, our study uncovers an unexpected large structural diversity of auxiliary domains, revealing nine different topologies. Homology with proteins containing similar domains suggests that, besides a role in binding (intermediate) α-glucan products, most of them may also be involved in host cell wall interactions. Moreover, our set reveals correlations between enzyme reaction specificity, auxiliary domain type and bacterial origin; investigating these correlations further may help elucidate the role of GH70 enzymes in the bacteria that express them.

## Data Availability

AlphaFold models of the GH70 enzymes used in the present study are available from the corresponding author on reasonable request.

## Supporting Information Available

Representative set of 259 GH70 enzymes used in this study (Table S1). Sequence logos of loop A2 residues in the first 208 (top) and last 51 (bottom) representative GH70 sequences (Figure S1). Schematic domain organization of 259 GH70 enzymes used in this study (Figure S2). FNIII-type auxiliary domains in GH70 (Figure S3). SH3-type auxiliary domains in GH70 (Figure S4). MucBP-type auxiliary domains in GH70 (Figure S5). bIG-type auxiliary domains in GH70 (Figure S6). LGFP-type auxiliary domains in GH70 (Figure S7). (β3α)3-type auxiliary domains in GH70 (Figure S8). Small β/α-type auxiliary domains in GH70 (Figure S9). Occurrence/distribution of bacterial origin, enzyme specificity and auxiliary domain type (Figure S10) Chronological overview of review papers dealing with GH70 enzymes and their products (Table S2) (PDF)

## References

[ref1] Leemhuis H.; Pijning T.; Dobruchowska J. M.; van Leeuwen S. S.; Kralj S.; Dijkstra B. W.; Dijkhuizen L. Glucansucrases: three-dimensional structures, reactions, mechanism, α-glucan analysis and their implications in biotechnology and food applications. J. Biotechnol. 2013, 163, 250–272. 10.1016/j.jbiotec.2012.06.037. 22796091

[ref2] MacGregor E. A.; Jespersen H. M.; Svensson B. A circularly permuted alpha-amylase-type alpha/beta-barrel structure in glucan-synthesizing glucosyltransferases. FEBS Lett. 1996, 378, 263–266. 10.1016/0014-5793(95)01428-4. 8557114

[ref3] Henrissat B.; Davies G. Structural and sequence-based classification of glycoside hydrolases. Curr. Opin Struct Biol. 1997, 7, 637–644. 10.1016/S0959-440X(97)80072-3. 9345621

[ref4] Drula E.; Garron M.; Dogan S.; Lombard V.; Henrissat B.; Terrapon N. The carbohydrate-active enzyme database: functions and literature. Nucleic Acids Res. 2022, 50, D571–D577. 10.1093/nar/gkab1045. 34850161 PMC8728194

[ref5] Monchois V.; Willemot R.-M.; Monsan P. Glucansucrases: mechanism of action and structure-function relationships. FEMS Microbiol. Rev. 1999, 23, 131–151. 10.1111/j.1574-6976.1999.tb00394.x. 10234842

[ref6] Côté G. L.; Robyt J. F. The formation of alpha-D-(1----3) branch linkages by a D-glucansucrase from *Streptococcus mutans* 6715 producing a soluble D-glucan. Carbohydr. Res. 1984, 127, 95–107. 10.1016/0008-6215(84)85108-3. 6201273

[ref7] Molina M.; Cioci G.; Moulis C.; Séverac E.; Remaud-Siméon M. Bacterial α-glucan and branching sucrases from GH70 family: discovery, structure–function relationship studies and engineering. Microorganisms 2021, 9, 1607 10.3390/microorganisms9081607. 34442685 PMC8398850

[ref8] Gangoiti J.; Pijning T.; Dijkhuizen L. Biotechnological potential of novel glycoside hydrolase family 70 enzymes synthesizing α-glucans from starch and sucrose. Biotechnol Adv. 2018, 36, 196–207. 10.1016/j.biotechadv.2017.11.001. 29133008

[ref9] Miao M.; Jiang B.; Jin Z.; BeMiller J. N. Microbial starch-converting enzymes: recent insights and perspectives. Compr Rev. Food Sci. Food Saf. 2018, 17, 1238–1260. 10.1111/1541-4337.12381. 33350152

[ref10] Li X.; Wang X.; Meng X.; Dijkhuizen L.; Liu W. Structures, physico-chemical properties, production and (potential) applications of sucrose-derived α-d-glucans synthesized by glucansucrases. Carbohydr. Polym. 2020, 249, 116818 10.1016/j.carbpol.2020.116818. 32933666

[ref11] Gangoiti J.; Corwin S. F.; Lamothe L. M.; Vafiadi C.; Hamaker B. R.; Dijkhuizen L. Synthesis of novel alpha-glucans with potential health benefits through controlled glucose release in the human gastrointestinal tract. Crit. Rev. Food Sci. Nutr. 2020, 60, 123–146. 10.1080/10408398.2018.1516621. 30525940

[ref12] Vujičić-Žagar A.; Pijning T.; Kralj S.; López C. A.; Eeuwema W.; Dijkhuizen L.; Dijkstra B. W. Crystal structure of a 117 kDa glucansucrase fragment provides insight into evolution and product specificity of GH70 enzymes. Proc. Natl. Acad. Sci. U. S. A. 2010, 107, 21406–21411. 10.1073/pnas.1007531107. 21118988 PMC3003066

[ref13] Ito K.; Ito S.; Shimamura T.; Weyand S.; Kawarasaki Y.; Misaka T.; Abe K.; Kobayashi T.; Cameron A. D.; Iwata S. Crystal structure of glucansucrase from the dental caries pathogen *Streptococcus mutans*. J. Mol. Biol. 2011, 408, 177–186. 10.1016/j.jmb.2011.02.028. 21354427

[ref14] Brison Y.; Pijning T.; Malbert Y.; Fabre É; Mourey L.; Morel S.; Potocki-Véronèse G.; Monsan P.; Tranier S.; Remaud-Siméon M.; Dijkstra B. W. Functional and structural characterization of α-(1->2) branching sucrase derived from DSR-E glucansucrase. J. Biol. Chem. 2012, 287, 7915–7924. 10.1074/jbc.M111.305078. 22262856 PMC3318707

[ref15] Pijning T.; Vujičić-Žagar A.; Kralj S.; Dijkhuizen L.; Dijkstra B. W. Structure of the α-1,6/α-1,4-specific glucansucrase GTFA from *Lactobacillus reuteri* 121. Acta Crystallogr. F Struct Biol. Cryst. Commun. 2012, 68, 1448–1454. 10.1107/S1744309112044168. PMC3509963 23192022

[ref16] Claverie M.; Cioci G.; Vuillemin M.; Monties N.; Roblin P.; Lippens G.; Remaud-Siméon M.; Moulis C. Investigations on the determinants responsible for low molar mass dextran formation by DSR-M dextransucrase. ACS Catal. 2017, 7, 7106–7119. 10.1021/acscatal.7b02182.

[ref17] Molina M.; Moulis C.; Monties N.; Pizzut-Serin S.; Guieysse D.; Morel S.; Cioci G.; Remaud-Siméon M. Deciphering an undecided enzyme: investigations of the structural determinants involved in the linkage specificity of alternansucrase. ACS Catal. 2019, 9, 2222–2237. 10.1021/acscatal.8b04510.

[ref18] Schormann N.; Patel M.; Thannickal L.; Purushotham S.; Wu R.; Mieher J. L.; Wu H.; Deivanayagam C. The catalytic domains of *Streptococcus mutans* glucosyltransferases: a structural analysis. Acta Crystallogr. F Struct Biol. Commun. 2023, 79, 119–127. 10.1107/S2053230X23003199. 37158310 PMC10167749

[ref19] Leemhuis H.; Dijkman W. P.; Dobruchowska J. M.; Pijning T.; Grijpstra P.; Kralj S.; Kamerling J. P.; Dijkhuizen L. 4,6-α-Glucanotransferase activity occurs more widespread in *Lactobacillus* strains and constitutes a separate GH70 subfamily. Appl. Microbiol. Biotechnol. 2013, 97, 181–193. 10.1007/s00253-012-3943-1. 22361861 PMC3536977

[ref20] Meng X.; Gangoiti J.; Bai Y.; Pijning T.; Van Leeuwen S. S.; Dijkhuizen L. Structure-function relationships of family GH70 glucansucrase and 4,6-α-glucanotransferase enzymes, and their evolutionary relationships with family GH13 enzymes. Cell. Mol. Life Sci. 2016, 73, 2681–2706. 10.1007/s00018-016-2245-7. 27155661 PMC4919382

[ref21] Gangoiti J.; van Leeuwen S. S.; Vafiadi C.; Dijkhuizen L. The Gram-negative bacterium *Azotobacter chroococcum* NCIMB 8003 employs a new glycoside hydrolase family 70 4,6-α-glucanotransferase enzyme (GtfD) to synthesize a reuteran like polymer from maltodextrins and starch. Biochim. Biophys. Acta 2016, 1860, 1224–1236. 10.1016/j.bbagen.2016.02.005. 26868718

[ref22] Bai Y.; Gangoiti J.; Dijkstra B. W.; Dijkhuizen L.; Pijning T. Crystal structure of 4,6-α-glucanotransferase supports diet-driven evolution of GH70 Enzymes from α-amylases in oral bacteria. Structure 2017, 25, 231–242. 10.1016/j.str.2016.11.023. 28065507

[ref23] Pijning T.; Gangoiti J.; Te Poele E. M.; Börner T.; Dijkhuizen L. Insights into broad-specificity starch modification from the crystal structure of *Limosilactobacillus reuteri* NCC 2613 4,6-α-glucanotransferase GtfB. J. Agric. Food Chem. 2021, 69, 13235–13245. 10.1021/acs.jafc.1c05657. 34708648 PMC8587608

[ref24] Yang W.; Sheng L.; Chen S.; Wang L.; Su L.; Wu J. Characterization of a new 4,6-α-glucanotransferase from *Limosilactobacillus fermentum* NCC 3057 with ability of synthesizing low molecular mass isomalto-/maltopolysaccharide - ScienceDirect. Food Bioscience 2022, 46, 101514 10.1016/j.fbio.2021.101514.

[ref25] Dong J.; Bai Y.; Wang Q.; Chen Q.; Li X.; Wang Y.; Ji H.; Meng X.; Pijning T.; Svensson B.; Dijkhuizen L.; Abou Hachem M.; Jin Z. Insights into the structure-function relationship of GH70 GtfB α-glucanotransferases from the crystal structure and molecular dynamic simulation of a newly characterized *Limosilactobacillus reuteri* N1 GtfB enzyme. J. Agric. Food Chem. 2024, 72, 5391–5402. 10.1021/acs.jafc.4c00104. 38427803

[ref26] Pijning T.; Te Poele E. M.; de Leeuw T. C.; Guskov A.; Dijkhuizen L. Crystal structure of 4,6-α-glucanotransferase GtfC-ΔC from thermophilic *Geobacillus* 12AMOR1: starch transglycosylation in non-permuted GH70 Enzymes. J. Agric. Food Chem. 2022, 70, 15283–15295. 10.1021/acs.jafc.2c06394. 36442227 PMC9732880

[ref27] Gangoiti J.; Pijning T.; Dijkhuizen L. The *Exiguobacterium sibiricum* 255–15 GtfC enzyme represents a novel glycoside hydrolase 70 subfamily of 4,6-α-glucanotransferase enzymes. Appl. Environ. Microbiol. 2016, 82, 756–766. 10.1128/AEM.03420-15. 26590275 PMC4711130

[ref28] Janeček Š How many conserved sequence regions are there in the α-amylase family?. Biologia 2002, 57, 29–41.

[ref29] Giffard P. M.; Jacques N. A. Definition of a fundamental repeating unit in streptococcal glucosyltransferase glucan-binding regions and related sequences. J. Dent Res. 1994, 73, 1133–1141. 10.1177/00220345940730060201. 8046101

[ref30] Kingston K. B.; Allen D. M.; Jacques N. A. Role of the C-terminal YG repeats of the primer-dependent streptococcal glucosyltransferase, GtfJ, in binding to dextran and mutan. Microbiology (Reading) 2002, 148, 549–558. 10.1099/00221287-148-2-549. 11832518

[ref31] Shah D. S.; Joucla G.; Remaud-Siméon M.; Russell R. R. Conserved repeat motifs and glucan binding by glucansucrases of oral streptococci and *Leuconostoc mesenteroides*. J. Bacteriol. 2004, 186, 8301–8308. 10.1128/JB.186.24.8301-8308.2004. 15576779 PMC532428

[ref32] Kralj S.; van Geel-Schutten G. H.; Dondorff M. M.; Kirsanovs S.; van der Maarel M. J.; Dijkhuizen L. Glucan synthesis in the genus *Lactobacillus*: isolation and characterization of glucansucrase genes, enzymes and glucan products from six different strains. Microbiology 2004, 150, 3681–3690. 10.1099/mic.0.27321-0. 15528655

[ref33] Moulis C.; Joucla G.; Harrison D.; Fabre E.; Potocki-Veronese G.; Monsan P.; Remaud-Simeon M. Understanding the polymerization mechanism of glycoside-hydrolase family 70 glucansucrases. J. Biol. Chem. 2006, 281, 31254–31267. 10.1074/jbc.M604850200. 16864576

[ref34] Meng X.; Dobruchowska J. M.; Pijning T.; Gerwig G. J.; Kamerling J. P.; Dijkhuizen L. Truncation of domain V of the multidomain glucansucrase GTF180 of *Lactobacillus reuteri* 180 heavily impairs its polysaccharide-synthesizing ability. Appl. Microbiol. Biotechnol. 2015, 99, 5885–5894. 10.1007/s00253-014-6361-8. 25586581

[ref35] Brison Y.; Malbert Y.; Czaplicki G.; Mourey L.; Remaud-Siméon M.; Tranier S. Structural insights into the carbohydrate binding ability of an α-(1→2) branching sucrase from glycoside hydrolase family 70. J. Biol. Chem. 2016, 291, 7527–7540. 10.1074/jbc.M115.688796. 26865636 PMC4817182

[ref36] Claverie M.; Cioci G.; Vuillemin M.; Bondy P.; Remaud-Siméon M.; Moulis C. Processivity of dextransucrases synthesizing very-high-molar-mass dextran is mediated by sugar-binding pockets in domain V. J. Biol. Chem. 2020, 295, 5602–5613. 10.1074/jbc.RA119.011995. 32161118 PMC7186162

[ref37] Molina M.; Moulis C.; Monties N.; Guieysse D.; Morel S.; Cioci G.; Remaud-Siméon M. A specific oligosaccharide-binding site in the alternansucrase catalytic domain mediates alternan elongation. J. Biol. Chem. 2020, 295, 9474–9489. 10.1074/jbc.RA120.013028. 32409580 PMC7363119

[ref38] Jumper J.; Evans R.; Pritzel A.; Green T.; Figurnov M.; Ronneberger O.; Tunyasuvunakool K.; Bates R.; Žídek A.; Potapenko A.; Bridgland A.; Meyer C.; Kohl S. A. A.; Ballard A. J.; Cowie A.; Romera-Paredes B.; Nikolov S.; Jain R.; Adler J.; Back T.; Petersen S.; Reiman D.; Clancy E.; Zielinski M.; Steinegger M.; Pacholska M.; Berghammer T.; Bodenstein S.; Silver D.; Vinyals O.; Senior A. W.; Kavukcuoglu K.; Kohli P.; Hassabis D. Highly accurate protein structure prediction with AlphaFold. Nature 2021, 596, 583–589. 10.1038/s41586-021-03819-2. 34265844 PMC8371605

[ref39] Katoh K.; Rozewicki J.; Yamada K. D. MAFFT online service: multiple sequence alignment, interactive sequence choice and visualization. Brief Bioinform. 2019, 20, 1160–1166. 10.1093/bib/bbx108. 28968734 PMC6781576

[ref40] Waterhouse A. M.; Procter J. B.; Martin D. M.; Clamp M.; Barton G. J. Jalview Version 2 - a multiple sequence alignment editor and analysis workbench. Bioinformatics 2009, 25, 1189–1191. 10.1093/bioinformatics/btp033. 19151095 PMC2672624

[ref41] Heger A.; Holm L. Rapid automatic detection and alignment of repeats in protein sequences. Proteins 2000, 41, 224–237. 10.1002/1097-0134(20001101)41:2<224::AID-PROT70>3.0.CO;2-Z. 10966575

[ref42] Sievers F.; Higgins D. G. Clustal Omega for making accurate alignments of many protein sequences. Protein Sci. 2018, 27, 135–145. 10.1002/pro.3290. 28884485 PMC5734385

[ref43] Edgar R. C. MUSCLE: multiple sequence alignment with high accuracy and high throughput. Nucleic Acids Res. 2004, 32, 1792–1797. 10.1093/nar/gkh340. 15034147 PMC390337

[ref44] Robert X.; Gouet P. Deciphering key features in protein structures with the new ENDscript server. Nucleic Acids Res. 2014, 42, W320 10.1093/nar/gku316. 24753421 PMC4086106

[ref45] Crooks G. E.; Hon G.; Chandonia J.; Brenner S. E. WebLogo: A sequence logo generator. Genome Res. 2004, 14, 1188–1190. 10.1101/gr.849004. 15173120 PMC419797

[ref46] Tamura K.; Stecher G.; Kumar S. MEGA11: Molecular Evolutionary Genetics Analysis Version 11. Mol. Biol. Evol. 2021, 38, 3022–3027. 10.1093/molbev/msab120. 33892491 PMC8233496

[ref47] Varadi M.; Anyango S.; Deshpande M.; Nair S.; Natassia C.; Yordanova G.; Yuan D.; Stroe O.; Wood G.; Laydon A.; Žídek A.; Green T.; Tunyasuvunakool K.; Petersen S.; Jumper J.; Clancy E.; Green R.; Vora A.; Lutfi M.; Figurnov M.; Cowie A.; Hobbs N.; Kohli P.; Kleywegt G.; Birney E.; Hassabis D.; Velankar S. AlphaFold Protein Structure Database: massively expanding the structural coverage of protein-sequence space with high-accuracy models. Nucleic Acids Res. 2022, 50, D439–D444. 10.1093/nar/gkab1061. 34791371 PMC8728224

[ref48] van Kempen M.; Kim S. S.; Tumescheit C.; Mirdita M.; Lee J.; Gilchrist C. L. M.; Söding J.; Steinegger M. Fast and accurate protein structure search with Foldseek. Nat. Biotechnol. 2023, 1–4, 243 10.1038/s41587-023-01773-0. PMC10869269 37156916

[ref49] Krissinel E.; Henrick K. Secondary-structure matching (SSM), a new tool for fast protein structure alignment in three dimensions. Acta Crystallogr. D Biol. Crystall. 2004, 60, 2256–2268. 10.1107/S0907444904026460. 15572779

[ref50] Paysan-Lafosse T.; Blum M.; Chuguransky S.; Grego T.; Pinto B. L.; Salazar G. A.; Bileschi M. L.; Bork P.; Bridge A.; Colwell L.; Gough J.; Haft D. H.; Letunić I.; Marchler-Bauer A.; Mi H.; Natale D. A.; Orengo C. A.; Pandurangan A. P.; Rivoire C.; Sigrist C. J. A.; Sillitoe I.; Thanki N.; Thomas P. D.; Tosatto S. C. E.; Wu C. H.; Bateman A. InterPro in 2022. Nucleic Acids Res. 2023, 51, D418–D427. 10.1093/nar/gkac993. 36350672 PMC9825450

[ref51] Te Poele E. M.; van der Hoek S. E.; Chatziioannou A. C.; Gerwig G. J.; Duisterwinkel W. J.; Oudhuis L.A.A.C.M.; Gangoiti J.; Dijkhuizen L.; Leemhuis H. GtfC Enzyme of *Geobacillus* sp. 12AMOR1 represents a novel thermostable type of GH70 4,6-α-glucanotransferase that synthesizes a linear alternating (α1 → 6)/(α1 → 4) α-glucan and delays bread staling. J. Agric. Food Chem. 2021, 69, 9859–9868. 10.1021/acs.jafc.1c03475. 34427087

[ref52] Janecek S.; Svensson B.; MacGregor E. A. alpha-Amylase: an enzyme specificity found in various families of glycoside hydrolases. Cell. Mol. Life Sci. 2014, 71, 1149–1170. 10.1007/s00018-013-1388-z. 23807207 PMC11114072

[ref53] Gangoiti J.; Lamothe L.; van Leeuwen S. S.; Vafiadi C.; Dijkhuizen L. Characterization of the *Paenibacillus beijingensis* DSM 24997 GtfD and its glucan polymer products representing a new glycoside hydrolase 70 subfamily of 4,6-α-glucanotransferase enzymes. PLoS One 2017, 12, e0172622 10.1371/journal.pone.0172622. 28399167 PMC5388325

[ref54] Olvera C.; Fernandez-Vazquez J. L.; Ledezma-Candanoza L.; Lopez-Munguia A. Role of the C-terminal region of dextransucrase from *Leuconostoc mesenteroides* IBT-PQ in cell anchoring. Microbiology 2007, 153, 3994–4002. 10.1099/mic.0.2007/008854-0. 18048914

[ref55] Janecek S.; Svensson B.; Russell R. R. B. Location of repeat elements in glucansucrases of *Leuconostoc* and *Streptococcus* species. FEMS Microbiol. Lett. 2000, 192, 53–57. 10.1016/S0378-1097(00)00408-0. 11040428

[ref56] Campbell I. D.; Spitzfaden C. Building proteins with fibronectin type III modules. Structure 1994, 2, 333–337. 10.1016/S0969-2126(00)00034-4. 8081748

[ref57] Chi-Rosso G.; Gotwals P. J.; Yang J.; Ling L.; Jiang K.; Chao B.; Baker D. P.; Burkly L. C.; Fawell S. E.; Koteliansky V. E. Fibronectin type III repeats mediate RGD-independent adhesion and signaling through activated beta1 integrins. J. Biol. Chem. 1997, 272, 31447–31452. 10.1074/jbc.272.50.31447. 9395478

[ref58] Marino M.; Banerjee M.; Jonquières R.; Cossart P.; Ghosh P. GW domains of the *Listeria monocytogenes* invasion protein InlB are SH3-like and mediate binding to host ligands. EMBO J. 2002, 21, 5623–5634. 10.1093/emboj/cdf558. 12411480 PMC131055

[ref59] Dietrich C.; Li de la Sierra-Gallay I.; Masi M.; Girard E.; Dautin N.; Constantinesco-Becker F.; Tropis M.; Daffé M.; van Tilbeurgh H.; Bayan N. The C-terminal domain of *Corynebacterium glutamicum* mycoloyltransferase A is composed of five repeated motifs involved in cell wall binding and stability. Mol. Microbiol. 2020, 114, 1–16. 10.1111/mmi.14492. 32073722

[ref60] Yu L.; Qian Z.; Ge J.; Du R. Glucansucrase produced by lactic acid bacteria: structure, properties, and applications. Fermentation 2022, 8, 629–648. 10.3390/fermentation8110629.

[ref61] Hartman A. M.; Jumde V. R.; Elgaher W. A. M.; Te Poele E. M.; Dijkhuizen L.; Hirsch A. K. H. Potential dental biofilm inhibitors: dynamic combinatorial chemistry affords sugar-based molecules that target bacterial glucosyltransferase. ChemMedChem. 2021, 16, 113–123. 10.1002/cmdc.202000222. 32542998 PMC7818428

[ref62] An Y.; Tran P. L.; Yoo M.; Song H.; Park K.; Kim T.; Park J.; Woo E. The distinctive permutated domain structure of periplasmic α-amylase (MalS) from Glycoside Hydrolase Family 13 Subfamily 19. Molecules 2023, 28, 3972 10.3390/molecules28103972. 37241718 PMC10223318

[ref63] Pijning T.; Vujičić-Žagar A.; Kralj S.; Dijkhuizen L.; Dijkstra B. W. Flexibility of truncated and full-length glucansucrase GTF180 enzymes from *Lactobacillus reuteri* 180. FEBS J. 2014, 281, 2159–2171. 10.1111/febs.12769. 24597929

[ref64] Suwannarangsee S.; Moulis C.; Potocki-Veronese G.; Monsan P.; Remaud-Siméon M.; Chulalaksananukul W. Search for a dextransucrase minimal motif involved in dextran binding. FEBS Lett. 2007, 581, 4675–4680. 10.1016/j.febslet.2007.08.062. 17826770

[ref65] Greco A.; Ho J. G. S.; Lin S.-J.; Palcic M. M.; Rupnik M.; Ng K. K.-S. Carbohydrate recognition by *Clostridium difficile* toxin A. Nat. Struct. Mol. Biol. 2006, 13, 460–461. 10.1038/nsmb1084. 16622409

[ref66] Valk V.; van der Kaaij R. M.; Dijkhuizen L. The evolutionary origin and possible functional roles of FNIII domains in two *Microbacterium Aurum* B8.A granular starch degrading enzymes, and in other carbohydrate acting enzymes. Amylase 2017, 1, 1–11. 10.1515/amylase-2017-0001.

[ref67] Kurochkina N.; Guha U. SH3 domains: modules of protein-protein interactions. Biophys. Rev. 2013, 5, 29–39. 10.1007/s12551-012-0081-z. 28510178 PMC5418429

[ref68] Dionne U.; Bourgault É.; Dubé A. K.; Bradley D.; Chartier F. J. M.; Dandage R.; Dibyachintan S.; Després P. C.; Gish G. D.; Pham N. T. H.; Létourneau M.; Lambert J.-P.; Doucet N.; Bisson N.; Landry C. R. Protein context shapes the specificity of SH3 domain-mediated interactions *in vivo*. Nat. Commun. 2021, 12, 1597 10.1038/s41467-021-21873-2. 33712617 PMC7954794

[ref69] Wangpaiboon K.; Pitakchatwong C.; Panpetch P.; Charoenwongpaiboon T.; Field R. A.; Pichyangkura R. Modified properties of alternan polymers arising from deletion of SH3-like motifs in *Leuconostoc citreum* ABK-1 alternansucrase. Carbohydr. Polym. 2019, 220, 103–109. 10.1016/j.carbpol.2019.05.002. 31196527

[ref70] Nishiyama K.; Sugiyama M.; Mukai T. Adhesion properties of lactic acid bacteria on intestinal mucin. Microorganisms 2016, 4, 34 10.3390/microorganisms4030034. 27681930 PMC5039594

