# Supplementary material for: Unprecedented Diversity of the Glycoside Hydrolase Family 70: A Comprehensive Analysis of Sequence, Structure, and Function
Source: J Agric Food Chem. 2024 Jul 18;72(30):16911–29. doi: 10.1021/acs.jafc.4c04807 (PMC11299179; doi:10.1021/acs.jafc.4c04807)
Supplement: Supplementary file 1 — jf4c04807_si_001.pdf [file jf4c04807_si_001.pdf]

SUPPORTING INFORMATION

Unprecedented Diversity of the Glycoside Hydrolase family 70 - A Comprehensive Analysis of Sequence, Structure and Function

Tjaard Pijning[1]* and Lubbert Dijkhuizen[2, 3]

[1]*Biomolecular X-ray Crystallography, Groningen Biomolecular Sciences and Biotechnology Institute (GBB), University of Groningen, Nijenborgh 7, 9747 AG Groningen, The Netherlands*

[2]*Microbial Physiology, Groningen Biomolecular Sciences and Biotechnology Institute (GBB), University of Groningen, Nijenborgh 7, 9747 AG Groningen, The Netherlands*

[3]*CarbExplore Research B.V., Zernikelaan 8, 9747 AA Groningen, The Netherlands. ORCID: 0000-0003-2312-7162*

**Corresponding author: Tjaard Pijning, Biomolecular X-ray Crystallography, Groningen Biomolecular Sciences and Biotechnology Institute (GBB), University of Groningen, Nijenborgh 7, 9747 AG Groningen, The Netherlands, tel.: +31631921948. Email: t.pijning@rug.nl. ORCID: 0000-0003-4107-366*

This file includes:

Table S1. Representative set of 259 GH70 enzymes used in this study.

Figure S1. Sequence logos of loop A2 residues in the first 208 (top) and last 51 (bottom) representative GH70 sequences.

Figure S2. Schematic domain organization of 259 GH70 enzymes used in this study.

Figure S3. FNIII-type auxiliary domains in GH70.

Figure S4. SH3-type auxiliary domains in GH70.

Figure S5. MucBP-type auxiliary domains in GH70.

Figure S6. bIG-type auxiliary domains in GH70.

Figure S7. LGFP-type auxiliary domains in GH70.

Figure S8. $(\beta_3\alpha)_3$-type auxiliary domains in GH70.

Figure S9. Small β/α-type auxiliary domains in GH70.

Figure S10. Occurrence/distribution of bacterial origin, enzyme specificity and auxiliary domain type.

Table S2. Chronological overview of review papers dealing with GH70 enzymes and their products.

Supplemental references.

Table S1.

Table S1. Representative set of 259 GH70 enzymes used in this study. For the 4 entries with a partial sequence, the missing N- or C-terminal segment is indicated with (p) at the left or right side, resp. The column “Seq. id. (%)” lists the sequence identity after alignment of the A/B/C/IV core domains; when the sequence contains two catalytic entities, values are given for each (CD1 and CD2). The column “Spec./char.” lists the (predicted) enzyme specificity; those that have been characterized are indicated by an asterisk (*). For entries for which one or more crystal structures are available, only one is listed.

**No.**	**Accession**	***Organism***	**Phylum**	**Family**	**Genus**	**Length**	**Seq. id. CD1/CD2 (%)**	**Spec./ char.**	**Name**	**PDB**	**Ref. no.**
1	Q5SBN3	*Limosilactobacillus reuteri* 180	*Bacillota*	*Lactobacillaceae*	*Lactobacillus*	1772	100,0	GS *	LrGtf180	3KLK	1, 2
2	WP_226062047.1	*Limosilactobacillus reuteri*	*Bacillota*	*Lactobacillaceae*	*Lactobacillus*	1786	98,8	GS			
3	WP_231794622.1	*Limosilactobacillus caviae*	*Bacillota*	*Lactobacillaceae*	*Lactobacillus*	1741	82,8	GS			
4	WP_225430867.1	*Limosilactobacillus reuteri*	*Bacillota*	*Lactobacillaceae*	*Lactobacillus*	1780	73,0	GS			
5	A9Q0J1	*Limosilactobacillus reuteri* TMW 1.106	*Bacillota*	*Lactobacillaceae*	*Lactobacillus*	1782	73,0	GS *	Gtf106A		3
6	Q5SBL9	*Limosilactobacillus reuteri* 121	*Bacillota*	*Lactobacillaceae*	*Lactobacillus*	1781	72,7	GS *	GtfA	4AMC	4-6
7	WP_239457077.1	*Limosilactobacillus ingluviei*	*Bacillota*	*Lactobacillaceae*	*Lactobacillus*	1781	68,1	GS			
8	Q4JCS4	*Limosilactobacillus reuteri* ATCC 55730	*Bacillota*	*Lactobacillaceae*	*Lactobacillus*	1781	68,1	GS *	GtfO		7
9	WP_152744819.1	*Limosilactobacillus reuteri*	*Bacillota*	*Lactobacillaceae*	*Lactobacillus*	1772	69,3	GS			
10	Q5SBN0	*Limosilactobacillus reuteri* ML1	*Bacillota*	*Lactobacillaceae*	*Lactobacillus*	1772	69,0	GS *	GtfML1		8
11	WP_262349203.1	*Limosilactobacillus fermentum*	*Bacillota*	*Lactobacillaceae*	*Lactobacillus*	1638	69,2	GS			
12	WP_082601335.1	*Limosilactobacillus gastricus*	*Bacillota*	*Lactobacillaceae*	*Lactobacillus*	1094	68,4	GS			
13	WP_261909402.1	*Liquorilactobacillus satsumensis*	*Bacillota*	*Lactobacillaceae*	*Lactobacillus*	1709	62,5	GS			
14	WP_057741771.1	*Liquorilactobacillus cappilatus*	*Bacillota*	*Lactobacillaceae*	*Lactobacillus*	1493	60,6	GS			
15	WP_057875231.1	*Liquorilactobacillus aquaticus*	*Bacillota*	*Lactobacillaceae*	*Lactobacillus*	1273	60,3	GS			
16	WP_260344977.1	*Periweissella beninensis*	*Bacillota*	*Lactobacillaceae*	*Periweissella*	1233	62,2	GS			
17	WP_057738211.1	*Liquorilactobacillus uvarum*	*Bacillota*	*Lactobacillaceae*	*Lactobacillus*	1058	61,2	GS			
18	WP_057876534.1	*Liquorilactobacillus aquaticus*	*Bacillota*	*Lactobacillaceae*	*Lactobacillus*	1058	60,6	GS			
19	WP_051993368.1	*Liquorilactobacillus sucicola*	*Bacillota*	*Lactobacillaceae*	*Lactobacillus*	1070	58,9	GS			

20	WP_057746024.1	*Liquorilactobacillus cappilatus*	*Bacillota*	*Lactobacillaceae*	*Lactobacillus*	1068	58,9	GS		
21	WP_056961808.1	*Liquorilactobacillus satsumensis*	*Bacillota*	*Lactobacillaceae*	*Lactobacillus*	1053	60,1	GS		
22	WP_056990607.1	*Liquorilactobacillus mali*	*Bacillota*	*Lactobacillaceae*	*Lactobacillus*	1058	60,0	GS		
23	WP_223389705.1	*Liquorilactobacillus hordei*	*Bacillota*	*Lactobacillaceae*	*Lactobacillus*	1508	58,9	GS		
24	WP_136344491.1	*Ligilactobacillus animalis*	*Bacillota*	*Lactobacillaceae*	*Lactobacillus*	1508	59,9	GS		
25	WP_010690264.1	*Ligilactobacillus animalis*	*Bacillota*	*Lactobacillaceae*	*Lactobacillus*	1651	61,0	GS		
26	WP_257576753.1	*Ligilactobacillus apodemi*	*Bacillota*	*Lactobacillaceae*	*Lactobacillus*	1703	57,3	GS		
27	WP_186432140.1	*Oenococcus oeni*	*Bacillota*	*Lactobacillaceae*	*Oenococcus*	1941	57,5	GS		
28	WP_127824245.1	*Oenococcus* UCMA16435	*Bacillota*	*Lactobacillaceae*	*Oenococcus*	1935	57,8	GS		
29	WP_220730315.1	*Leuconostoc rapi*	*Bacillota*	*Lactobacillaceae*	*Leuconostoc*	1793	56,1	GS		
30	WP_243155631.1	*Leuconostoc gelidum*	*Bacillota*	*Lactobacillaceae*	*Leuconostoc*	1877	54,3	GS		
31	WP_220740687.1	*Leuconostoc miyukkimchii*	*Bacillota*	*Lactobacillaceae*	*Leuconostoc*	1827	54,9	GS		
32	WP_193324688.1	*Leuconostoc citreum*	*Bacillota*	*Lactobacillaceae*	*Leuconostoc*	1935	53,3	GS		
33	D5FS20	*Leuconostoc citreum* B110-1-2 D5FS20	*Bacillota*	*Lactobacillaceae*	*Leuconostoc*	1527	53,1	GS *	Dsr-F	9
34	WP_201331104.1	*Lactobacillus nasalidis*	*Bacillota*	*Lactobacillaceae*	*Lactobacillus*	1185	60,9	GS		
35	WP_052933770.1	*Lactobacillus delbrueckii*	*Bacillota*	*Lactobacillaceae*	*Lactobacillus*	1179	60,5	GS		
36	WP_244661437.1	*Lactobacillus nasalidis*	*Bacillota*	*Lactobacillaceae*	*Lactobacillus*	(p) 966	59,2	GS		
37	WP_081372331.1	*Lactobacillus delbrueckii*	*Bacillota*	*Lactobacillaceae*	*Lactobacillus*	1202	60,0	GS		
38	WP_074027723.1	*Lactobacillus delbrueckii*	*Bacillota*	*Lactobacillaceae*	*Lactobacillus*	1214	60,2	GS		
39	WP_223318597.1	*Leuconostoc mesenteroides*	*Bacillota*	*Lactobacillaceae*	*Leuconostoc*	1527	56,8	GS		
40	Q8KRE1	*Leuconostoc mesenteroides* LCC4	*Bacillota*	*Lactobacillaceae*	*Leuconostoc*	1527	56,3	GS *	DsrD	10
41	J7FZE3	*Leuconostoc mesenteroides* KIBGE-IB-22	*Bacillota*	*Lactobacillaceae*	*Leuconostoc*	1527	56,4	GS *	DsrN	11
42	Q2I2N5	*Leuconostoc mesenteroides* 0326	*Bacillota*	*Lactobacillaceae*	*Leuconostoc*	1527	56,3	GS *	DexYG	12
43	Q6TXV4	*Leuconostoc mesenteroides* L0309	*Bacillota*	*Lactobacillaceae*	*Leuconostoc*	1522	56,5	GS *	DsrX	13
44	Q9ZAR4	*Leuconostoc mesenteroides* NRRL B-512F	*Bacillota*	*Lactobacillaceae*	*Leuconostoc*	1527	56,1	GS *	DsrS	14
45	WP_148605829.1	*Leuconostoc litchii*	*Bacillota*	*Lactobacillaceae*	*Leuconostoc*	1557	54,7	GS		
46	WP_010007068.1	*Leuconostoc fallax*	*Bacillota*	*Lactobacillaceae*	*Leuconostoc*	1543	56,4	GS		
47	WP_220739689.1	*Leuconostoc miyukkimchii*	*Bacillota*	*Lactobacillaceae*	*Leuconostoc*	1531	54,9	GS		
48	WP_210729352.1	*Leuconostoc holzapfelii*	*Bacillota*	*Lactobacillaceae*	*Leuconostoc*	1482	54,9	GS		
49	C7DT60	*Leuconostoc lactis EG001*	*Bacillota*	*Lactobacillaceae*	*Leuconostoc*	1500	54,4	GS *		15
50	WP_146972579.1	*Leuconostoc citreum*	*Bacillota*	*Lactobacillaceae*	*Leuconostoc*	1505	54,9	GS		
51	A9ZRX3	*Leuconostoc citreum*	*Bacillota*	*Lactobacillaceae*	*Leuconostoc*	1477	54,6	GS *	LcDS	16

52	D2CFL0	*Leuconostoc mesenteroides* B-1299CB4	*Bacillota*	*Lactobacillaceae*	*Leuconostoc*	1505	54,6	GS *	DsrBCB4	17
53	Q9L466	*Leuconostoc mesenteroides* B-1355	*Bacillota*	*Lactobacillaceae*	*Leuconostoc*	1477	54,6	GS *	DsrC	18
54	O52224	*Leuconostoc mesenteroides* B-1299	*Bacillota*	*Lactobacillaceae*	*Leuconostoc*	1508	54,5	GS *	DsrB	19
55	Q9EZH5	*Leuconostoc mesenteroides* B-742CB	*Bacillota*	*Lactobacillaceae*	*Leuconostoc*	1508	54,6	GS *	Dsrb742	20
56	WP_128686848.1	*Oenococcus sicerae*	*Bacillota*	*Lactobacillaceae*	*Oenococcus*	1524	56,5	GS		
57	WP_028291708.1	*Oenococcus kitaharae* DSM 17330	*Bacillota*	*Lactobacillaceae*	*Oenococcus*	1511	55,3	GS *	Dsr-OK	21
58	WP_252767194.1	*Fructilactobacillus* KI4 B1	*Bacillota*	*Lactobacillaceae*	*Lactobacillus*	1612	53,2	GS		
59	WP_252750234.1	*Fructilactobacillus* KI16 H9	*Bacillota*	*Lactobacillaceae*	*Lactobacillus*	1636	52,7	GS		
60	WP_241655762.1	*Enterococcus faecium*	*Bacillota*	*Enterococcaceae*	*Enterococcus*	1724	53,7	GS		
61	L8BSI8	*Latilactobacillus curvatus* TMW 1.624	*Bacillota*	*Lactobacillaceae*	*Lactobacillus*	1697	53,5	GS *	Gtf1624	3
62	Q5SBM3	*Latilactobacillus sakei*	*Bacillota*	*Lactobacillaceae*	*Lactobacillus*	1595	53,5	GS *	Gtf-Kg15	8
63	WP_242458253.1	*Weissella confusa*	*Bacillota*	*Lactobacillaceae*	*Weissella*	1423	55,8	GS		
64	A0A482PV50	*Weissella confusa* VTT E-90392	*Bacillota*	*Lactobacillaceae*	*Weissella*	1418	55,2	GS *	Wc392-DSR	22
65	A0A5E8F621	*Weissella confusa* LBAE C39-2	*Bacillota*	*Lactobacillaceae*	*Weissella*	1412	55,7	GS *	DSRC39-2	23
66	A0A0F6TP16	*Weissella confusa* Cab3	*Bacillota*	*Lactobacillaceae*	*Weissella*	1401	55,7	GS *	WcCAB3-DSR	24
67	WP_231898404.1	*Weissella cibaria*	*Bacillota*	*Lactobacillaceae*	*Weissella*	1462	54,6	GS		
68	D2XRA2	*Weissella cibaria* LBAE-K39	*Bacillota*	*Lactobacillaceae*	*Weissella*	1445	53,7	GS *	DsrK39	25
69	Q5SBM6	*Limosilactobacillus fermentum* KG3	*Bacillota*	*Lactobacillaceae*	*Lactobacillus*	1463	53,5	GS *	GtfKg3	8
70	B9UNL6	*Weissella cibaria* CMU	*Bacillota*	*Lactobacillaceae*	*Weissella*	1472	53,8	GS *	DsrwC	26
71	WP_200136072.1	*Leuconostoc multispecies*	*Bacillota*	*Lactobacillaceae*	*Leuconostoc*	1487	54,5	GS		
72	WP_205143387.1	*Periweissella beninensis*	*Bacillota*	*Lactobacillaceae*	*Periweissella*	1620	52,6	GS		
73	WP_260355020.1	*Leuconostoc suionicum*	*Bacillota*	*Lactobacillaceae*	*Leuconostoc*	1511	53,0	GS		
74	WP_052037835.1	*Leuconostoc mesenteroides*	*Bacillota*	*Lactobacillaceae*	*Leuconostoc*	1511	52,6	GS		
75	WP_203617611.1	*Fructobacillus tropaeoli*	*Bacillota*	*Lactobacillaceae*	*Fructobacillus*	1606	53,2	GS		
76	WP_248720544.1	*Convivina intestini*	*Bacillota*	*Lactobacillaceae*	*Convivina*	1631	49,8	GS		
77	WP_188351245.1	*Leuconostoc pseudomesenteroides*	*Bacillota*	*Lactobacillaceae*	*Leuconostoc*	1483	55,6	GS		
78	WP_014975050.1	*Leuconostoc carnosum*	*Bacillota*	*Lactobacillaceae*	*Leuconostoc*	2824	54,0 / 51,4	GS-BrS		
79	WP_243129707.1	*Leuconostoc citreum*	*Bacillota*	*Lactobacillaceae*	*Leuconostoc*	1707	54,2	GS		
80	WP_150260610.1	*Leuconostoc carnosum*	*Bacillota*	*Lactobacillaceae*	*Leuconostoc*	1461	56,4	GS		
81	WP_261907634.1	*Liquorilactobacillus satsumensis*	*Bacillota*	*Lactobacillaceae*	*Lactobacillus*	1476	54,0	GS		
82	WP_228888120.1	*Liquorilactobacillus satsumensis*	*Bacillota*	*Lactobacillaceae*	*Lactobacillus*	1791	54,0	GS		
83	WP_261912693.1	*Lentilactobacillus hilgardii*	*Bacillota*	*Lactobacillaceae*	*Lactobacillus*	1545	56,0	GS		

84	Q5SBM8	*Lentilactobacillus parabuchneri* 33	*Bacillota*	*Lactobacillaceae*	*Lactobacillus*	1561	55,4	GS *	Gtf33		8
85	WP_231458450.1	*Pediococcus pentosaceus*	*Bacillota*	*Lactobacillaceae*	*Pediococcus*	1505	53,5	GS			
86	WP_263282894.1	*Lentilactobacillus hilgardii*	*Bacillota*	*Lactobacillaceae*	*Lactobacillus*	1400	55,4	GS			
87	WP_220740895.1	*Leuconostoc miyukkimchii*	*Bacillota*	*Lactobacillaceae*	*Leuconostoc*	1567	54,2	GS			
88	WP_139988371.1	*Leuconostoc citreum*	*Bacillota*	*Lactobacillaceae*	*Leuconostoc*	1495	51,2	GS			
89	B1N0B6	*Leuconostoc citreum* KM20	*Bacillota*	*Lactobacillaceae*	*Leuconostoc*	1495	50,8	GS *	DexT		27
90	Q48756	*Leuconostoc mesenteroides* B-1299	*Bacillota*	*Lactobacillaceae*	*Leuconostoc*	1290	49,1	GS *	DsrA		28
91	WP_252780198.1	*Fructilactobacillus ixorae*	*Bacillota*	*Lactobacillaceae*	*Lactobacillus*	1416	53,0	GS			
92	WP_252797321.1	*Fructilactobacillus hinvesii*	*Bacillota*	*Lactobacillaceae*	*Lactobacillus*	1906	52,7	GS			
93	WP_248574655.1	*Leuconostoc* MTCC 10508	*Bacillota*	*Lactobacillaceae*	*Leuconostoc*	1488	54,4	GS			
94	Q69A94	*Leuconostoc mesenteroides* IBT-PQ	*Bacillota*	*Lactobacillaceae*	*Leuconostoc*	1454	53,3	GS *	DsrP		29
95	WP_216761399.1	*Leuconostoc fallax*	*Bacillota*	*Lactobacillaceae*	*Leuconostoc*	1604	54,0	GS			
96	WP_174249168.1	*Leuconostoc gelidum*	*Bacillota*	*Lactobacillaceae*	*Leuconostoc*	1528	54,4	GS			
97	WP_135197408.1	*Leuconostoc carnosum*	*Bacillota*	*Lactobacillaceae*	*Leuconostoc*	1537	53,3	GS			
98	WP_204769824.1	*Leuconostoc rapi*	*Bacillota*	*Lactobacillaceae*	*Leuconostoc*	1554	52,6	GS			
99	WP_186432386.1	*Oenococcus oeni*	*Bacillota*	*Lactobacillaceae*	*Oenococcus*	1323	53,8	GS			
100	WP_128685690.1	*Oenococcus sicerae*	*Bacillota*	*Lactobacillaceae*	*Oenococcus*	1352	52,8	GS			
101	WP_268872824.1	*Liquorilactobacillus aquaticus*	*Bacillota*	*Lactobacillaceae*	*Lactobacillus*	1506	52,8	GS			
102	WP_057738872.1	*Liquorilactobacillus uvarum*	*Bacillota*	*Lactobacillaceae*	*Lactobacillus*	1102 (p)	52,1	GS			
103	WP_081778173.1	*Liquorilactobacillus sucicola*	*Bacillota*	*Lactobacillaceae*	*Lactobacillus*	1583	52,0	GS			
104	WP_057743768.1	*Liquorilactobacillus capillatus*	*Bacillota*	*Lactobacillaceae*	*Lactobacillus*	1516	51,5	GS			
105	WP_248720560.1	*Convivina intestini*	*Bacillota*	*Lactobacillaceae*	*Convivina*	1467	55,1	GS			
106	WP_252443191.1	*Fructobacillus* W13	*Bacillota*	*Lactobacillaceae*	*Fructobacillus*	1675	52,9	GS			
107	WP_057491792.1	*Streptococcus orisasini*	*Bacillota*	*Streptococcaceae*	*Streptococcus*	1482	54,5	GS			
108	WP_075346439.1	*Streptococcus caviae*	*Bacillota*	*Streptococcaceae*	*Streptococcus*	1414	53,9	GS			
109	WP_018029790.1	*Streptococcus ferus*	*Bacillota*	*Streptococcaceae*	*Streptococcus*	1392	53,8	GS			
110	WP_014678014.1	*Streptococcus mutans*	*Bacillota*	*Streptococcaceae*	*Streptococcus*	1477	53,0	GS			
111	P08987	*Streptococcus mutans* ATCC 700610	*Bacillota*	*Streptococcaceae*	*Streptococcus*	1476	52,7	GS *	Gtf-I	8FG8	30, 31
112	P13470	*Streptococcus mutans* ATCC 700610	*Bacillota*	*Streptococcaceae*	*Streptococcus*	1455	52,9	GS *	Gtf-SI	3AIB	32, 33
113	WP_003079439.1	*Streptococcus macacae*	*Bacillota*	*Streptococcaceae*	*Streptococcus*	1418	51,7	GS			
114	WP_003081055.1	*Streptococcus macacae*	*Bacillota*	*Streptococcaceae*	*Streptococcus*	1448	51,9	GS			
115	WP_019770806.1	*Streptococcus sobrinus*	*Bacillota*	*Streptococcaceae*	*Streptococcus*	1590	52,2	GS			

116	Q55263	*Streptococcus sobrinus*	*Bacillota*	*Streptococcaceae*	*Streptococcus*	1590	51,9	GS *	GtfI		34
117	P11001	*Streptococcus downei* MFE 28	*Bacillota*	*Streptococcaceae*	*Streptococcus*	1597	51,9	GS *	GtfI		35
118	P27470	*Streptococcus sobrinus*	*Bacillota*	*Streptococcaceae*	*Streptococcus*	1592	51,5	GS *	GTF-I		36
119	WP_249551167.1	*Streptococcus gallolyticus*	*Bacillota*	*Streptococcaceae*	*Streptococcus*	1545	52,3	GS			
120	WP_074669003.1	*Streptococcus equinus*	*Bacillota*	*Streptococcaceae*	*Streptococcus*	1445	52,1	GS			
121	WP_074486119.1	*Streptococcus henryi*	*Bacillota*	*Streptococcaceae*	*Streptococcus*	1475	51,2	GS			
122	WP_018364919.1	*Streptococcus caballi*	*Bacillota*	*Streptococcaceae*	*Streptococcus*	1622	52,6	GS			
123	WP_149561278.1	*Streptococcus salivarius*	*Bacillota*	*Streptococcaceae*	*Streptococcus*	1599	51,0	GS			
124	Q00599	*Streptococcus salivarius* ATCC 25975	*Bacillota*	*Streptococcaceae*	*Streptococcus*	1599	50,6	GS *	GtfK		37
125	WP_205016717.1	*Streptococcus saliviloxodontae*	*Bacillota*	*Streptococcaceae*	*Streptococcus*	1623	49,9	GS			
126	WP_205009160.1	*Streptococcus loxodontisalivarius*	*Bacillota*	*Streptococcaceae*	*Streptococcus*	1620	50,4	GS			
127	WP_019777743.1	*Streptococcus sobrinus*	*Bacillota*	*Streptococcaceae*	*Streptococcus*	1508	50,9	GS			
128	WP_019780969.1	*Streptococcus sobrinus*	*Bacillota*	*Streptococcaceae*	*Streptococcus*	1554	49,6	GS			
129	Q8KZL5	*Streptococcus sobrinus* B13N	*Bacillota*	*Streptococcaceae*	*Streptococcus*	1554	49,6	GS *	GtfU		36
130	WP_165213336.1	*Streptococcus* ZJ373	*Bacillota*	*Streptococcaceae*	*Streptococcus*	1542	50,2	GS			
131	WP_082753031.1	*Streptococcus* DD12	*Bacillota*	*Streptococcaceae*	*Streptococcus*	1540	51,8	GS			
132	WP_008534376.1	*Streptococcus* C150	*Bacillota*	*Streptococcaceae*	*Streptococcus*	1492	50,7	GS			
133	WP_143568112.1	*Streptococcus salivarius*	*Bacillota*	*Streptococcaceae*	*Streptococcus*	1490	49,1	GS			
134	WP_205016800.1	*Streptococcus saliviloxodontae*	*Bacillota*	*Streptococcaceae*	*Streptococcus*	1648	49,8	GS			
135	WP_143568111.1	*Streptococcus salivarius*	*Bacillota*	*Streptococcaceae*	*Streptococcus*	1520	48,4	GS			
136	Q00600	*Streptococcus salivarius* ATCC 25975	*Bacillota*	*Streptococcaceae*	*Streptococcus*	1518	48,6	GS *	GtfJ		38
137	WP_205016801.1	*Streptococcus saliviloxodontae*	*Bacillota*	*Streptococcaceae*	*Streptococcus*	1510	51,6	GS			
138	WP_205008850.1	*Streptococcus loxodontisalivarius*	*Bacillota*	*Streptococcaceae*	*Streptococcus*	1512	51,5	GS			
139	WP_168750373.1	*Streptococcus mutans*	*Bacillota*	*Streptococcaceae*	*Streptococcus*	1462	52,1	GS			
140	P49331	*Streptococcus mutans* ATCC 700610	*Bacillota*	*Streptococcaceae*	*Streptococcus*	1462	51,5	GS *	Gtf-S	8FN5	31, 39
141	WP_003079947.1	*Streptococcus macacae*	*Bacillota*	*Streptococcaceae*	*Streptococcus*	1471	51,7	GS			
142	WP_027976201.1	*Streptococcus devriesei*	*Bacillota*	*Streptococcaceae*	*Streptococcus*	1465	52,3	GS			
143	WP_018029925.1	*Streptococcus ferus*	*Bacillota*	*Streptococcaceae*	*Streptococcus*	1480	51,2	GS			
144	WP_002929608.1	*Streptococcus sanguinis*	*Bacillota*	*Streptococcaceae*	*Streptococcus*	1557	53,2	GS			
145	Q9LCH3	*Streptococcus oralis*	*Bacillota*	*Streptococcaceae*	*Streptococcus*	1575	52,1	GS *	GtfR		40
146	A8AVK3	*Streptococcus gordonii*	*Bacillota*	*Streptococcaceae*	*Streptococcus*	1576	52,4	GS *	GtfG		41
147	WP_209551424.1	*Streptococcus panodentis*	*Bacillota*	*Streptococcaceae*	*Streptococcus*	1574	51,9	GS			

148	WP_245335104.1	*Streptococcus oricebi*	*Bacillota*	*Streptococcaceae*	*Streptococcus*	1494	51,2	GS			
149	WP_082854413.1	*Streptococcus pantholopis*	*Bacillota*	*Streptococcaceae*	*Streptococcus*	1507	53,1	GS			
150	WP_070847249.1	*Streptococcus* HMSC068F04	*Bacillota*	*Streptococcaceae*	*Streptococcus*	1573	51,4	GS			
151	Q55265	*Streptococcus salivarius* ATCC 25975	*Bacillota*	*Streptococcaceae*	*Streptococcus*	1577	50,9	GS *	GtfM		38
152	WP_201041480.1	*Streptococcus* 21.1	*Bacillota*	*Streptococcaceae*	*Streptococcus*	1449	53,9	GS			
153	Q55264	*Streptococcus salivarius* ATCC 25975	*Bacillota*	*Streptococcaceae*	*Streptococcus*	1449	52,7	GS *	GtfL		38
154	WP_002888669.1	*Streptococcus salivarius*	*Bacillota*	*Streptococcaceae*	*Streptococcus*	1463	53,8	GS			
155	WP_066916356.1	*Streptococcus* DD12	*Bacillota*	*Streptococcaceae*	*Streptococcus*	1395	53,1	GS			
156	WP_066916358.1	*Streptococcus* DD12	*Bacillota*	*Streptococcaceae*	*Streptococcus*	1464	51,5	GS			
157	WP_201710270.1	*Streptococcus salivarius*	*Bacillota*	*Streptococcaceae*	*Streptococcus*	1585	52,4	GS			
158	WP_004228132.1	*Streptococcus criceti*	*Bacillota*	*Streptococcaceae*	*Streptococcus*	1393	52,3	GS			
159	WP_019785141.1	*Streptococcus sobrinus*	*Bacillota*	*Streptococcaceae*	*Streptococcus*	1360	52,7	GS			
160	WP_019795751.1	*Streptococcus sobrinus*	*Bacillota*	*Streptococcaceae*	*Streptococcus*	1369	50,9	GS			
161	P29336	*Streptococcus downei* MFE 28	*Bacillota*	*Streptococcaceae*	*Streptococcus*	1365	50,6	GS *	GtfS		42
162	WP_165213351.1	*Streptococcus* J373	*Bacillota*	*Streptococcaceae*	*Streptococcus*	1338	51,8	GS			
163	WP_051394930.1	*Leuconostoc mesenteroides*	*Bacillota*	*Lactobacillaceae*	*Leuconostoc*	1323	49,6	GS			
164	WP_147011350.1	*Leuconostoc mesenteroides*	*Bacillota*	*Lactobacillaceae*	*Leuconostoc*	1498	49,7	GS			
165	WP_219054040.1	*Lactobacillus* Sy-1	*Bacillota*	*Lactobacillaceae*	*Lactobacillus*	1865	51,8	GS			
166	WP_260117219.1	*Nicoliella spurrieriana*	*Bacillota*	*Lactobacillaceae*	*Nicoliella*	1900	51,1	GS			
167	WP_260116902.1	*Nicoliella spurrieriana*	*Bacillota*	*Lactobacillaceae*	*Nicoliella*	1282	50,7	GS			
168	WP_054605012.1	*Apilactobacillus kunkeei*	*Bacillota*	*Lactobacillaceae*	*Lactobacillus*	1419	50,6	GS			
169	WP_053792989.1	*Apilactobacillus kunkeei*	*Bacillota*	*Lactobacillaceae*	*Lactobacillus*	1433	50,8	GS			
170	WP_140967679.1	*Apilactobacillus kunkeei*	*Bacillota*	*Lactobacillaceae*	*Lactobacillus*	1430	50,1	GS			
171	WP_220382206.1	*Apilactobacillus kunkeei*	*Bacillota*	*Lactobacillaceae*	*Lactobacillus*	1286	47,7	GS			
172	WP_140967681.1	*Apilactobacillus kunkeei*	*Bacillota*	*Lactobacillaceae*	*Lactobacillus*	2954	48,7 / 49,4	GS-BrS			
173	WP_054605837.1	*Apilactobacillus kunkeei*	*Bacillota*	*Lactobacillaceae*	*Lactobacillus*	1853	48,2	GS			
174	WP_089940200.1	*Convivina intestini*	*Bacillota*	*Lactobacillaceae*	*Convivina*	1294	50,6	GS			
176	WP_248715884.1	*Convivina* LMG 32447	*Bacillota*	*Lactobacillaceae*	*Convivina*	1480	49,3	GS			
176	WP_260116903.1	*Nicoliella spurrieriana*	*Bacillota*	*Lactobacillaceae*	*Nicoliella*	875	46,4	GS			
177	WP_219053759.1	*Lactobacillus* Sy-1	*Bacillota*	*Lactobacillaceae*	*Lactobacillus*	878	47,2	GS			
178	WP_036061303.1	*Leuconostoc citreum*	*Bacillota*	*Lactobacillaceae*	*Leuconostoc*	1786 (p)	48,8	GS			
179	A0A2H4A2M1	*Leuconostoc citreum* NRRL B-1299	*Bacillota*	*Lactobacillaceae*	*Leuconostoc*	1293	48,4	GS *	Dsr-M	5LFC	43, 44

180	WP_252762729.1	*Leuconostoc fallax*	*Bacillota*	*Lactobacillaceae*	*Leuconostoc*	1936	51,3	GS			
181	WP_141266701.1	*Leuconostoc mesenteroides*	*Bacillota*	*Lactobacillaceae*	*Leuconostoc*	2811	55,2 / 51,6	GS-BrS			
182	WP_014975050.1	*Leuconostoc carnosum*	*Bacillota*	*Lactobacillaceae*	*Leuconostoc*	2824	53,5 / 51,4	GS-BrS			
183	WP_203617613.1	*Fructobacillus tropaeoli*	*Bacillota*	*Lactobacillaceae*	*Fructobacillus*	1669	50,7	BrS			
184	WP_260117220.1	*Nicoliella spurrieriana*	*Bacillota*	*Lactobacillaceae*	*Nicoliella*	1912	50,8	BrS			
185	WP_059394329.1	*Fructobacillus tropaeoli*	*Bacillota*	*Lactobacillaceae*	*Fructobacillus*	1672 (p)	50,6	BrS			
186	WP_248720543.1	*Convivina intestini*	*Bacillota*	*Lactobacillaceae*	*Convivina*	1567	48,9	BrS			
187	WP_248605444.1	*Apilactobacillus kunkeei*	*Bacillota*	*Lactobacillaceae*	*Lactobacillus*	2806	47,4 / 49,9	GS-BrS			
188	WP_220382103.1	*Apilactobacillus kunkeei*	*Bacillota*	*Lactobacillaceae*	*Lactobacillus*	2926	47,7 / 49,0	GS-BrS			
189	WP_054608464.1	*Apilactobacillus kunkeei*	*Bacillota*	*Lactobacillaceae*	*Lactobacillus*	1318	49,1	BrS			
190	WP_140967680.1	*Apilactobacillus kunkeei*	*Bacillota*	*Lactobacillaceae*	*Lactobacillus*	1134	48,5	BrS			
191	WP_260167844.1	*Leuconostoc mesenteroides*	*Bacillota*	*Lactobacillaceae*	*Leuconostoc*	1598	50,4	BrS			
192	WP_165980462.1	*Leuconostoc citreum*	*Bacillota*	*Lactobacillaceae*	*Leuconostoc*	1877	50,9	BrS			
193	CDX66896	*Leuconostoc citreum*	*Bacillota*	*Lactobacillaceae*	*Leuconostoc*	1877	50,6	BrS *	Brs-A		43
194	WP_243284657.1	*Leuconostoc citreum* NRRL B-1299	*Bacillota*	*Lactobacillaceae*	*Leuconostoc*	2833	53,6 / 51,3	GS-BrS			
195	G8XR50	*Leuconostoc citreum* NRRL B-1299	*Bacillota*	*Lactobacillaceae*	*Leuconostoc*	2836	53,4 / 50,9	GS-BrS *	DsrE	3TTO	45, 46
196	WP_010006776.1	*Leuconostoc fallax*	*Bacillota*	*Lactobacillaceae*	*Leuconostoc*	1774	50,7	BrS			
197	WP_149333926.1	*Leuconostoc citreum*	*Bacillota*	*Lactobacillaceae*	*Leuconostoc*	1888	48,1	BrS			
198	WP_080732772.1	*Leuconostoc mesenteroides*	*Bacillota*	*Lactobacillaceae*	*Leuconostoc*	1583	48,0	BrS			
199	WP_155246464.1	*Leuconostoc mesenteroides*	*Bacillota*	*Lactobacillaceae*	*Leuconostoc*	1268	48,5	BrS			
200	WP_192269123.1	*Leuconostoc mesenteroides*	*Bacillota*	*Lactobacillaceae*	*Leuconostoc*	2057	54,2	GS			
201	A0A088FXI5	*Leuconostoc citreum* ABK1	*Bacillota*	*Lactobacillaceae*	*Leuconostoc*	2057	54,1	GS*	LmALT		47
202	Q9RE05	*Leuconostoc mesenteroides* NRRL B-1355	*Bacillota*	*Lactobacillaceae*	*Leuconostoc*	2057	54,4	GS*	ASR	6HVG	48, 49
203	WP_219053758.1	*Lactobacillus* Sy-1	*Bacillota*	*Lactobacillaceae*	*Lactobacillus*	1331	50,6	GS			
204	WP_249510840.1	*Apilactobacillus apisilvae*	*Bacillota*	*Lactobacillaceae*	*Lactobacillus*	874	46,0	GS			
205	WP_140934634.1	*Apilactobacillus micheneri*	*Bacillota*	*Lactobacillaceae*	*Lactobacillus*	862	44,0	BrS			
206	WP_053791619.1	*Apilactobacillus kunkeei*	*Bacillota*	*Lactobacillaceae*	*Lactobacillus*	1032	44,9	BrS			
207	WP_260148484.1	*Leuconostoc citreum*	*Bacillota*	*Lactobacillaceae*	*Leuconostoc*	1279	44,3	GS			
208	CDX66641	*Leuconostoc citreum* NRRL B-1299	*Bacillota*	*Lactobacillaceae*	*Leuconostoc*	1278	44,0	GS *	Dsr-DP		43
209	ASA47862.1	*Limosilactobacillus fermentum* NCC 3057	*Bacillota*	*Lactobacillaceae*	*Lactobacillus*	1047	41,7	GtfB *	GtfB	7DT1	50
210	WP_182578963.1	*Limosilactobacillus agrestis*	*Bacillota*	*Lactobacillaceae*	*Lactobacillus*	1282	40,5	GtfB			
211	WP_235958398.1	*Limosilactobacillus frumenti*	*Bacillota*	*Lactobacillaceae*	*Lactobacillus*	1211	41,5	GtfB			

212	WP_225362263.1	*Fructilactobacillus sanfranciscensis*	*Bacillota*	*Lactobacillaceae*	*Lactobacillus*	1151	39,4	GtfB		
213	WP_180993253.1	*Fructilactobacillus sanfranciscensis*	*Bacillota*	*Lactobacillaceae*	*Lactobacillus*	1282	39,2	GtfB		
214	WP_191667476.1	*Lactobacillus* M31	*Bacillota*	*Lactobacillaceae*	*Lactobacillus*	1488	40,9	GtfB		
215	ABQ83597.1	*Limosilactobacillus reuteri* DSM 20016	*Bacillota*	*Lactobacillaceae*	*Lactobacillus*	1363	39,9	GtfB *	GtfW		51
216	WP_267202213.1	*Limosilactobacillus* YH-lim2214	*Bacillota*	*Lactobacillaceae*	*Lactobacillus*	1364	40,3	GtfB		
217	WP_255135256.1	*Streptococcus thermophilus*	*Bacillota*	*Streptococcaceae*	*Streptococcus*	1603	41,2	GtfB		
218	WP_225437518.1	*Limosilactobacillus reuteri*	*Bacillota*	*Lactobacillaceae*	*Lactobacillus*	1590	40,9	GtfB		
219	A0A1Z2RUH3	*Limosilactobacillus reuteri* NCC 2613	*Bacillota*	*Lactobacillaceae*	*Lactobacillus*	1244	40,9	GtfB *	Lr2613 GtfB	7P38	52, 53
220	A0A1D7ZV76	*Limosilactobacillus fermentum* NCC 2970	*Bacillota*	*Lactobacillaceae*	*Lactobacillus*	1593	41,0	GtfB *	Lr2970 GtfB		54
221	WP_154548524.1	*Lactobacillus porci*	*Bacillota*	*Lactobacillaceae*	*Lactobacillus*	1373	40,7	GtfB		
222	WP_229305506.1	*Limosilactobacillus reuteri*	*Bacillota*	*Lactobacillaceae*	*Lactobacillus*	1620	40,4	GtfB		
223	AAU08003.2	*Limosilactobacillus reuteri* ML1	*Bacillota*	*Lactobacillaceae*	*Lactobacillus*	1620	40,7	GtfB *	GtfB ML4		51
224	AAU08014.2	*Limosilactobacillus reuteri* 121	*Bacillota*	*Lactobacillaceae*	*Lactobacillus*	1619	40,7	GtfB *	Lr121 GtfB	5JBD	55, 56
225	WP_229723701.1	*Limosilactobacillus caviae*	*Bacillota*	*Lactobacillaceae*	*Lactobacillus*	937	42,1	GtfB		
226	WP_261313217.1	*Limosilactobacillus panis*	*Bacillota*	*Lactobacillaceae*	*Lactobacillus*	1591	40,4	GtfB		
227	WP_064225342.1	*Ligilactobacillus aviarius*	*Bacillota*	*Lactobacillaceae*	*Lactobacillus*	1702	43,2	GtfB		
228	WP_081035579.1	*Ligilactobacillus araffinosus*	*Bacillota*	*Lactobacillaceae*	*Lactobacillus*	1515	43,3	GtfB		
229	WP_064213163.1	*Ligilactobacillus aviarius*	*Bacillota*	*Lactobacillaceae*	*Lactobacillus*	1567	43,0	GtfB		
230	WP_079579463.1	*Ligilactobacillus acidipiscis*	*Bacillota*	*Lactobacillaceae*	*Lactobacillus*	1493	39,9	GtfB		
231	WP_124978270.1	*Ligilactobacillus salitolerans*	*Bacillota*	*Lactobacillaceae*	*Lactobacillus*	1572	40,8	GtfB		
232	WP_212780512.1	*Lactobacillus corticis*	*Bacillota*	*Lactobacillaceae*	*Lactobacillus*	881	39,3	GtfB		
233	WP_213533855.1	*Lactococcus nasutitermitis*	*Bacillota*	*Streptococcaceae*	*Lactococcus*	1159	39,1	GtfB		
234	WP_159723789.1	*Enterococcus sp* CSURQ0835	*Bacillota*	*Enterococcaceae*	*Enterococcus*	1039	37,3	GtfB		
235	AKM18207.1	*Geobacillus* 12AMOR1	*Bacillota*	*Bacillaceae*	*Geobacillus*	903	34,6	GtfC *	GbGtfC	7ZC0	57, 58
236	WP_066235061.1	*Heyndrickxia sporothermodurans*	*Bacillota*	*Bacillaceae*	*Heyndrickxia*	902	35,3	GtfC		
237	WP_235601145.1	*Weizmannia coagulans*	*Bacillota*	*Bacillaceae*	*Weissella*	889	34,0	GtfC		
238	WP_035322188.1	*Peribacillus kribbensis*	*Bacillota*	*Bacillaceae*	*Peribacillus*	904	34,0	GtfC		
239	WP_214892803.1	*Exiguobacterium* H66	*Bacillota*	*Bacillaceae*	*Exiguobacterium*	893	32,4	GtfC		
240	WP_209548227.1	*Exiguobacterium multisp*	*Bacillota*	*Bacillaceae*	*Exiguobacterium*	893	31,7	GtfC		
241	ACB62096.1	*Exiguobacterium sibiricum* 255-15	*Bacillota*	*Bacillaceae*	*Exiguobacterium*	893	32,4	GtfC *	EsGtfC		59
242	WP_214771614.1	*Exiguobacterium multisp*	*Bacillota*	*Bacillaceae*	*Exiguobacterium*	892	31,8	GtfC		
243	WP_064299263.1	*Exiguobacterium* KKBO11	*Bacillota*	*Bacillaceae*	*Exiguobacterium*	892	32,4	GtfC		

244	WP_239984758.1	*Sporolactobacillus pectinivorans*	*Bacillota*	*Sporolactobacillaceae*	*Sporolactobacillus*	1022	35,4	GtfC		
245	WP_214713558.1	*Bacillus* ISL-46	*Bacillota*	*Bacillaceae*	*Bacillus*	783	32,2	GtfD		
246	WP_052702730.1	*Paenibacillus beijingensis* DSM 24997	*Bacillota*	*Paenibacillaceae*	*Paenibacillus*	776	34,8	GtfD *	PbGtfD	60
247	WP_224722266.1	*Paenibacillus vietnamensis*	*Bacillota*	*Paenibacillaceae*	*Paenibacillus*	777	33,1	GtfD		
248	WP_223067679.1	*Paenibacillus caui*	*Bacillota*	*Paenibacillaceae*	*Paenibacillus*	777	34,1	GtfD		
249	WP_175890816.1	*Burkholderia multisp*	*Pseudomonadota*	*Burkholderiaceae*	*Burkholderia*	721	33,8	GtfD		
250	WP_175911446.1	*Burkholderia multisp*	*Pseudomonadota*	*Burkholderiaceae*	*Burkholderia*	727	32,5	GtfD		
251	NIJ05635.1	*Frigoribacterium faeni*	*Actinomycetota*	*Microbacteriaceae*	*Frigobacterium*	923	32,0	GtfD		
252	RJG09097.1	*Pseudomonas cavernicola*	*Pseudomonadota*	*Pseudomonadaceae*	*Pseudomonas*	797	32,3	GtfD		
253	NVK44519.1	*Oceanospirillaceae bacterium*	*Pseudomonadota*	*Oceanospirillaceae*	*Oceanospirillaceae*	775	31,4	GtfD		
254	WP_167520052.1	*Azotobacter salinestris*	*Pseudomonadota*	*Pseudomonadaceae*	*Azotobacter*	742	30,8	GtfD		
255	AJE22990.1	*Azotobacter chroococcum* NCIMB 8003	*Pseudomonadota*	*Pseudomonadaceae*	*Azotobacter*	780	30,7	GtfD *	AcGtfD	61
256	WP_049623289.1	*Frateuria defendens*	*Pseudomonadota*	*Rhodanobacteraceae*	*Frateuria*	790	30,0	GtfD		
257	WP_142092843.1	*Propioniferax innocua*	*Actinomycetota*	*Propionibacteriaceae*	*Propioniferax*	1011	32,5	GtfD		
258	WP_172421791.1	*Enemella evansiae*	*Actinomycetota*	*Propionibacteriaceae*	*Enemella*	1017	31,8	GtfD		
259	NNG18745.1	*Naumannella* ID2617S	*Actinomycetota*	*Propionibacteriaceae*	*Naumanella*	1016	32,1	GtfD		

7

Figure S1

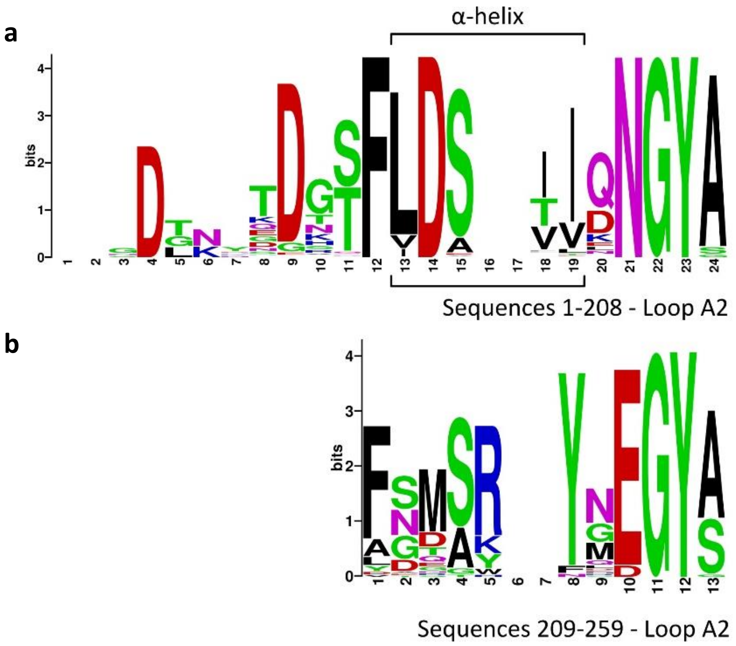

Figure S1. Sequence logos of loop A2. (a) Residues belonging to loop A2 in the first 208 representative GH70 sequences, and (b) residues belonging to loop A2 in the last 51 representative GH70 sequences, showing a clear distinction in length and composition between putative GS/BrS enzymes and α-GT enzymes. Unlike α-GTs, Gs/BrS enzymes feature a helical segment that blocks donor subsites beyond -1. The gaps occur due to a few sequences with a longer loop A2.

Figure S2 – S10

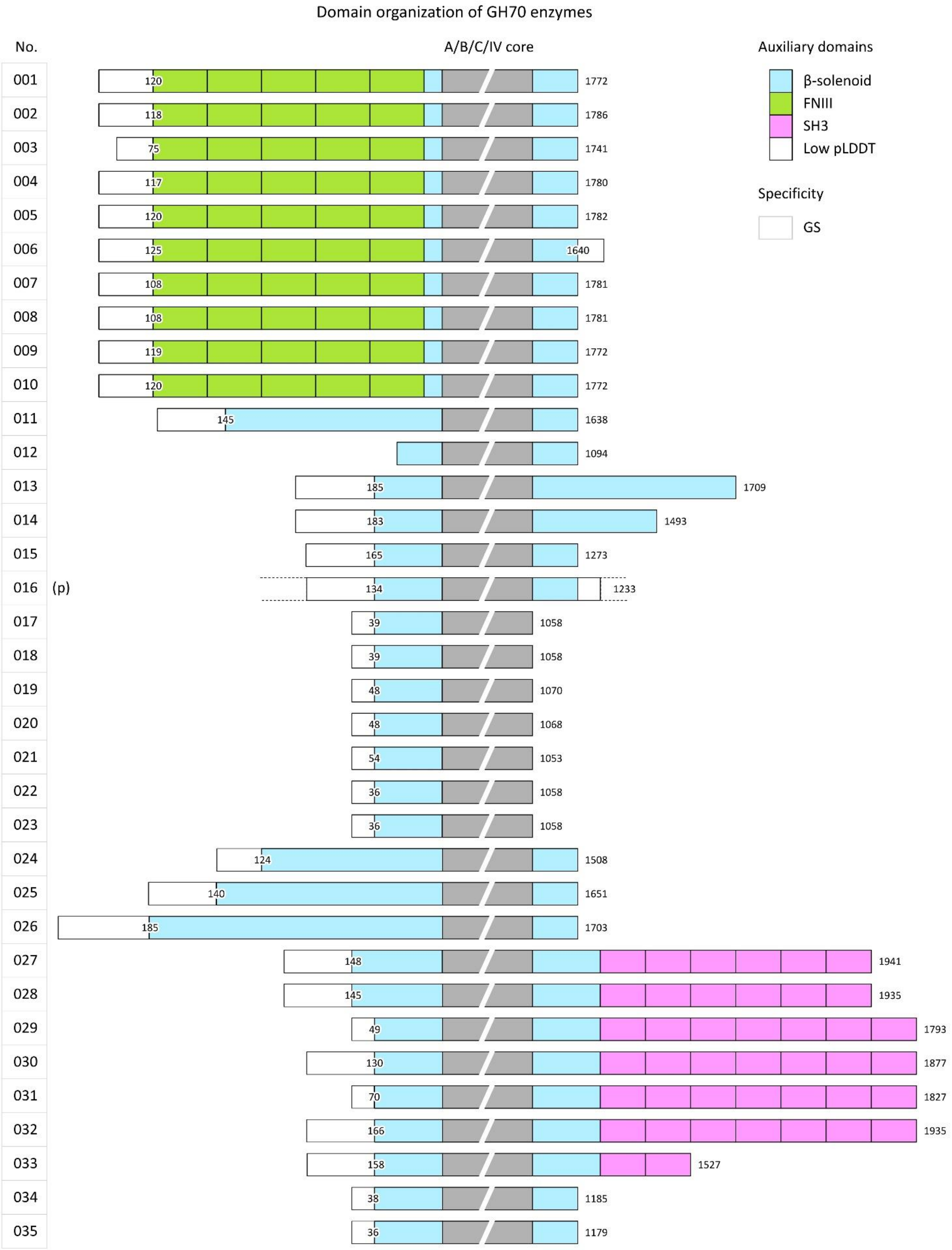

Figure S2 (continued)

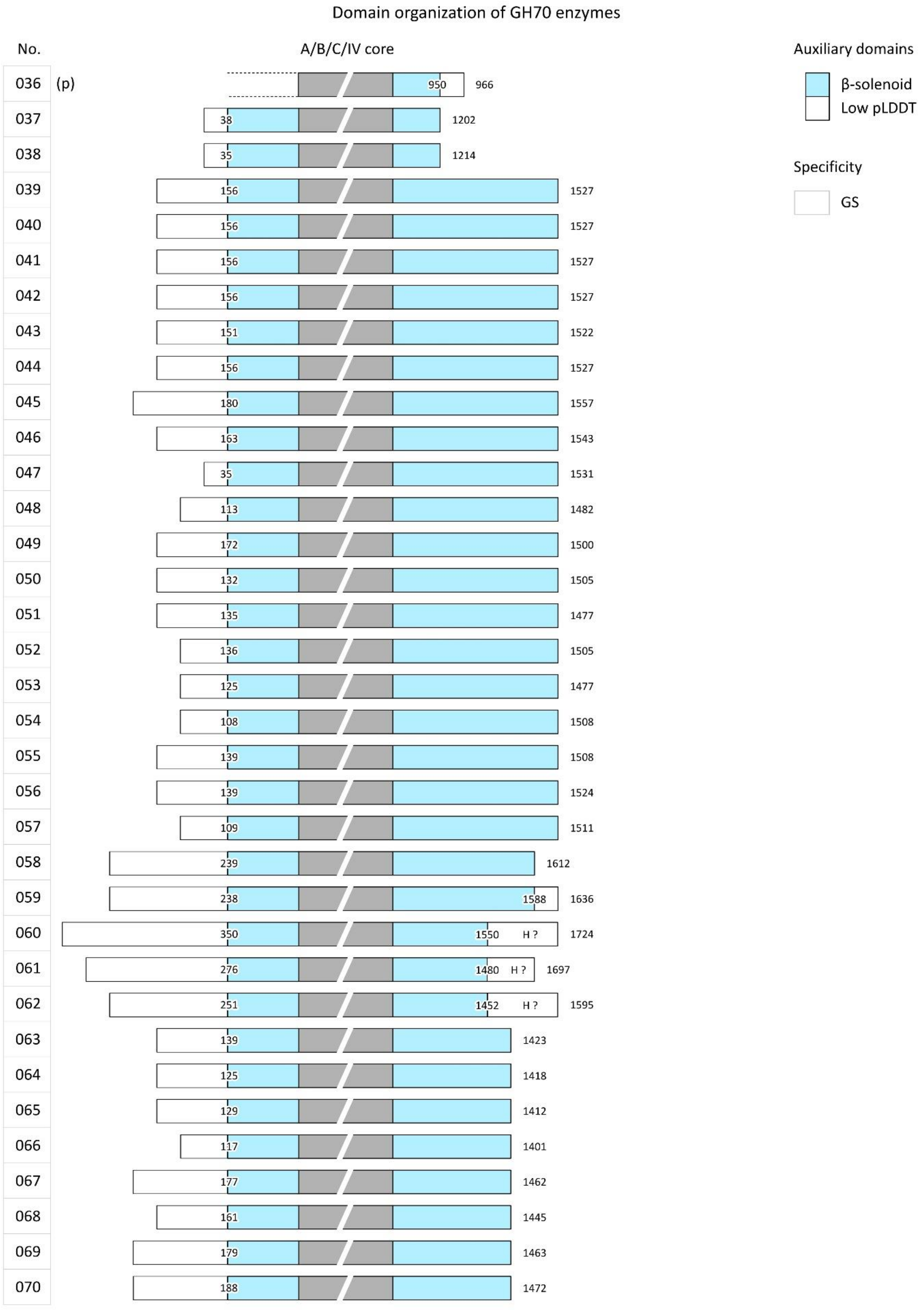

Figure S2 (continued)

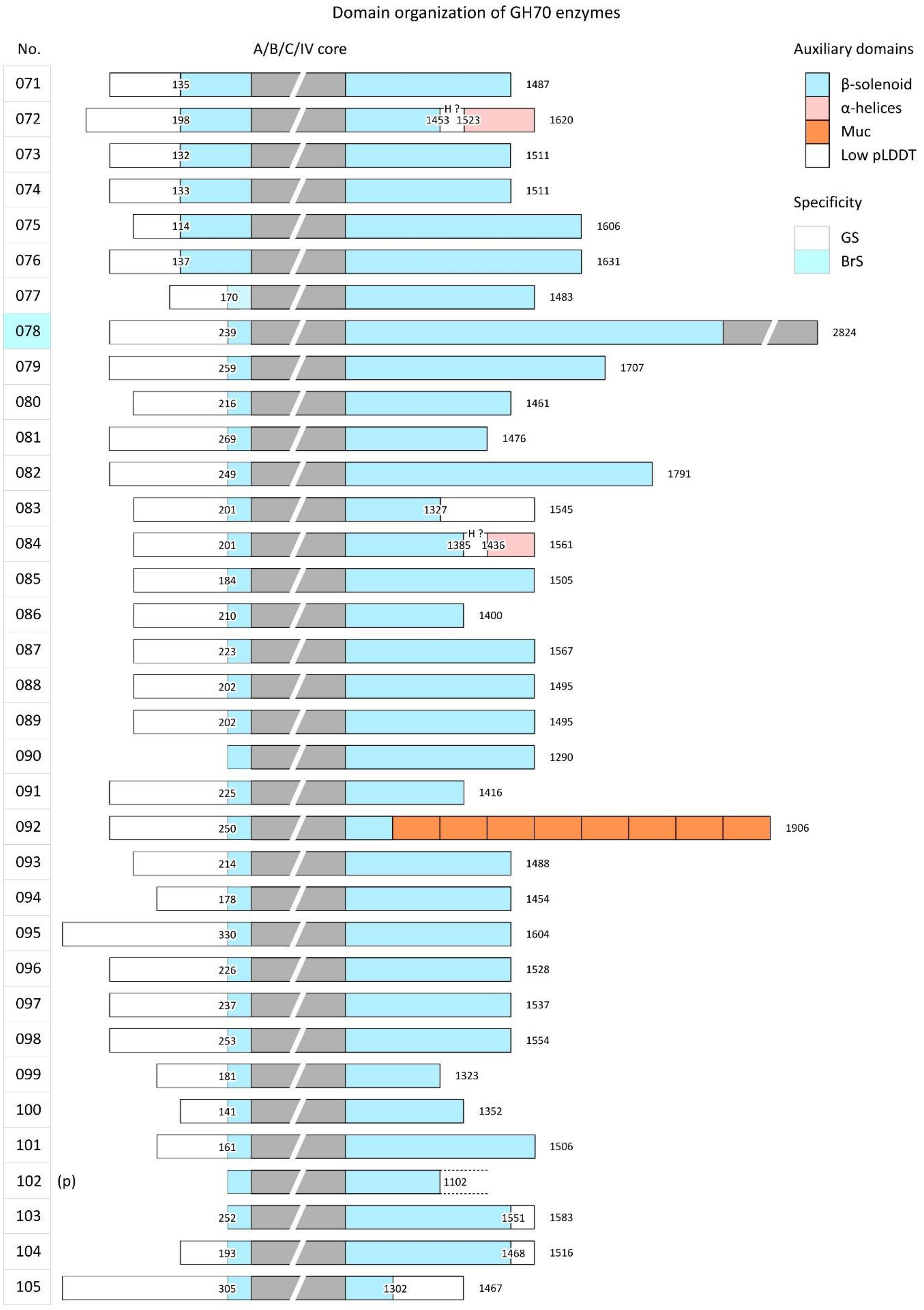

Figure S2 (continued)

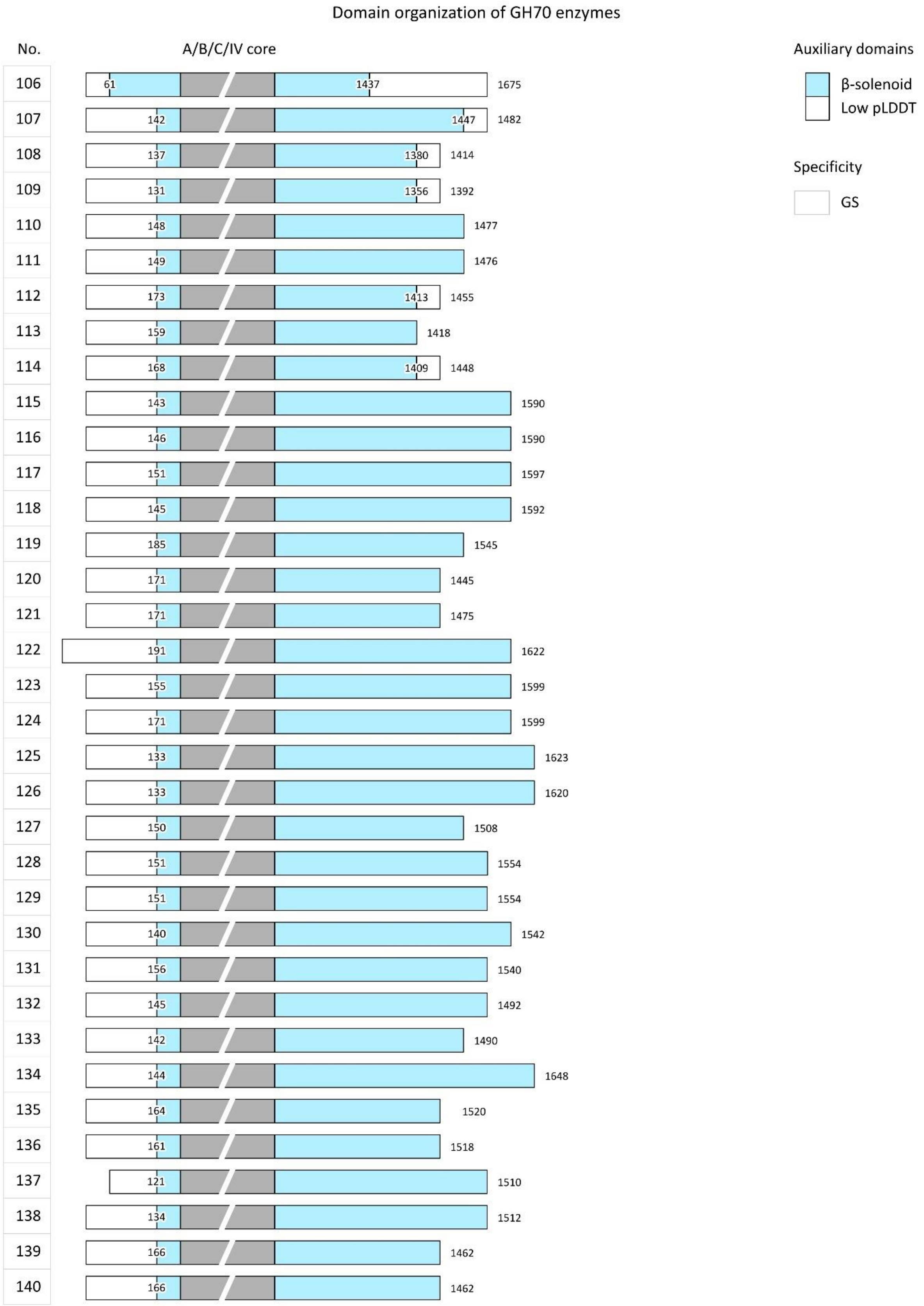

Figure S2 (continued)

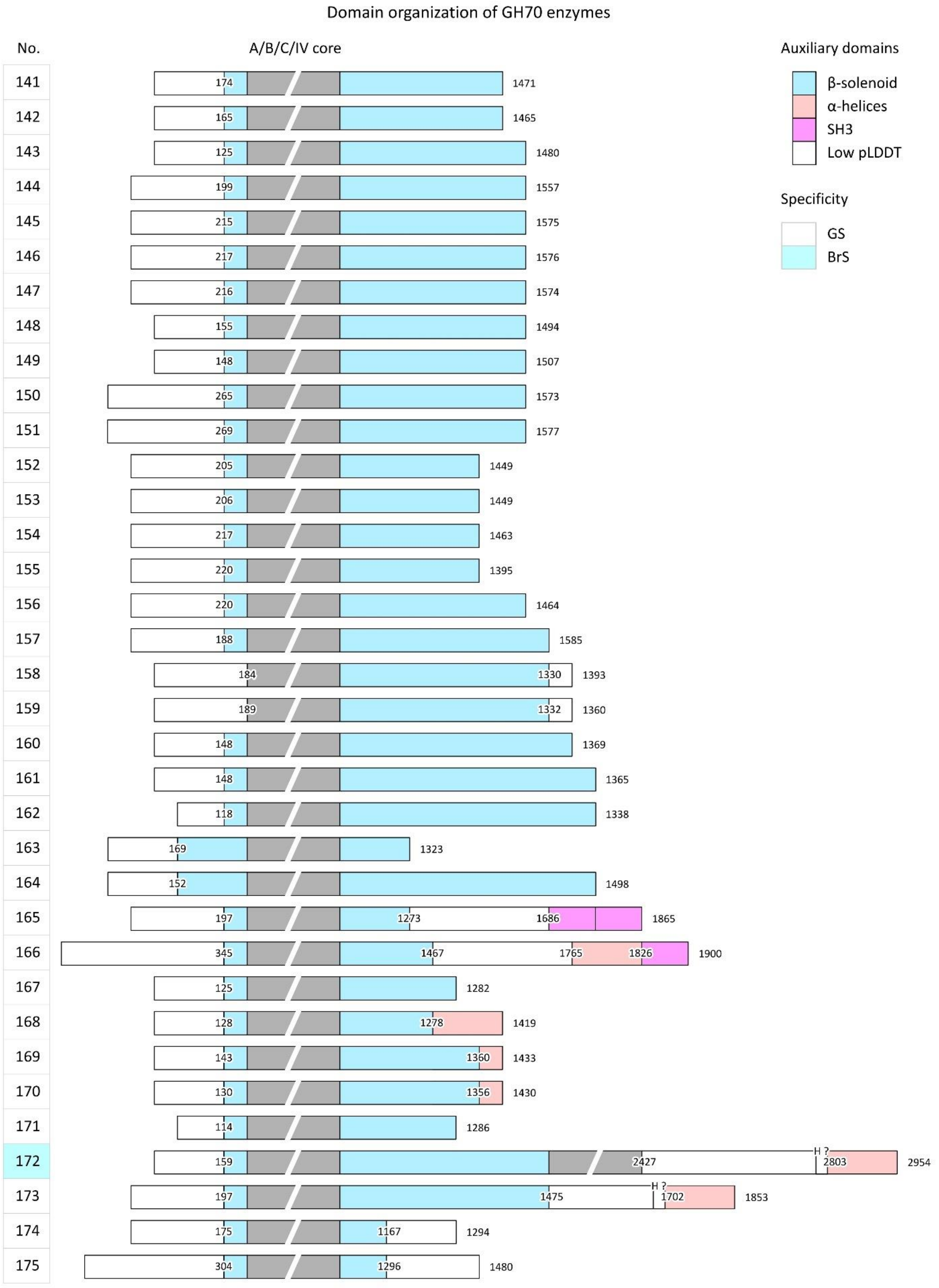

Figure S2 (continued)

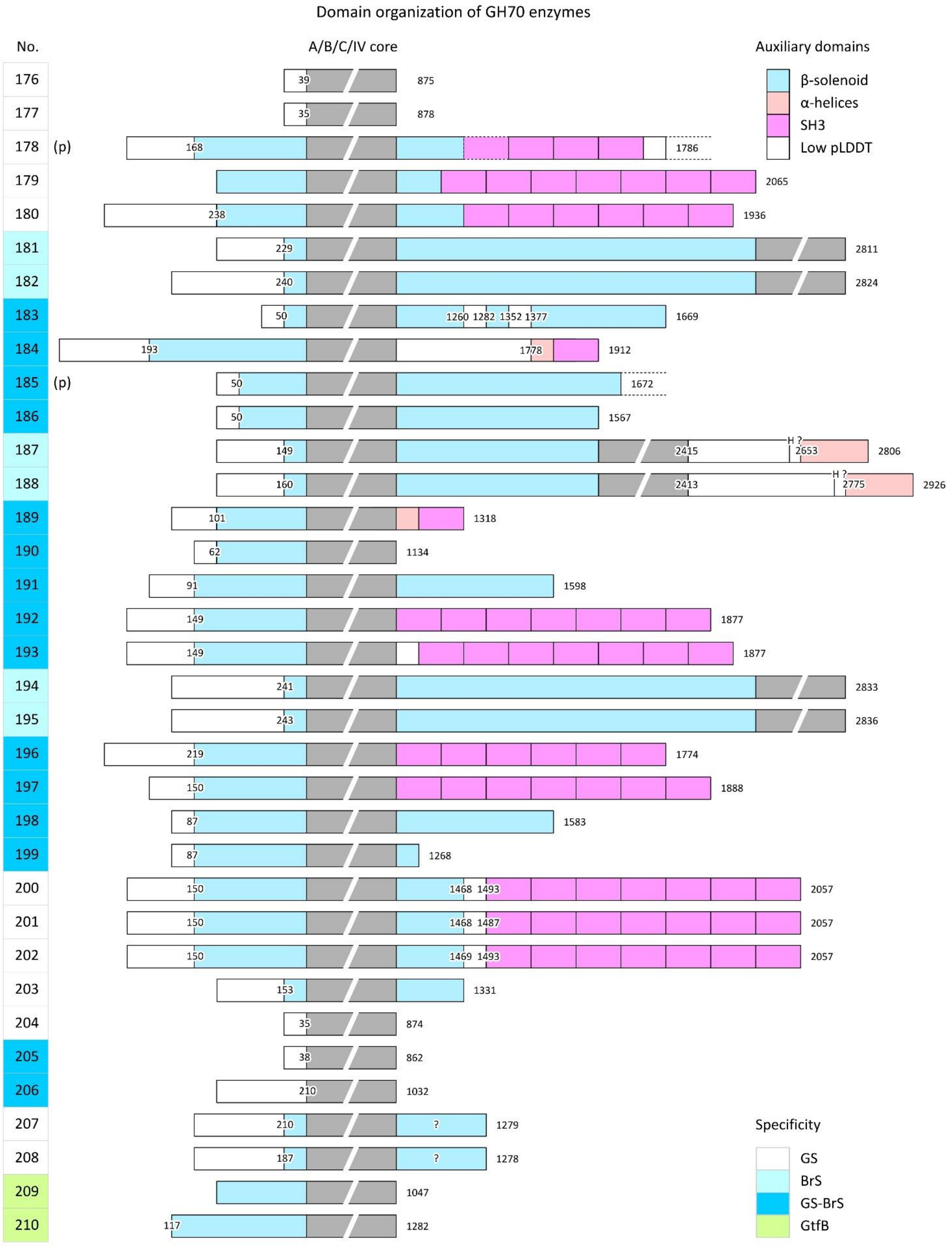

Figure S2 (continued)

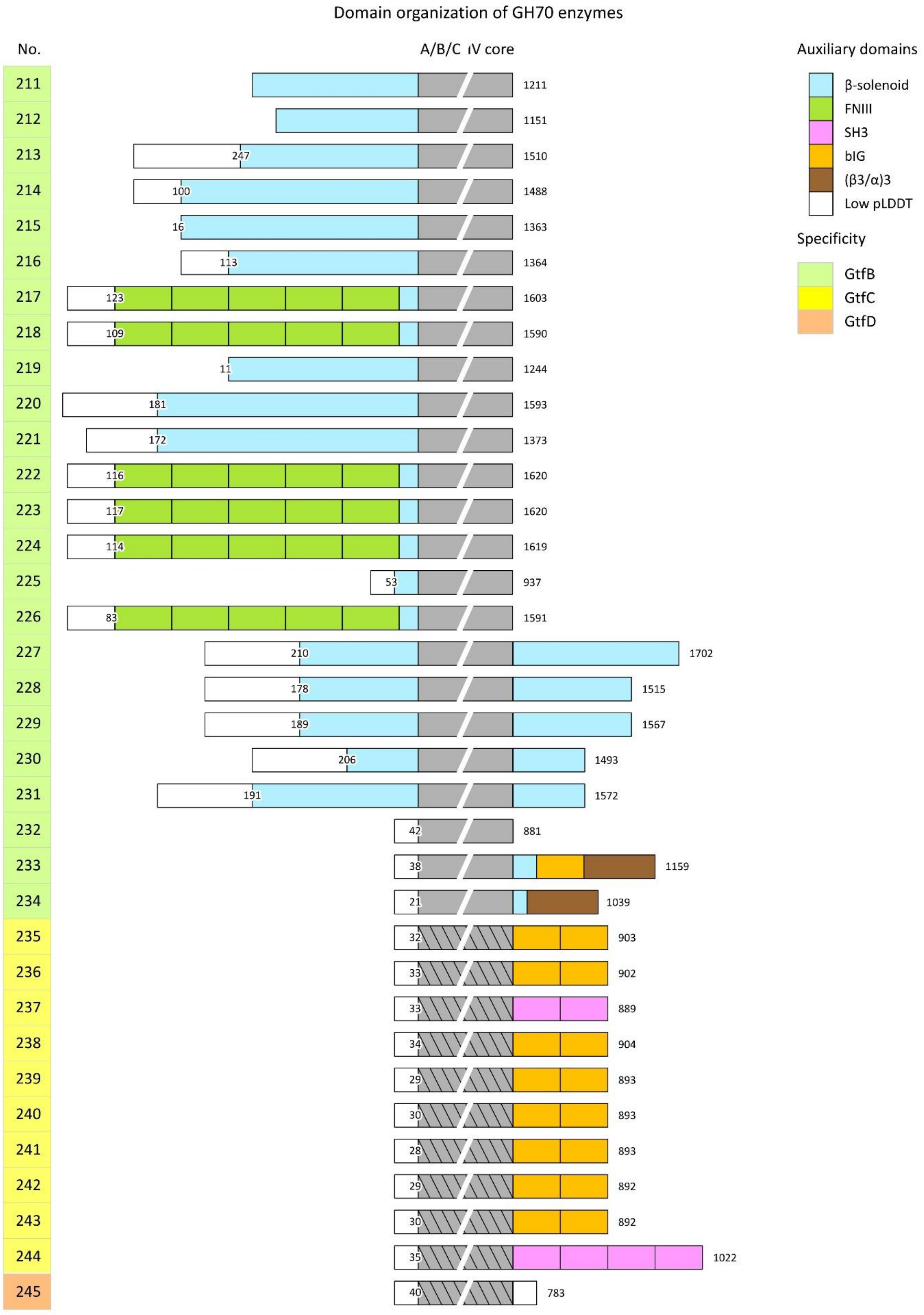

Figure S2 (continued)

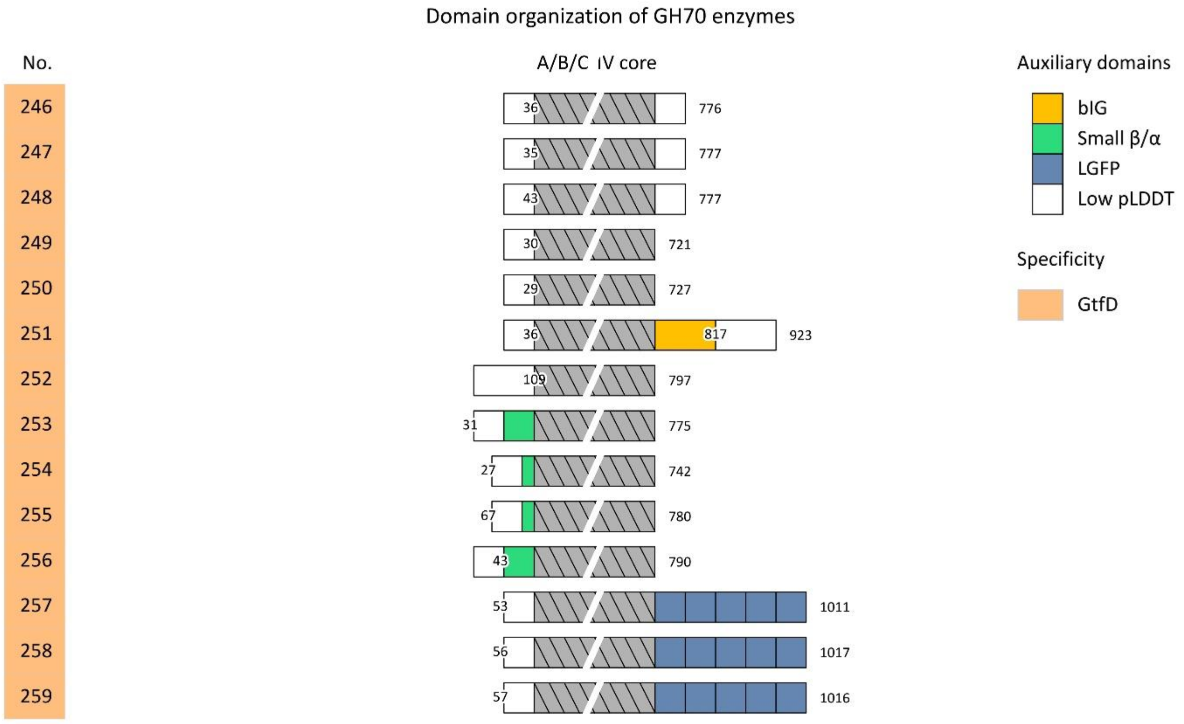

Figure S2. Domain organization of 259 representative GH70 sequences deducted from AlphaFold models. The sequences are aligned on the (first) A/B/C/IV core, shown in grey; hatching indicates the non-permuted cores. Color coding on the sequence numbers corresponds to the predicted enzyme specificities. Auxiliary domains are colored according to their type; the length of the blocks roughly corresponds to the length in the sequence. White boxes indicate the (mostly N- and C-terminal) segments longer than about 10 residues for which AlphaFold modeling gave pLDDT scores <60. Numbers in the sequences indicate approximate domain boundaries and C-termini. Dotted outlines indicate that some sequences were annotated as partial (N- and/or C-terminal) in GenBank (also indicated with “(p)”).

Figure S3.

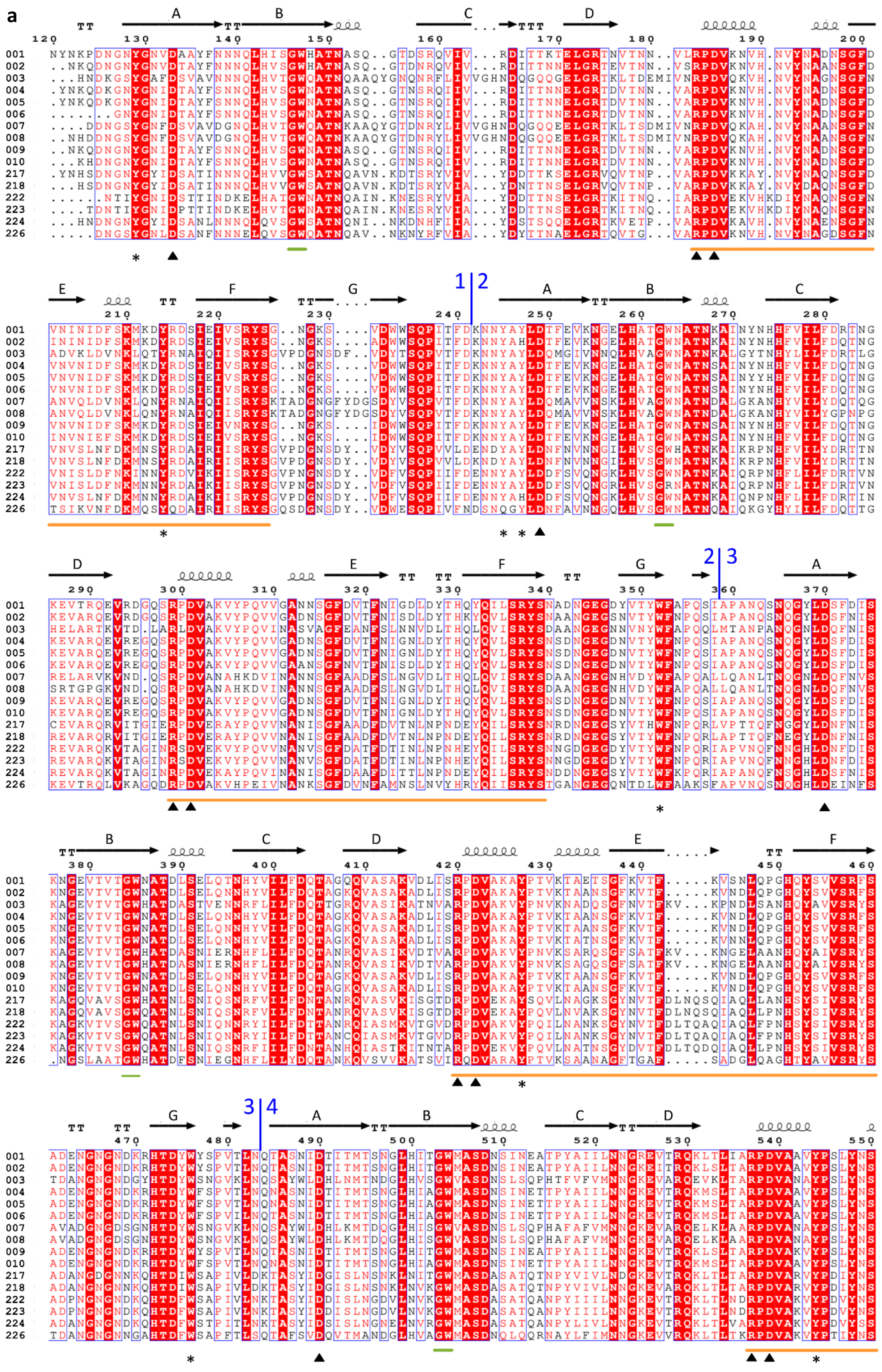

Figure S3 (continued)

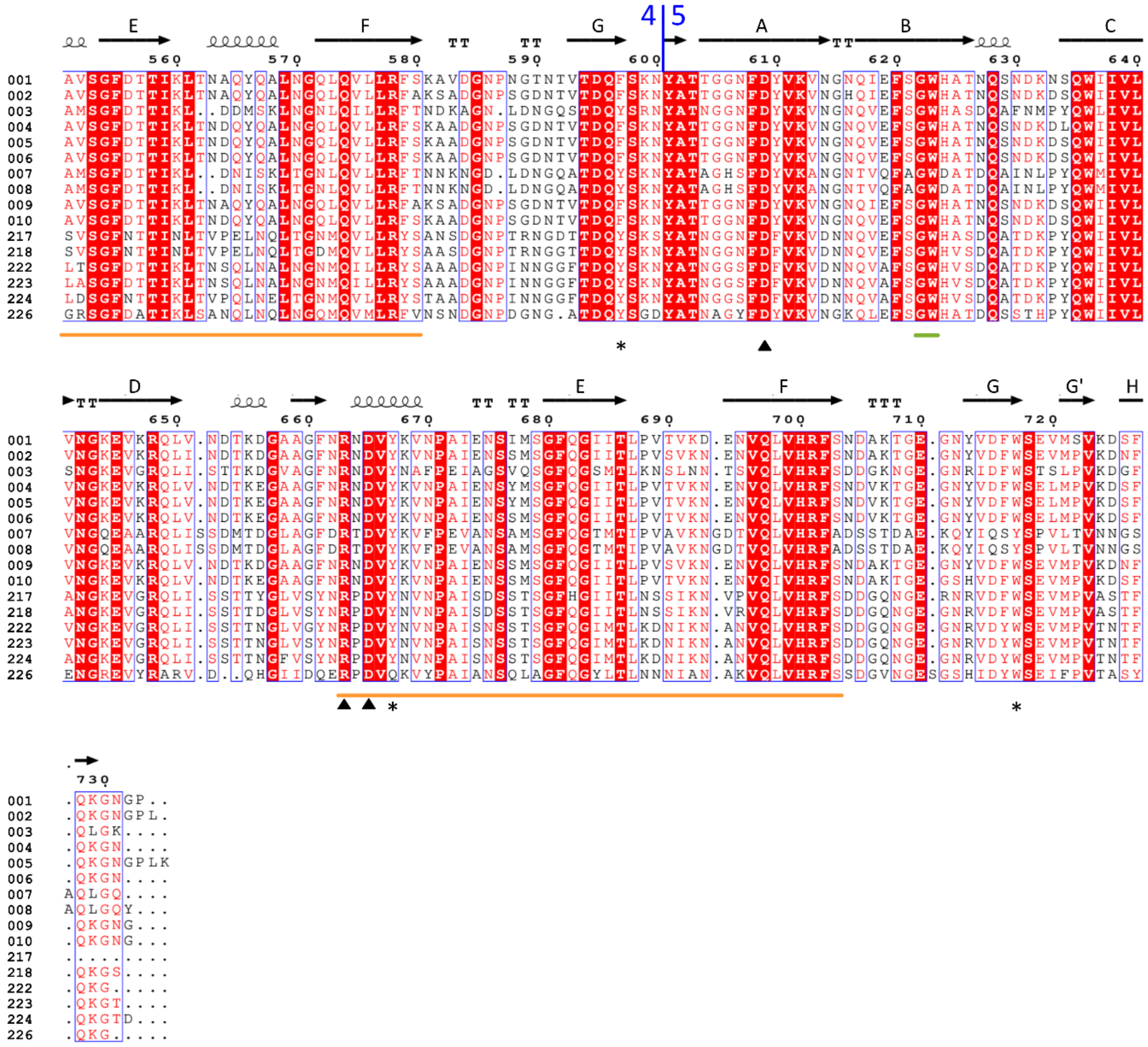

b

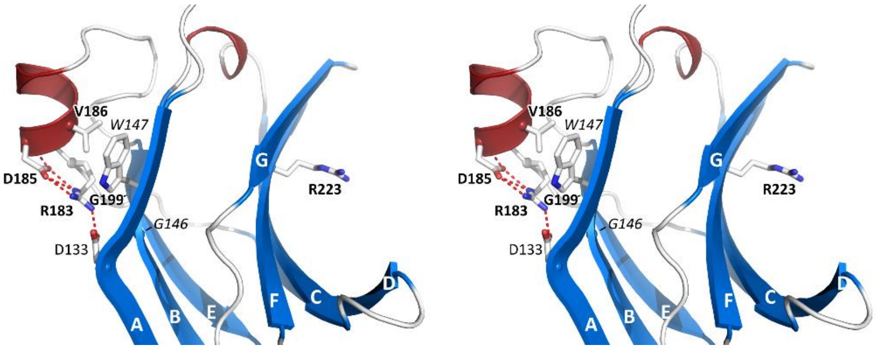

Figure S3. FNIII auxiliary domains in GH70. (a) Sequence alignment of the FNIII domains in 16 GH70 enzymes (entry numbers are given at the beginning of each line and correspond to those in Table S1). Secondary structure elements and residue numbers are those of entry 001 (*L. reuteri* 180 glucansucrase Gtf180); the β-strands are labeled. Vertical blue lines indicate the approximate borders of the five FNIII domains 1-5. In each domain, the conserved RDV motifs are indicated by the yellow sections, and the GW motifs by the green sections. The three residues (D, R, D) in each domain forming

salt bridge interactions are indicated by a black triangle, and the fully or almost fully conserved aromatic residues that are predicted at to lie at the protein surface by an asterisk (*). (b) Stereo view of a representative FNIII domain, the first FNIII domain (residues 125-242) in *L. reuteri* 180 glucansucrase Gtf180 (no. 001); the seven β-strands are labeled A-G. Fully conserved residues in the RDV-repeat (**R**(P/N/S/T/Q)**DV**-x_{11-12}-S/A**G**Y/F-x_{17-22}-**R**(Y/F)S) are shown with bold labels. Residues R183, D185 and D133 form a three-residue salt bridge (interactions are shown as red dotted lines); they cluster together with an almost fully conserved GW-motif (residues 146 and 147, shown with italics labels).

Figure S4

a

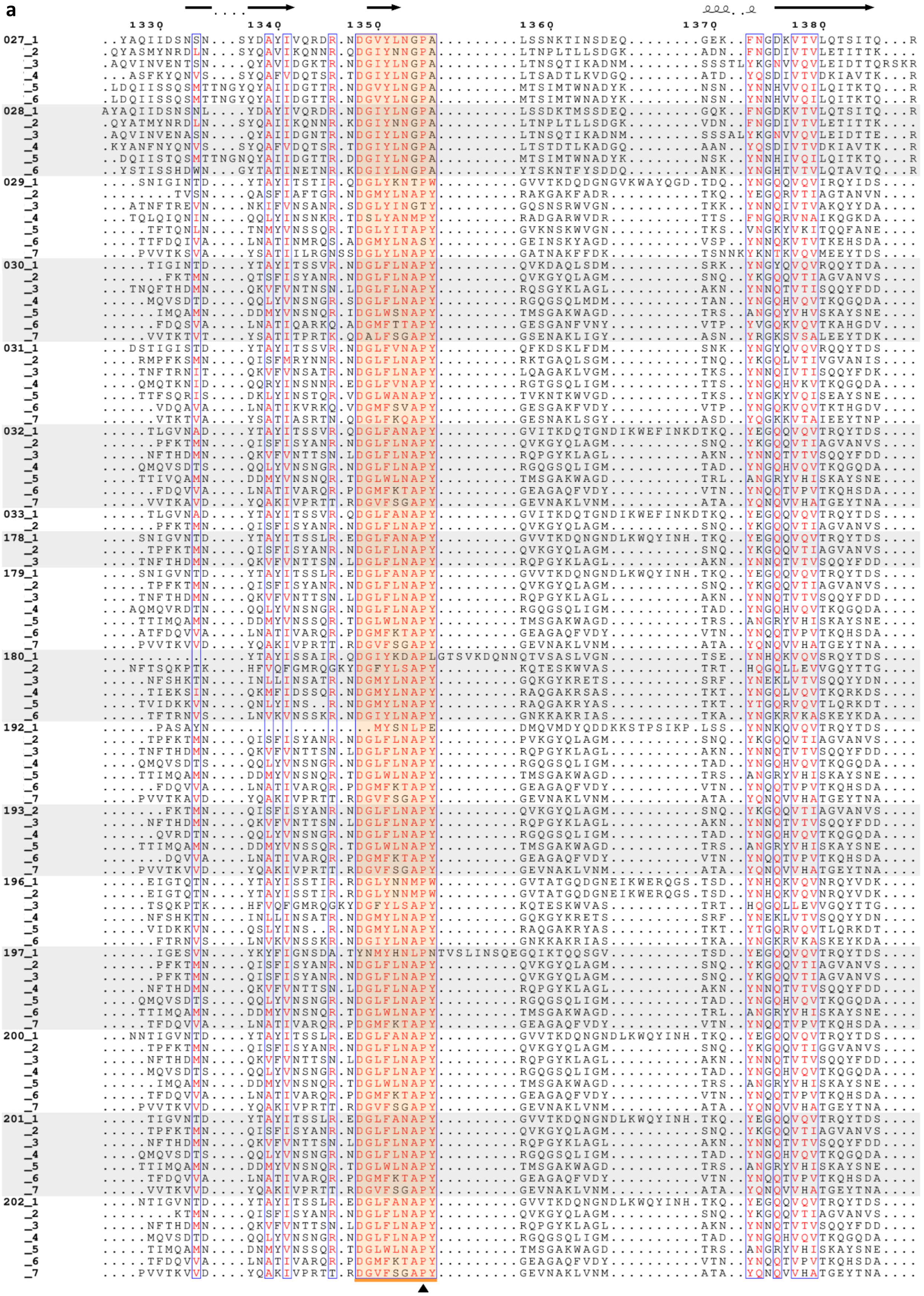

Figure S4 (continued)

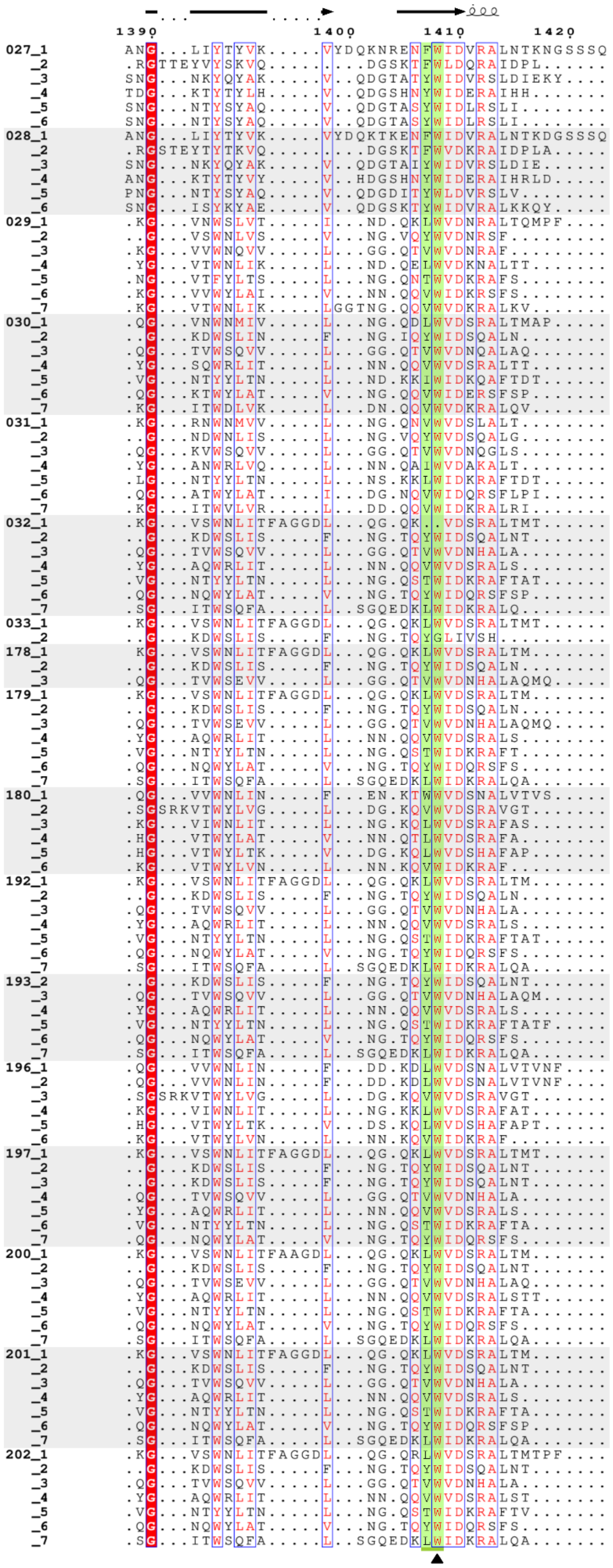

Figure S4 (continued)

b

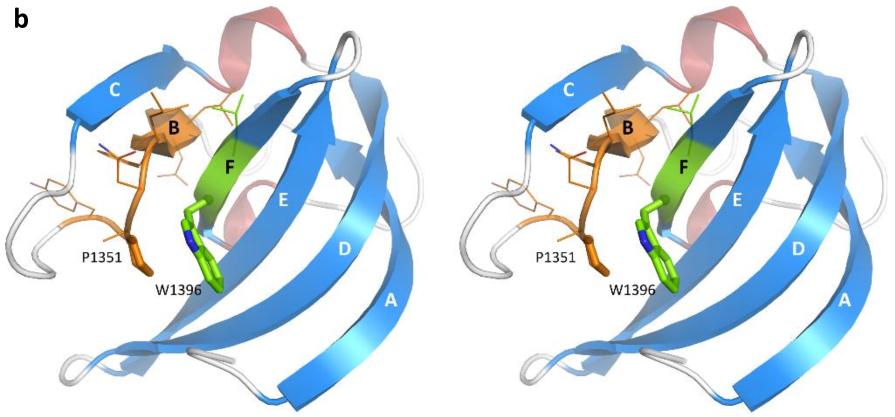

c

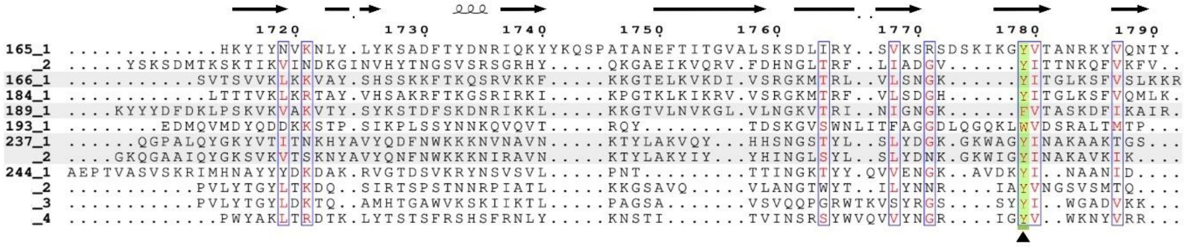

Figure S4. SH3 domains in GH70. (a) Sequence alignment of individual SH3_8 domains in GH70 enzymes ("Group 1"). Entry numbers are given at the beginning of each line and correspond to those in Table S1; the number after the underscore depicts the domain number in that entry. Secondary structure elements and residue numbers are those of the first SH3_8-domain of entry 030 (*Leuconostoc gelidum* glucansucrase). The APY motif is highlighted in orange; the xW motif is highlighted in green; the respective proline and tryptophan in this motif are indicated by a black triangle. (b) Stereo view of a representative SH3_8 domain, the first SH3 domain (residues 1326-1406) in *Leuconostoc gelidum* predicted glucansucrase (no. 030); the six β-strands are labeled A-F. The conserved APY-motif (DGLFLNAPY in this case) is highlighted in orange, and the semi-conserved xW motif (where x = aliphatic/aromatic) in green. From these motifs, the respective conserved proline and tryptophan are shown in side chain sticks showing their mutual stacking interaction. (c) Sequence alignment of the remaining SH3_3/DUF5576 domains in GH70 ("Group 2") lacking the APY-motif. Secondary structure elements and residue numbers are those of the second SH3_3 domain of entry no. 165 (*Lactobacillus* Sy-1 glucansucrase).

Figure S5

a

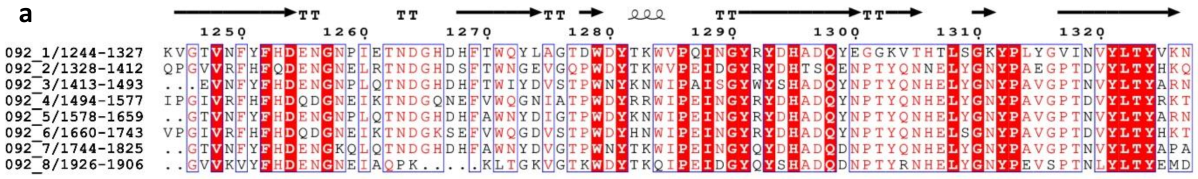

b

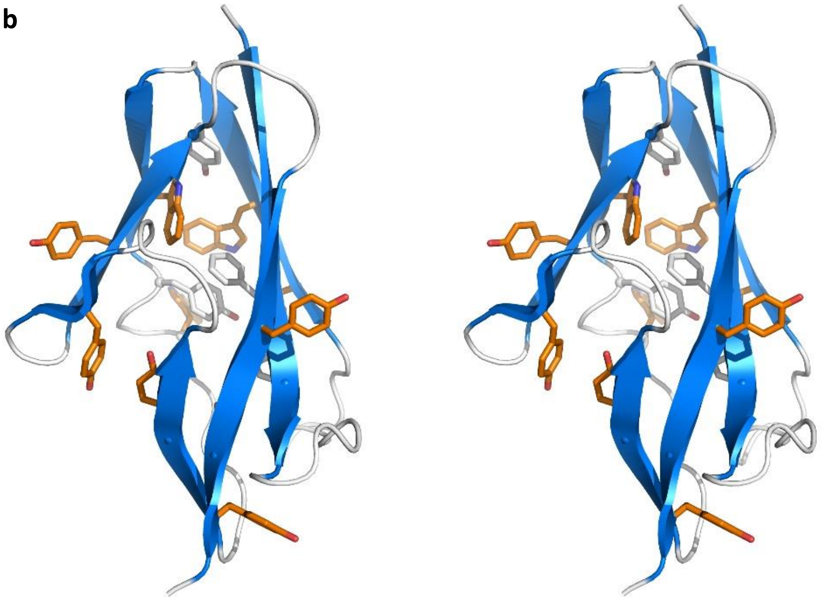

Figure S5. MucBP domains in GH70. (a) Sequence alignment of the 8 MucBP auxiliary domains in no. 092 (*Fructilactobacillus hinvesii* GS). Residue numbering and secondary structure corresponds to that of the first MucBP domain. (b) Stereo view of the second MucBP domain (residues 1328-1412). Aromatic residues are shown in stick representation; the more buried ones are colored in white, while the more exposed ones are colored in orange.

Figure S6

a

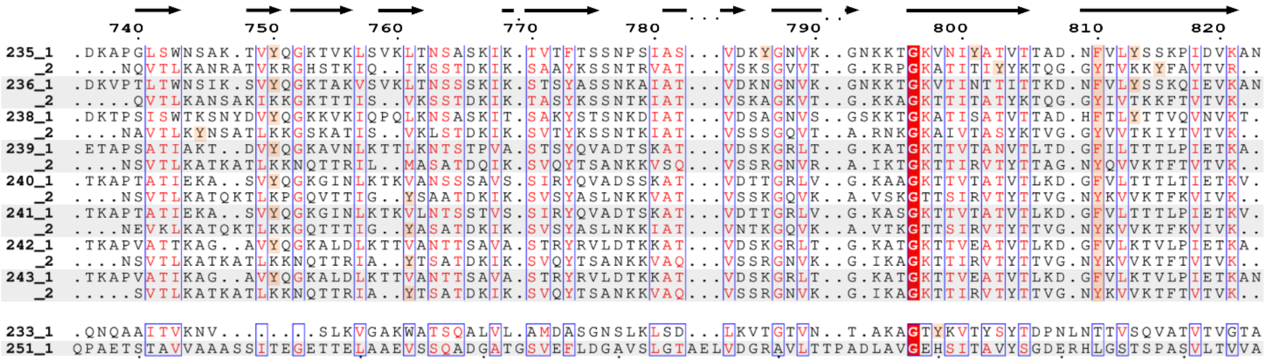

b

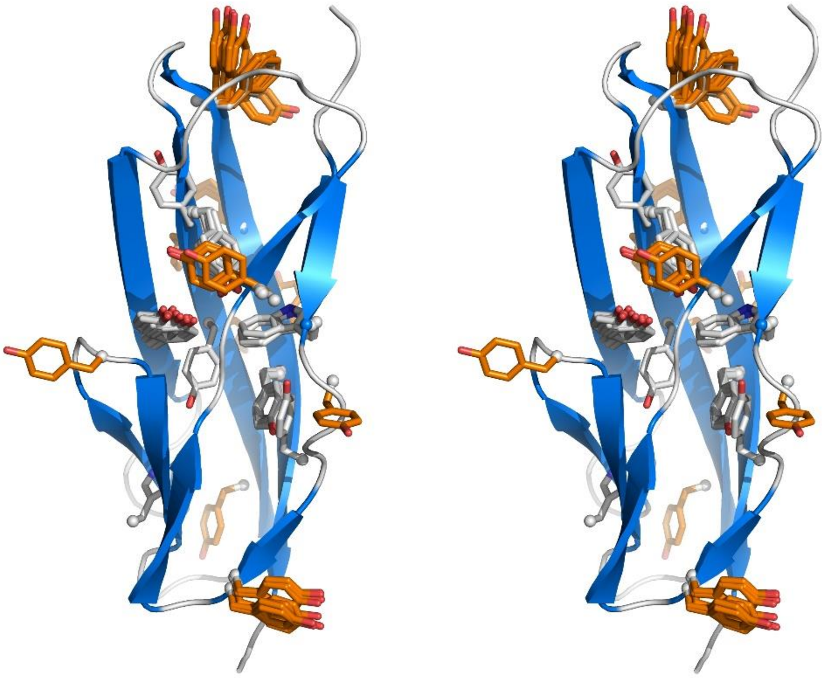

Figure S6. bIG domains in GH70. (a) Sequence alignment of individual bIG domains in 10 GH70 enzymes. The first eight entries depict the bIG_2 type domains; the last two align less well and likely resemble bIG_3 domains. Entry numbers are given at the beginning of each line and correspond to those in Table S1; the number after the underscore depicts the domain number in that entry. Secondary structure elements and residue numbers are those of the first bIG_2 domain of entry 235 (*Geobacillus* 12AMOR1 α-glucanotransferase GtfC). Aromatic residues that project near the surface are highlighted in orange. (b) Stereo view of superimposed bIG_2 domains, found in 10 GH70 entries. The cartoon representation is that of *Geobacillus* 12AMOR1 GtfC (no. 235). Aromatic residues from all 17 bIG_2 domains are shown in stick representation; the more buried ones are colored in white, while the more exposed ones are colored in orange.

a

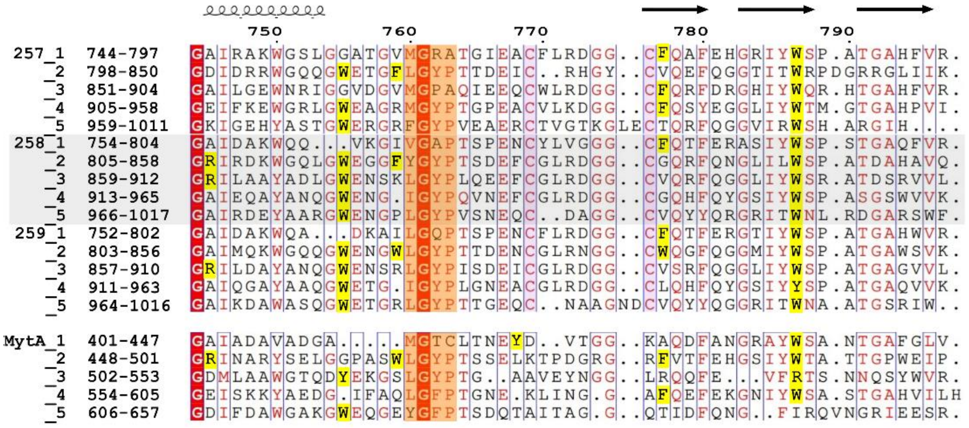

b

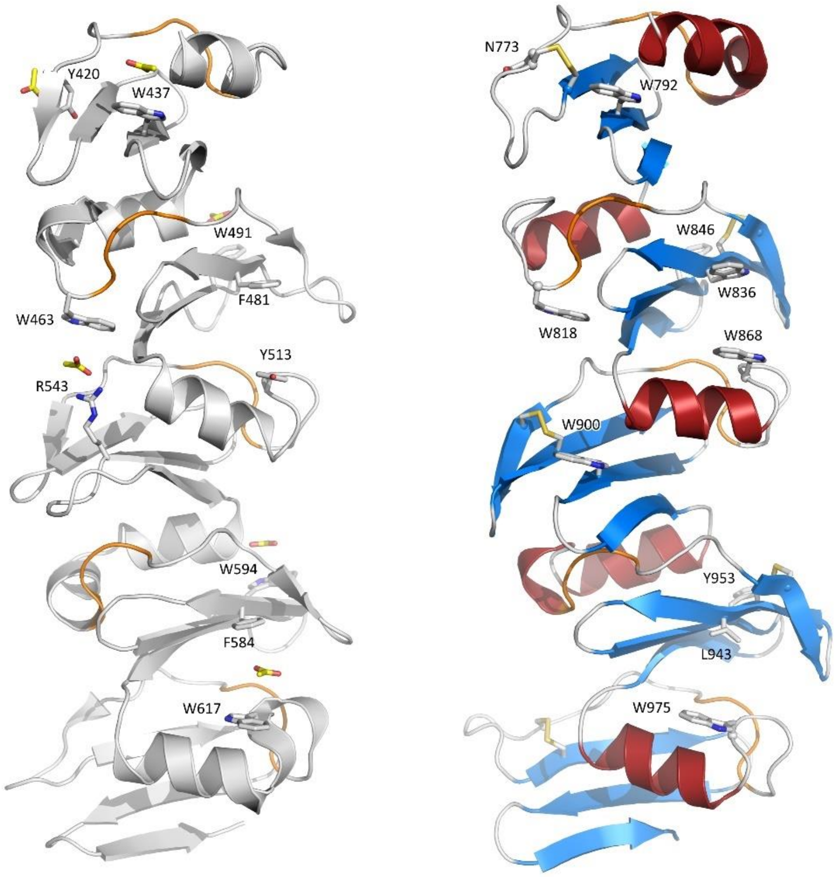

Figure S7. LGFP domains in GH70. (a) Sequence alignment of the five LGFP domains of GtfDs (entries 257-259) and *C. glutamicum* MytA. The LGFP motifs are shaded in orange; residues of MytA interacting with acetate ligands are highlighted in yellow, as are the corresponding (semi-)conserved residues in 257-259. The residue numbering and secondary structure elements are from the first LGFP domain of entry 257 (*Naumannella* sp ID2617S GtfD). Conserved cysteine residues predicted to form a disulfide bridge in the GH70 proteins are highlighted in purple. (b) Structural comparison of the five C-terminal LGFP domains of *C. glutamicum* MytA (left panel; PDB: 6SX4[62]); residues 401-657 of chain A) and *Naumannella* sp ID2617S GtfD (no. 259; AlphaFold model residues 752-1016). The five LGFP motifs of both proteins are highlighted in orange. In MytA, the bound acetate ligands, as well as

interacting residues are shown in stick representation (acetate in yellow). In the *Naumannella* GtfD, the corresponding residues, and the five predicted disulfide bridges, are also shown in stick representation.

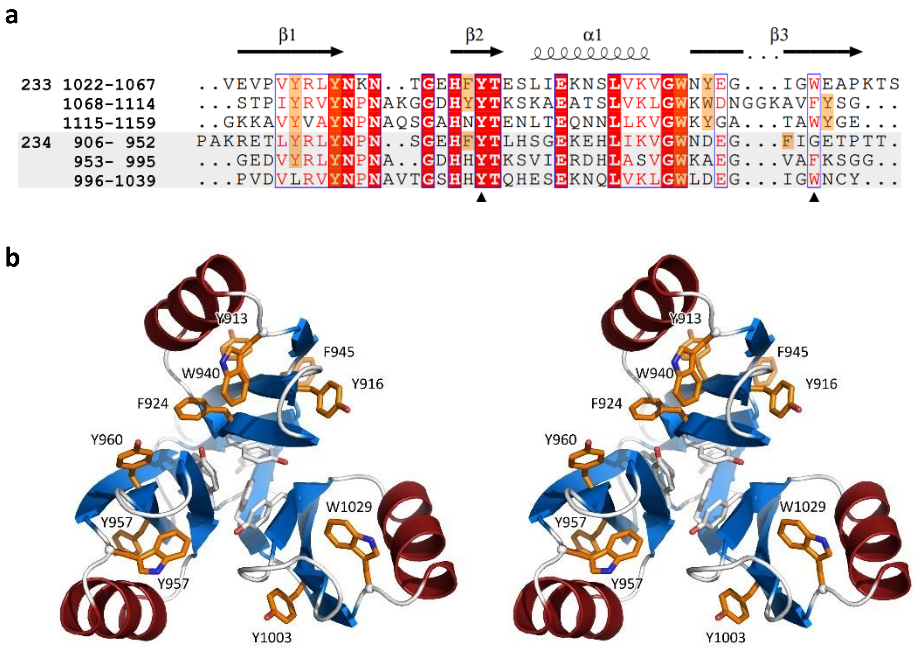

Figure S8. (β3α)3 domains in GH70. (a) Sequence alignment of the β3α subdomains in the (β3α)3 domains of no. 233 and 234; the secondary structure elements are derived from the first β3α subdomain of no. 234. The most exposed aromatic residues are highlighted in orange; those forming the central hydrophobic core are indicated by a black triangle. (b) Stereo view of the (β3α)3 domain of *Enterococcus* sp. CSURQ0835 (no. 234), looking down the approximate 3-fold axis. The most exposed aromatic residues are labeled and highlighted in orange.

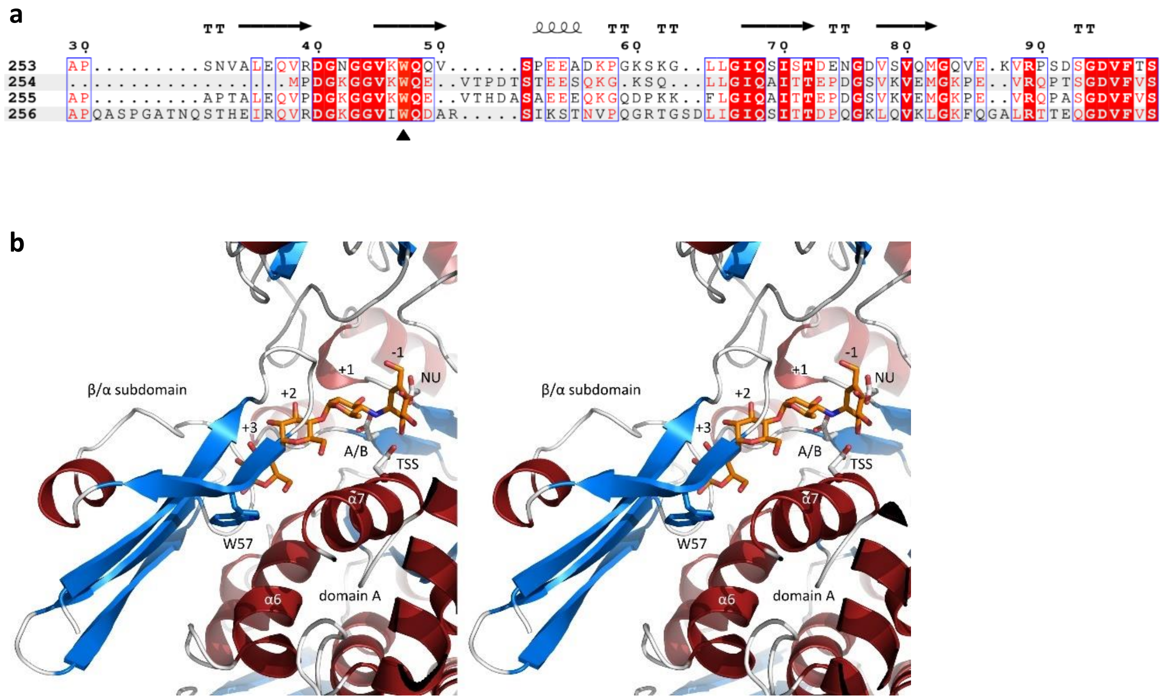

Figure S9. Small β/α domains in GH70. (a) Sequence alignment of the small β/α domains of entries no. 253-256 (GtfD-type α-GTs). Residue numbering and secondary structure elements are from no. 253 (*Oceanospirillaceae bacterium* GtfD). The conserved tryptophan residue in the second β-strand is indicated by a black triangle. (b) Stereo view of the small β/α domain of *Oceanospirillaceae bacterium* GtfD (no. 253) constituting a small β/α subdomain, located adjacent to helices α6 and α7 of the catalytic domain A. From a superposition with *L. reuteri* NCC 2613 GtfB complexed with acarbose (PDB: 7P39[53]), the bound pseudotetrasaccharide inhibitor in subsites +3 to -1 is shown as sticks with orange carbon atoms. This shows that the small β/α subdomain of the *O. bacterium* GtfD (absent in *L. reuteri* NCC 2613 GtfB) lies adjacent to acceptor subsites; its single aromatic residue W47 in the second β-strand of the β/α domain is highlighted. The *O. bacterium* GtfD active site is indicated with its three predicted catalytic residues, the nucleophile (NU), acid/base (A/B) and transition state stabilizing residue (TSS).

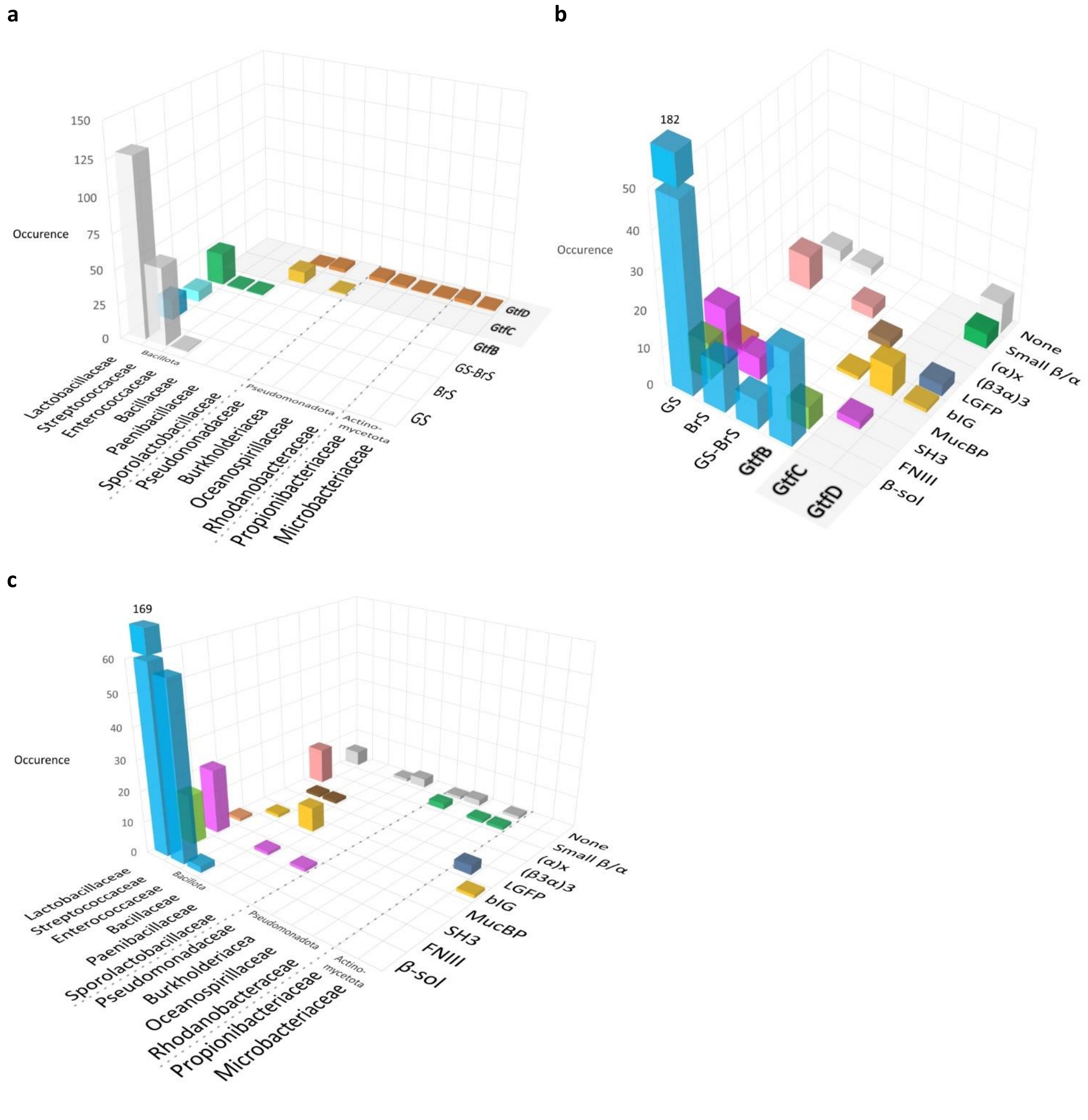

Figure S10. Occurrence/distribution of bacterial origin, enzyme specificity and auxiliary domain type in 259 GH70 enzymes. (a) Distribution of enzyme specificity over bacterial phyla and families. For each bacterial family, the occurrence of (predicted) specificities in the representative set of 259 enzymes is plotted on the vertical axis. Most specificities occur in a limited number of families; the GtfD specificity is found in most families and the only one found in all three phyla. The shaded area represents non-permuted enzymes, also showing a striking separation with permuted enzymes regarding the bacterial families in which they are found. Bold-labeled specificities represent α-glucanotransferases (α-GTs). (b) Occurrence of auxiliary domain type in different enzyme specificities. The shaded area represents non-permuted enzymes; bold-labeled specificities represent α-glucanotransferases (α-GTs).

Glucansucrases (GS) shows the largest diversity of auxiliary domain types. (c) Occurrence of auxiliary domain types in bacterial phyla and families containing GH70 enzymes. For each bacterial family, the occurrence of (predicted) auxiliary domain type in the representative set of 259 enzymes is plotted on the vertical axis. β-solenoid auxiliary domains are by far the most abundant. Some domain types occur only in a single bacterial family (e.g., FNIII, bIG, (α)x). Except for MucBP, which is found in two different phyla, all auxiliary domain types are restricted to one of the three bacterial phyla.

Table S2.

Year	First author	Topic	Ref.
1999	Monchois	Glucansucrases:mechanism, structure-function relationship	63
2003	Remaud-Siméon	Glucansucrases: structure, mechanism, engineering	64
2006	Van Hijum	Glucansucrases (and fructansucrases): structure-function relationship	65
2013	Leemhuis	Glucansucrases: structure, mechanism, products, applications	66
2016	Meng	Glucansucrases, α-glucanotransferases: structure-function relationships, evolution	67
2016	Moulis	Branching sucrases (and amylosucrases)	68
2018	Gangoiti	α-Glucanotransferases: biotechnological applications	69
2018	Miao	α-Glucanotransferases	70
2020	Li	α-Glucans: structure, properties, applications	71
2020	Gangoiti	Starch-derived α-glucans: synthesis, applications	72
2021	Molina	Glucansucrases, branching sucrases: structure-function relationship,engineeering	73
2022	Jurášková	LAB exopolysaccharides: synthesis, applications	74
2022	Yu	Glucansucrases: structure, properties, applications	75

Table S2. Chronological overview of review papers dealing with GH70 enzymes and their products.

References

1. Pijning, T.; Vujičić-Žagar, A.; Kralj, S.; Eeuwema, W.; Dijkhuizen, L.; Dijkstra, B.W. Biochemical and crystallographic characterization of a glucansucrase from *Lactobacillus reuteri* 180. *Biocat Biotrans.* **2008,** *26,* 12-17.

2. Vujičić-Žagar, A.; Pijning, T.; Kralj, S.; Lopez, C.A.; Eeuwema, W.; Dijkhuizen, L.; Dijkstra, B.W. Crystal structure of a 117 kDa glucansucrase fragment provides insight into evolution and product specificity of GH70 enzymes. *Proc. Natl. Acad. Sci. U. S. A.* **2010,** *107,* 21406-21411.

3. Rühmkorf, C.; Bork, C.; Mischnick, P.; Rübsam, H.; Becker, T.; Vogel, R.F. Identification of Lactobacillus curvatus TMW 1.624 dextransucrase and comparative characterization with *Lactobacillus reuteri* TMW 1.106 and *Lactobacillus animalis* TMW 1.971 dextransucrases. *Food Microbiol.* **2013,** *34,* 52-61.

4. Kralj, S.; van Geel-Schutten, G.H.; van der Maarel, M.J.; Dijkhuizen, L. Biochemical and molecular characterization of *Lactobacillus reuteri* 121 reuteransucrase. *Microbiology* **2004,** *150,* 2099-2112.

5. Pijning, T.; Vujičić-Žagar, A.; Kralj, S.; Dijkhuizen, L.; Dijkstra, B.W. Structure of the alpha-1,6/alpha-1,4-specific glucansucrase GTFA from *Lactobacillus reuteri* 121. *Acta Crystallogr. Sect. F. Struct. Biol. Cryst. Commun.* **2012,** *68,* 1448-1454.

6. Kralj, S.; van Geel-Schutten, G.H.; Rahaoui, H.; Leer, R.J.; Faber, E.J.; van der Maarel, M.J.; Dijkhuizen, L. Molecular characterization of a novel glucosyltransferase from *Lactobacillus reuteri* strain 121 synthesizing a unique, highly branched glucan with alpha-(1-->4) and alpha-(1-->6) glucosidic bonds. *Appl. Environ. Microbiol.* **2002,** *68,* 4283-4291.

7. Kralj, S.; Stripling, E.; Sanders, P.; van Geel-Schutten, G.H.; Dijkhuizen, L. Highly hydrolytic reuteransucrase from probiotic *Lactobacillus reuteri* strain ATCC 55730. *Appl Environ Microbiol.* **2005,** *71,* 3942-3950.

8. Kralj, S.; van Geel-Schutten, G.H.; Dondorff, M.M.G.; Kirsanovs, S.; van der Maarel, M. J. E. C.; Dijkhuizen, L. Glucan synthesis in the genus *Lactobacillus*: isolation and characterization of glucansucrase genes, enzymes and glucan products from six different strains. *Microbiology (Reading)* **2004,** *150,* 3681-3690.

9. Vidal, R.F.; Martínez, A.; Moulis, C.; Escalier, P.; Morel, S.; Remaud-Siméon, M.; Monsan, P. A novel dextransucrase is produced by *Leuconostoc citreum* strain B/110-1-2: an isolate used for the industrial production of dextran and dextran derivatives. *J Ind Microbiol Biotechnol.* **2011,** *38,* 1499-1506.

10. Neubauer, H.; Bauché, A.; Mollet, B. Molecular characterization and expression analysis of the dextransucrase DsrD of *Leuconostoc mesenteroides* Lcc4 in homologous and heterologous Lactococcus lactis cultures. *Microbiology (Reading)* **2003,** *149,* 973-982.

11. Siddiqui, N.N.; Aman, A.; Qader, S.A.U. Mutational analysis and characterization of dextran synthesizing enzyme from wild and mutant strain of *Leuconostoc mesenteroides*. *Carbohydr Polym.* **2013,** *91*, 209-216.

12. Zhang, H.; Hu, Y.; Zhu, C.; Zhu, B.; Wang, Y. Cloning, sequencing and expression of a dextransucrase gene (dexYG) from *Leuconostoc mesenteroides*. *Biotechnol Lett.* **2008,** *30,* 1441-1446.

13. Yalin, Y.; Jin, L.; Jianhua, W.; Da, T.; Zigang, T. Expression and characterization of dextransucrase gene dsrX from Leuconostoc mesenteroides in Escherichia coli. *J Biotechnol* **2008,** *133,* 505-512.

14. Monchois, V.; Remaud-Siméon, M.; Russell, R.R.; Monsan, P.; Willemot, R.M. Characterization of Leuconostoc mesenteroides NRRL B-512F dextransucrase (DSRS) and identification of amino-acid residues playing a key role in enzyme activity. *Appl Microbiol Biotechnol.* **1997,** *48*, 465-472.

15. Kim, Y.; Yeon, M.J.; Choi, N.; Chang, Y.; Jung, M.Y.; Song, J.J.; Kim, J.S. Purification and characterization of a novel glucansucrase from *Leuconostoc lactis* EG001. *Microbiol Res.* **2010,** *165,* 384-391.

16. Yi, A.; Lee, S.; Jang, M.; Park, J.; Eom, H.; Han, N.S.; Kim, T. Cloning of dextransucrase gene from *Leuconostoc citreum* HJ-P4 and its high-level expression in E. coli by low temperature induction. *J Microbiol Biotechnol.* **2009,** *19,* 829-835.

17. Kang, H.; Kim, Y.; Kim, D. Functional, genetic, and bioinformatic characterization of dextransucrase (DSRBCB4) gene in *Leuconostoc mesenteroides* B-1299CB4. *J Microbiol Biotechnol.* **2008,** *18,* 1050-1058.

18. Yoon, S.; Fulton, D.B.; Robyt, J.F. Enzymatic synthesis of L-DOPA alpha-glycosides by reaction with sucrose catalyzed by four different glucansucrases from four strains of *Leuconostoc mesenteroides. Carbohydr Res.* **2010,** *345,* 1730-1735.

19. Monchois, V.; Remaud-Siméon, M.; Monsan, P.; Willemot, R.M. Cloning and sequencing of a gene coding for an extracellular dextransucrase (DSRB) from *Leuconostoc mesenteroides* NRRL B-1299 synthesizing only a alpha (1-6) glucan. *FEMS Microbiol Lett.* **1998,** *159,* 307-315.

20. Yoon, S.; Bruce Fulton, D.; Robyt, J.F. Enzymatic synthesis of two salicin analogues by reaction of salicyl alcohol with Bacillus macerans cyclomaltodextrin glucanyltransferase and *Leuconostoc mesenteroides* B-742CB dextransucrase. *Carbohydr Res.* **2004,** *339,* 1517-1529

21. Vuillemin, M.; Grimaud, F.; Claverie, M.; Rolland-Sabaté, A.; Garnier, C.; Lucas, P.; Monsan, P.; Dols-Lafargue, M.; Remaud-Siméon, M.; Moulis, C. A dextran with unique rheological properties produced by the dextransucrase from *Oenococcus kitaharae* DSM 17330. *Carbohydr Polym.* **2018,** *179,* 10-18.

22. Kajala, I.; Shi, Q.; Nyyssölä, A.; Maina, N.H.; Hou, Y.; Katina, K.; Tenkanen, M.; Juvonen, R. Cloning and characterization of a *Weissella confusa* dextransucrase and its application in high fibre baking. *PLoS One* **2015,** *10,* e0116418.

23. Amari, M.; Arango, L.F.G.; Gabriel, V.; Robert, H.; Morel, S.; Moulis, C.; Gabriel, B.; Remaud-Siméon, M.; Fontagné-Faucher, C. Characterization of a novel dextransucrase from *Weissella confusa* isolated from sourdough. *Appl Microbiol Biotechnol* **2013,** *97,* 5413-5422.

24. Shukla, S.; Shi, Q.; Maina, N.H.; Juvonen, M.; Maijatenkanen, n.; Goyal, A. Weissella confusa Cab3 dextransucrase: properties and in vitro synthesis of dextran and glucooligosaccharides. *Carbohydr Polym.* **2014,** *101,* 554-564.

25. Bounaix, M.S.; Robert, H.; Gabriel, V.; Morel, S.; Remaud-Siméon, M.; Gabriel, B.; Fontagne-Faucher, C. Characterization of dextran-producing *Weissella* strains isolated from sourdoughs and evidence of constitutive dextransucrase expression. *FEMS Microbiol. Lett.* **2010,** *311,* 18-26.

26. Kang, H.; Oh, J.; Kim, D. Molecular characterization and expression analysis of the glucansucrase DSRWC from *Weissella cibaria* synthesizing a alpha(1-->6) glucan. *FEMS Microbiol Lett.* **2009,** *292,* 33-41.

27. Ko, J.; Jeong, H.J.; Ryu, Y.B.; Park, S.; Wee, Y.; Kim, D.; Kim, Y.; Lee, W.S. Large increase in *Leuconostoc citreum* KM20 dextransucrase activity achieved by changing the strain/inducer combination in an E. coli expression system. *J Microbiol Biotechnol.* **2012,** *22,* 510-515.

28. Monchois, V.; Willemot, R.M.; Remaud-Siméon, M.; Croux, C.; Monsan, P. Cloning and sequencing of a gene coding for a novel dextransucrase from *Leuconostoc mesenteroides* NRRL B-1299 synthesizing only alpha (1-6) and alpha (1-3) linkages. *Gene* **1996,** *182,* 23-32.

29. Chellapandian, M.; Larios, C.; Sanchez-Gonzalez, M.; Lopez-Munguia, A. Production and properties of a dextransucrase from *Leuconostoc mesenteroides* IBT-PQ isolated from 'pulque', a traditional Aztec alcoholic beverage. *J. Ind. Microbiol. Biotechnol.* **1998,** *21,* 51-56.

30. Tsumori, H.; Minami, T.; Kuramitsu, H.K. Identification of essential amino acids in the Streptococcus mutans glucosyltransferases. *J Bacteriol.* **1997,** *179,* 3391-3396.

31. Schormann, N.; Patel, M.; Thannickal, L.; Purushotham, S.; Wu, R.; Mieher, J.L.; Wu, H.; Deivanayagam, C. The catalytic domains of *Streptococcus mutans* glucosyltransferases: a structural analysis. *Acta Cryst F.* **2023,** *79,* 119-127.

32. Ueda, S.; Shiroza, T.; Kuramitsu, H.K. Sequence analysis of the gtfC gene from *Streptococcus mutans* GS-5. *Gene* **1988,** *69,* 101.

33. Ito, K.; Ito, S.; Shimamura, T.; Weyand, S.; Kawarasaki, Y.; Misaka, T.; Abe, K.; Kobayashi, T.; Cameron, A.D.; Iwata, S. Crystal structure of glucansucrase from the dental caries pathogen *Streptococcus mutans*. *J Mol Biol.* **2011,** *408,* 177-186.

34. Mukasa, H.; Shimamura, A.; Tsumori, H. Nigerooligosaccharide acceptor reaction of *Streptococcus sobrinus* glucosyltransferase GTF-I. *Carbohydr Res.* **2000,** *326,* 98-103.

35. Monchois, V.; Arguello-Morales, M.; Russell, R.R. Isolation of an active catalytic core of *Streptococcus downei* MFe28 GTF-I glucosyltransferase. *J. Bacteriol.* **1999,** *181,* 2290-2292.

36. Hanada, N.; Fukushima, K.; Nomura, Y.; Senpuku, H.; Hayakawa, M.; Mukasa, H.; Shiroza, T.; Abiko, Y. Cloning and nucleotide sequence analysis of the *Streptococcus sobrinus* gtfU gene that produces a highly branched water-soluble glucan. *Biochim Biophys Acta* **2002,** *1570,* 75-79.

37. Giffard, P.M.; Simpson, C.L.; Milward, C.P.; Jacques, N.A. Molecular characterization of a cluster of at least two glucosyltransferase genes in *Streptococcus salivarius* ATCC 25975. *J Gen Microbiol.* **1991,** *137,* 2577-2593.

38. Simpson, C.L.; Cheetham, N.W.H.; Jacques, N.A. Four glucosyltransferases, GtfJ, GtfK, GtfL and GtfM, from *Streptococcus salivarius* ATCC 25975. *Microbiology (Reading)* **1995,** *141 (Pt 6),* 1451-1460.

39. Shimamura, A.; Nakano, Y.J.; Mukasa, H.; Kuramitsu, H.K. Identification of amino acid residues in *Streptococcus mutans* glucosyltransferases influencing the structure of the glucan product. *J Bacteriol.* **1994,** *176,* 4845-4850.

40. Wittrock, S.; Swistowska, A.M.; Collisi, W.; Hofmann, B.; Hecht, H.; Hofer, B. Re- or displacement of invariant residues in the C-terminal half of the catalytic domain strongly affects catalysis by glucosyltransferase R. *FEBS Lett.* **2008,** *582,* 491-496.

41. Vickerman, M.M.; Clewell, D.B. Deletions in the carboxyl-terminal region of *Streptococcus gordonii* glucosyltransferase affect cell-associated enzyme activity and sucrose-associated accumulation of growing cells. *Appl Environ Microbiol.* **1997,** *63,* 1667-1673.

42. Gilmore, K.S.; Russell, R.R.; Ferretti, J.J. Analysis of the *Streptococcus downei* gtfS gene, which specifies a glucosyltransferase that synthesizes soluble glucans. *Infect Immun.* **1990,** *58,* 2452-2458.

43. Passerini, D.; Vuillemin, M.; Ufarté, L.; Morel, S.; Loux, V.; Fontagné-Faucher, C.; Monsan, P.; Remaud-Siméon, M.; Moulis, C. Inventory of the GH70 enzymes encoded by *Leuconostoc citreum* NRRL B-1299 - identification of three novel α-transglucosylases. *FEBS J.* **2015,** *282,* 2115-2130.

44. Claverie, M.; Cioci, G.; Vuillemin, M.; Monties, N.; Roblin, P.; Lippens, G.; Remaud-Siméon, M.; Moulis, C. Investigations on the determinants responsible for low molar mass dextran formation by DSR-M dextransucrase. *ACS Catal.* **2017,** *7,* 7106-7119.

45. Bozonnet, S.; Dols-Laffargue, M.; Fabre, E.; Pizzut, S.; Remaud-Siméon, M.; Monsan, P.; Willemot, R. Molecular characterization of DSR-E, an alpha-1,2 linkage-synthesizing dextransucrase with two catalytic domains. *J Bacteriol.* **2002,** *184,* 5753-5761.

46. Brison, Y.; Pijning, T.; Malbert, Y.; Fabre, E.; Mourey, L.; Morel, S.; Potocki-Veronese, G.; Monsan, P.; Tranier, S.; Remaud-Siméon, M.; Dijkstra, B.W. Functional and structural characterization of alpha-(1->2) branching sucrase derived from DSR-E glucansucrase. *J. Biol. Chem.* **2012,** *287,* 7915-7924.

47. Wangpaiboon, K.; Padungros, P.; Nakapong, S.; Charoenwongpaiboon, T.; Rejzek, M.; Field, R.A.; Pichyangkura, R. An α-1,6-and α-1,3-linked glucan produced by *Leuconostoc citreum* ABK-1 alternansucrase with nanoparticle and film-forming properties. *Sci Rep.* **2018,** *8,* 8340.

48. Argüello-Morales, M.A.; Remaud-Siméon, M.; Pizzut, S.; Sarçabal, P.; Willemot, R.; Monsan, P. Sequence analysis of the gene encoding alternansucrase, a sucrose glucosyltransferase from *Leuconostoc mesenteroides* NRRL B-1355. *FEMS Microbiol Lett.* **2000,** *182,* 81-85.

49. Molina, M.; Moulis, C.; Monties, N.; Pizzut-Serin, S.; Guieysse, D.; Morel, S.; Cioci, G.; Remaud-Siméon, M. Deciphering an undecided enzyme: investigations of the structural determinants involved in the linkage specificity of alternansucrase. *ACS Catal.* **2019,** *9,* 2222-2237.

50. Yang, W.; Sheng, L.; Chen, S.; Wang, L.; Su, L.; Wu, J. Characterization of a new 4,6-α-glucanotransferase from *Limosilactobacillus fermentum* NCC 3057 with ability of synthesizing low molecular mass isomalto-/maltopolysaccharide. *Food Bioscience* **2022,** *46,* 101514.

51. Leemhuis, H.; Dijkman, W.P.; Dobruchowska, J.M.; Pijning, T.; Grijpstra, P.; Kralj, S.; Kamerling, J.P.; Dijkhuizen, L. 4,6-alpha-Glucanotransferase activity occurs more widespread in *Lactobacillus* strains and constitutes a separate GH70 subfamily. *Appl. Microbiol. Biotechnol.* **2013,** *97,* 181-193.

52. Gangoiti, J.; van Leeuwen, S.S.; Meng, X.; Duboux, S.; Vafiadi, C.; Pijning, T.; Dijkhuizen, L. Mining novel starch-converting glycoside hydrolase 70 enzymes from the Nestlé Culture Collection genome database: The *Lactobacillus reuteri* NCC 2613 GtfB. *Sci. Rep.* **2017,** *7,* 9947-16.

53. Pijning, T.; Gangoiti, J.; Te Poele, E.M.; Börner, T.; Dijkhuizen, L. Insights into broad-specificity starch modification from the crystal structure of *Limosilactobacillus reuteri* NCC 2613 4,6-α-glucanotransferase GtfB. *J Agric Food Chem* **2021,** *69,* 13235-13245.

54. Gangoiti Muñecas, J.; van Leeuwen, S.S.; Gerwig, G.J.; Duboux, S.; Vafiadi, C.; Pijning, T.; Dijkhuizen, L. 4,3-α-Glucanotransferase, a novel reaction specificity in glycoside hydrolase family 70 and clan GH-H. *Sci. Rep.* **2017,** *7,* 1-15.

55. Dobruchowska, J.M.; Gerwig, G.J.; Kralj, S.; Grijpstra, P.; Leemhuis, H.; Dijkhuizen, L.; Kamerling, J.P. Structural characterization of linear isomalto-/malto-oligomer products synthesized by the novel GTFB 4,6-α-glucanotransferase enzyme from *Lactobacillus reuteri* 121. *Glycobiology* **2012,** *22,* 517-528.

56. Bai, Y.; Gangoiti, J.; Dijkstra, B.W.; Dijkhuizen, L.; Pijning, T. Crystal Structure of 4,6-α-Glucanotransferase supports diet-driven evolution of GH70 enzymes from α-amylases in oral bacteria. *Structure* **2017,** *25*, 231-242.

57. Te Poele, E.M.; van der Hoek, S.E.; Chatziioannou, A.C.; Gerwig, G.J.; Duisterwinkel, W.J.; Oudhuis, L.A.A.C.M.; Gangoiti, J.; Dijkhuizen, L.; Leemhuis, H. GtfC Enzyme of *Geobacillus* sp. 12AMOR1 represents a novel thermostable type of GH70 4,6-α-glucanotransferase that synthesizes a linear alternating (α1 → 6)/(α1 → 4) α-glucan and delays bread staling. *J. Agric. Food Chem.* **2021,** *69,* 9859-9868.

58. Pijning, T.; Te Poele, E.M.; de Leeuw, T.C.; Guskov, A.; Dijkhuizen, L. Crystal structure of 4,6-α-glucanotransferase GtfC-ΔC from thermophilic *Geobacillus* 12AMOR1: starch transglycosylation in non-permuted GH70 Enzymes. *J Agric Food Chem.* **2022,** *70,* 15283-15295.

59. Gangoiti, J.; Pijning, T.; Dijkhuizen, L. The *Exiguobacterium sibiricum* 255-15 GtfC enzyme represents a novel glycoside hydrolase 70 subfamily of 4,6-α-glucanotransferase enzymes. *Appl. Environ. Microbiol.* **2015,** *82*, 756-766.

60. Gangoiti, J.; Lamothe, L.; van Leeuwen, S.S.; Vafiadi, C.; Dijkhuizen, L. Characterization of the *Paenibacillus beijingensis* DSM 24997 GtfD and its glucan polymer products representing a new glycoside hydrolase 70 subfamily of 4,6-α-glucanotransferase enzymes. *PLoS One* **2017,** *12,* e0172622.

61. Gangoiti, J.; van Leeuwen, S.S.; Vafiadi, C.; Dijkhuizen, L. The Gram-negative bacterium *Azotobacter chroococcum* NCIMB 8003 employs a new glycoside hydrolase family 70 4,6-α-glucanotransferase enzyme (GtfD) to synthesize a reuteran like polymer from maltodextrins and starch. *Biochim. Biophys. Acta* **2016,** *1860,* 1224-1236.

62. Dietrich, C.; Li de la Sierra-Gallay, I.; Masi, M.; Girard, E.; Dautin, N.; Constantinesco-Becker, F.; Tropis, M.; Daffé, M.; van Tilbeurgh, H.; Bayan, N. The C-terminal domain of *Corynebacterium glutamicum* mycoloyltransferase A is composed of five repeated motifs involved in cell wall binding and stability. *Mol Microbiol.* **2020,** *114,* 1-16.

63. Monchois, V.; Willemot, R.M.; Monsan, P. Glucansucrases: mechanism of action and structure-function relationships. *FEMS Microbiol Rev.* **1999,** *23,* 131-151.

64. Remaud-Siméon, M.; Albenne, C.; Joucia, G.; Fabre, E.; Bozonnet, S.; Pizzut, S.; Escalier, P.; Potocki-Vèronèse, G.; Monsan, P. Glucansucrases: structural basis, mechanistic aspects, and new perspectives for engineering, In *Oligosaccharides in Food and Agriculture*, American Chemical Society **2003**; *849*, 90-103.

65. Van Hijum, S. A.; Kralj, S.; Ozimek, L. K.; Dijkhuizen, L.; and Van Geel-Schutten, I. G. Structure-function relationships of glucansucrase and fructansucrase enzymes from lactic acid bacteria. *Microbiol Mol Biol Rev.* **2006**, *70*, 157-176.

66. Leemhuis, H.; Pijning, T.; Dobruchowska, J.M.; Van Leeuwen, S.; Dijkstra, B.W.; Dijkhuizen, L. Glucansucrases: three-dimensional structures, reactions, mechanism, α-glucan analysis and their implications in biotechnology and food applications. *J. Biotechnol.* **2013,** *163,* 250-272.

67. Meng, X.; Gangoiti, J.; Bai, Y.; Pijning, T.; Van Leeuwen, S.S.; Dijkhuizen, L. Structure-function relationships of family GH70 glucansucrase and 4,6-α-glucanotransferase enzymes, and their evolutionary relationships with family GH13 enzymes. *Cell Mol. Life Sci.* **2016,** *73,* 2681-2706.

68. Moulis, C.; André, I.; Remaud-Siméon, M. GH13 amylosucrases and GH70 branching sucrases, atypical enzymes in their respective families. *Cell Mol Life Sci.* **2016,** *73,* 2661-2679.

69. Gangoiti, J.; Pijning, T.; Dijkhuizen, L. Biotechnological potential of novel glycoside hydrolase family 70 enzymes synthesizing α-glucans from starch and sucrose. *Biotechnol Adv.* **2018,** *36,* 196-207.

70. Miao, M.; Jiang, B.; Jin, Z.; BeMiller, J.N. Microbial starch-converting enzymes: recent insights and perspectives. *Compr Rev Food Sci Food Saf.* **2018,** *17,* 1238-1260.

71. Li, X.; Wang, X.; Meng, X.; Dijkhuizen, L.; Liu, W. Structures, physico-chemical properties, production and (potential) applications of sucrose-derived α-d-glucans synthesized by glucansucrases. *Carbohydr Polym.* **2020,** *249,* 116818.

72. Gangoiti, J.; Corwin, S.F.; Lamothe, L.M.; Vafiadi, C.; Hamaker, B.R.; Dijkhuizen, L. Synthesis of novel alpha-glucans with potential health benefits through controlled glucose release in the human gastrointestinal tract. *Crit. Rev. Food Sci. Nutr.* **2020,** *60,* 123-146.

73. Molina, M.; Cioci, G.; Moulis, C.; Séverac, E.; Remaud-Siméon, M. Bacterial α-glucan and branching sucrases from GH70 family: discovery, structure–function relationship studies and engineering. *Microorganisms* **2021,** *9,* 1607.

74. Jurášková, D.; Ribeiro, S. C.; Silva, C. C. G. Exopolysaccharides produced by lactic acid bacteria: from biosynthesis to health-promoting properties. *Foods* **2022,** *11*,156.

75. Yu, L.; Qian, Z.; Ge, J.; Du, R. Glucansucrase produced by lactic acid bacteria: structure, properties, and applications. *Fermentation* **2022,** *8*, 629-648.
